# Membrane Fusion‐Based Drug Delivery Liposomes Transiently Modify the Material Properties of Synthetic and Biological Membranes

**DOI:** 10.1002/smll.202408039

**Published:** 2025-02-25

**Authors:** Jayna Hammond, Ceri J. Richards, YouBeen Ko, Thijs Jonker, Christoffer Åberg, Wouter H. Roos, Rafael B. Lira

**Affiliations:** ^1^ Moleculaire Biofysica Zernike Instituut Rijksuniversiteit Groningen Groningen The Netherlands; ^2^ Pharmaceutical Analysis Groningen Research Institute of Pharmacy Rijksuniversiteit Groningen Groningen The Netherlands; ^3^ Present address: Department of Bionanoscience Kavli Institute of Nanoscience Delft University of Technology Delft The Netherlands

**Keywords:** drug‐delivery, FLIM, giant unilamellar vesicles, membrane fusion, membranes

## Abstract

Many drug targets are located in intracellular compartments of cells but they often remain inaccessible to standard imaging and therapeutic agents. To aid intracellular delivery, drug carrier nanoparticles have been used to overcome the barrier imposed by the plasma membrane. The carrier must entrap large amounts of cargo, efficiently and quickly deliver the cargo in the cytosol or other intracellular compartments, and must be inert; they should not induce cellular responses or alter the cell state in the course of delivery. This study demonstrates that cationic liposomes with high charge density efficiently fuse with synthetic membranes and the plasma membrane of living cells. Direct fusion efficiently delivers large amounts of cargo to cells and cell‐like vesicles within seconds, bypassing slow and often inefficient internalization‐based pathways. These effects depend on liposome charge density, concentration, and the helper lipid. However, fusion‐mediated cargo delivery results in the incorporation of large amounts of foreign lipids, causing changes to the material properties of these membranes, namely modifications in membrane packing and fluidity, induction of membrane curvature, decrease in surface tension, and the formation of (short‐lived) pores. Importantly, these effects are transient and liposome removal allows cells to recover their state prior to liposome interaction.

## Graphical abstract

The table of content summarizes the main development and findings of this study. Fusion of small liposomes with acceptor model or biomembranes modify the material properties of these membranes. In sketch 1, several liposomes interacting with an acceptor model vesicle. A single fusion event is shown and highlighted in sketch 2, showing lipid and content mixing. Sketches 3 and 4 show content mixing results in transfer of entrapped cargo to lipid vesicles and living cells, respectively. Docked liposomes are shown (arrows). In sketch 5, FLIM images showing that fusion fluidizes cell membranes and internalizes excess of lipids. In sketch 6, the arrows point to the labeled plasma membrane after liposome fusion with cells.

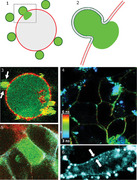

## 1 Introduction

Intracellular delivery of water‐soluble cargos using nanosized carriers has long been envisioned as a way to efficiently deliver various biologically‐active molecules, nanoparticles, and genes into cells. Dependent on their design, nanocarriers can offer controlled and targeted release,^[^ ^1^, ^2^ ^]^ high biocompatibility,^[^ ^3^ ^]^ improved cargo solubility,^[^ ^4^ ^]^ combined imaging and therapeutic modality cargos,^[^ ^5^ ^]^ as well as cargo co‐delivery.^[^ ^6^, ^7^ ^]^ Furthermore, they can prevent cargo degradation,^[^ ^8^ ^]^ and enhance cellular uptake.^[^ ^9^, ^10^ ^]^ Among the various delivery vehicles are liposomes: vesicles made of lipids whose fundamental building blocks are similar to the main building blocks of cellular membranes. As delivery systems, they offer large versatility, can encapsulate both hydrophilic and hydrophobic particles at high yield, are easy to produce, purify, and functionalize, and their mechanical properties—which often dictate their interaction with the target system^[^ ^11^ ^]^—can be easily tuned by the choice of their lipid constituents.^[^ ^12^, ^13^ ^]^ An important feature of drug delivery systems is that they are usually designed to be inert, functioning solely as a vehicle for cargo delivery without interfering with any aspect of the target system. However, particles are seldom completely inert. For in vivo delivery, incompatible particles may induce an immune response, potentially leading to adverse side effects. For in vitro applications such as cell transfection, it is important that the cells remain alive and functioning for further usage (i.e., ex vivo transfection and cellular reintroduction). Commonly, nanobiocompatibility is assessed by determining cellular viability, cytotoxicity, or proliferation after exposure to particles.^[^ ^3^ ^]^ However, the effects of nanoparticle carriers on the material properties of the target system has been generally overlooked.

In general, nanoparticles enter cells via endocytosis, or in some instances, via direct penetration through the cell membrane.^[^ ^14^, ^15^ ^]^ However, both delivery methods have their drawbacks.^[^ ^16^ ^]^ In the case of endocytosis, the particles are typically trafficked through the endo‐lysosomal pathway, introducing the additional challenge of releasing the drug from the endo/lysosomes into the cell cytosol before degradation or exocytosis.^[^ ^14^, ^15^ ^]^ Particles that enter through direct penetration, on the other hand, may have off‐site delivery and may induce damage to the cell plasma membrane (PM).^[^ ^17^, ^18^ ^]^ In contrast, a specific class of liposomes that deliver their cargo via fusion with the PM, called fusogenic liposomes, are able to directly deliver cargos into the cell cytosol whilst maintaining targeting capacity.^[^ ^19^, ^20^ ^]^ The direct fusion of liposomes inevitably involves the transfer of the lipid content from the liposome to the cell PM. This potentially induces changes in the properties of the PM, either locally at the fusion site, or more globally across the entire cell. Which and to what degree the PM material properties change due to membrane mixing in the course of drug delivery, whether the effects are adverse, and finally if they are reversible are important questions that have remained elusive. Moreover, for in vivo nanocarrier applications, the adsorption of environmental proteins onto the carrier surface, known as biomolecular corona formation,^[^ ^21^ ^]^ has been shown to mitigate toxicity in many systems,^[^ ^22^ ^]^ although in some cases it may reduce targeting ability^[^ ^23^, ^24^ ^]^ or prevent delivery altogether.^[^ ^25^, ^26^, ^27^ ^]^ Thus, the effect of environmental proteins on the fusion process, and hence on intracellular delivery of cargos, and any induced cellular changes are important factors to consider, but remain largely unexplored.

Here, we set out to investigate whether liposome nanovehicles, containing or devoid of cargo, pose short‐ or long‐term effects on acceptor synthetic and biological membranes. For this, we use cationic fusogenic liposomes that readily (thousands per cell) and quickly (within seconds) fuse with acceptor membranes, efficiently delivering their encapsulated cargo. To assess the specific effects of charge or chemical composition of the lipid formulation, we use either mono or multivalent cationic lipids with varying liposome charge density, containing cylindrical or cone‐shaped helper lipids. We used multicolor confocal microscopy and fluorescence lifetime imaging microscopy (FLIM) to assess fusion efficiency and the effects of fusion on the material properties of membranes, including changes of membrane fluidity, membrane remodeling and formation of leakage pores. As model systems, we used giant unilamellar vesicles (GUVs) that are devoid of metabolism, receptors or other active processes, so the physical–chemical effects on membranes are assessed without interference from underlying biological activity, as well as living cells to understand how these changes impart on biological systems.

We show that cationic liposomes exhibiting a high charge density (σ_M_), efficiently fuse with acceptor synthetic and biological membranes, efficiently delivering cargo. At this σ_M_, helper lipids become dispensable. At intermediate σ_M_, fusion efficiency is reduced despite stable membrane docking, and incorporation of inverted cone‐shaped helper lipids improves fusion efficiency. Uncharged liposomes are unable to bind and fuse. Interestingly, preincubation in serum leads to protein corona formation on the liposome surface, shielding their charges and having a similar effect as low σ_M_, preventing fusion. Extensive liposome fusion modifies acceptor membrane fluidity in a way that can be predicted by lipid compositional changes, leading to changes in membrane tension, curvature, and eventually to the formation of leakage pores. Interestingly, the formed pores have specific sizes and a limited lifetime. This means that even if the PM of living cells becomes permeabilized, the delivered cargos are retained in the cytosol. Importantly, removal of liposomes from the media recovers the introduced changes in the membrane material properties.

### 1.1 Rationale

In our proto drug delivery system, strong interaction and fusion with acceptor synthetic and biological membranes dispersed in low ionic strength solutions requires charge. To enable a strong interaction, and thereby a higher probability of fusion between the liposome and acceptor membranes, we decided to use positively charged liposomes. Admittedly, electrostatic interactions are highly screened in high ionic strength solutions such as serum, but the interaction should nevertheless remain strong at short range. Furthermore, ex vivo experiments can readily be performed under low ionic (but isotonic) conditions, as done here, where the electrostatic interactions will remain strong. To render the liposomes positively charged, their membranes contained the cationic lipid DOTAP. DOTAP is a cylindrically shaped lipid that forms bilayers, and hence liposomes can be made of purely DOTAP lipids. Furthermore, it is well known that the structure and activity of liposomes also depends on helper lipids, conferring liposome stability, determining phase state, and restructuring upon interaction with target membranes.^[^ ^28^, ^29^ ^]^ The most commonly used helper lipids are DOPC and DOPE. Due to its cylindrical shape, DOPC forms lamellar phase,^[^ ^30^ ^]^ and thus, liposomes can also be made purely of DOPC. In contrast, DOPE is an inverted cone‐shaped lipid that imposes negative membrane curvature, and DOPE alone or with low fractions of cylindrical lipids forms inverted hexagonal phases.^[^ ^31^ ^]^ Depending on the helper lipid, liposomes exhibit different modes of interactions with cells, with mixtures of DOTAP:DOPE efficiently fusing with the cell plasma membrane, whereas DOTAP:DOPC liposomes are internalized by cells.^[^ ^32^, ^33^ ^]^

**Figure 1:**
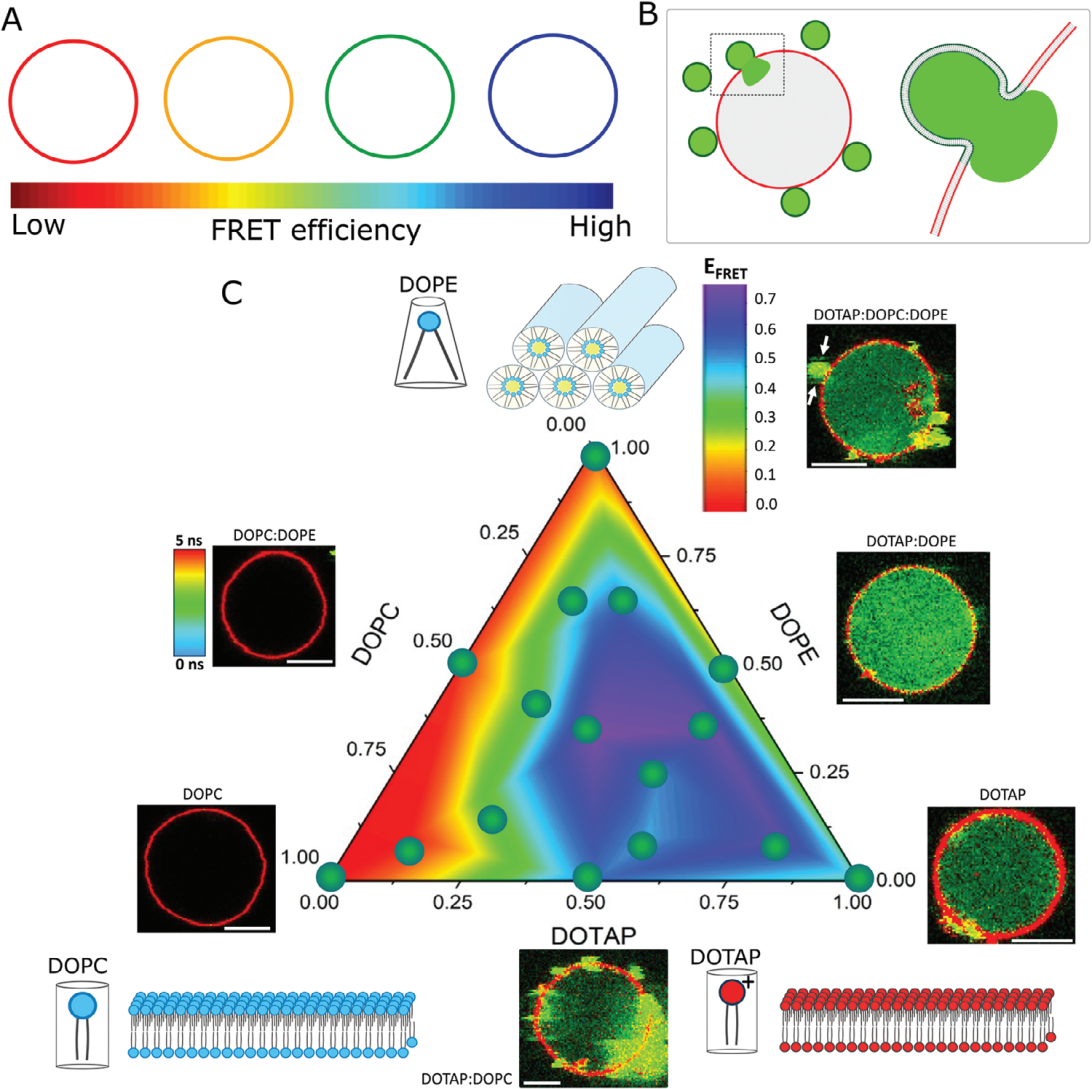
Fusion efficiency diagram for three‐component liposomes. A) FLIM‐FRET sketch of the fusion assay. Donor lifetime decreases (red to blue colors) as fusion efficiency increases, as assessed from an increase in FRET efficiency (*E*_FRET_). B) Sketch of the content mixing assay in which liposomes encapsulating a water‐soluble probe (green) fully fuse with an acceptor GUV (red), transferring its content to the GUV lumen. C) *E*_FRET_ fusion diagram measured on GUVs incubated with liposomes containing varying fractions of DOPC, DOPE, and DOTAP. The sketches show that DOPC alone forms neutral bilayers (lower left), DOTAP alone forms positively charged bilayers (lower right), whereas DOPE alone forms an inverted hexagonal phase (top) due to its molecular geometry. The heat map within the ternary component diagram represents the estimated fusion efficiency based on *E*_FRET_ measurements. Each green solid circle represents the mean of a tested liposomal composition (10–15 GUVs per liposome composition). *Insets*: GUV FLIM images show representative content mixing results from incubation of GUVs with the respective liposomal compositions. Here, unlabeled liposomes encapsulating 50 µM SRB fuse with Bodipy C_16_‐containing GUVs. Bodipy C_16_ and SRB are clearly resolved in the FLIM images due to their lifetime differences. Note that in some of the images, docked liposomes can be observed (arrows). Scale bar: 8 µm. The GUVs are made of DOPC:PS:Chol:DOPE (25:25:25:25, mole fraction).

To separately determine the roles of membrane charge and of the helper lipid on the interaction and fusion of liposomes, we used giant unilamellar vesicles (GUVs) as a model of the PM. The GUVs were composed of equimolar mixtures of DOPC:PS:Chol:DOPE, in which PS stands for brain phosphatidylserine (or alternatively, DOPG was used instead of PS without measurable differences), as this composition closely mimics some mechanical properties of the PM, including fluidity and charge.^[^ ^34^, ^35^ ^]^ Interaction was studied with the recently developed time‐resolved fluorescence resonance energy transfer assay.^[^ ^36^ ^]^ In this assay, fluorescence resonance energy transfer (FRET) is assessed with fluorescence lifetime imaging microscopy (FLIM). GUVs are doped with the donor Bodipy C_16_ probe, and the liposomes are made with the acceptor lipid Rh‐PE. Bodipy C_16_ is nearly insensitive to the environment,^[^ ^37^ ^]^ and thus, lifetime changes are only associated with FRET. Fusion of liposomes with the GUVs results in an increase in donor–acceptor proximity and a reduction in donor lifetime due to FRET, whereas binding only causes minimal FRET changes.^[^ ^38^ ^]^ To resolve if the liposomes fully fused with the GUVs, the liposomes encapsulated the water‐soluble dye sulforhodamine B (SRB). Upon complete merging (full fusion) of the GUV and liposomal membranes, SRB is transferred to the GUV lumen. As SRB does not spontaneously cross the membranes of GUVs or cells, its presence in the GUV lumen is a result of membrane fusion‐mediated delivery. On the other hand, the appearance of SRB‐containing liposomes on the GUV surface without content mixing is an indication of liposome binding without fusion. Therefore, in this configuration, FLIM‐FRET is able to resolve membrane docking, hemifusion and full‐fusion at the single vesicle level (**Figure** **1**).

### 1.2 Fusion Efficiency is Determined by Membrane Charge and Modulated by Helper Lipids

To independently assess the effects of membrane charge and cylindrical or inverted cone‐shaped helper lipids, we varied the mole fraction of DOPC, DOTAP, and DOPE in the liposomes. To assess fusion efficiency, we measured FRET efficiency (*E*_FRET_) at the single GUV level. In this manner, we were able to construct a liposomes composition fusion diagram for which FRET is used as the readout of fusion efficiency (Figure 1). We started with the single component compositions. As expected, liposomes made of the neutral DOPC alone do not fuse with the GUVs (*E*_FRET_ ∼ 0). DOPE alone does not form bilayers, and the hexagonal phase lipid aggregates mixed with the GUVs also do not induce any change in FRET. In contrast, liposomes made purely of DOTAP efficiently fuse with the GUVs. These results show that charge is necessary and sufficient for fusion. We next examined two‐component mixtures of lipids. As expected, neutral liposomes composed of DOPC:DOPE do not undergo fusion. In contrast, DOTAP mixtures with either DOPC and DOPE efficiently fuse with the GUVs. Interestingly, while DOPC does not seem to change the efficiency of fusion of DOTAP, mixtures with DOPE result in higher *E*_FRET_ compared to that of DOTAP alone. Of note, this binary composition is more efficient than any ternary mixtures of these lipids, indicating that the cylindrical DOPC lipid reduces liposome fusion efficiency. Aside from the results shown in Figure 1C, representative lipid mixing FLIM GUV images are shown in Figure S1 (Supporting Information) along with their respective donor decays. From the fusion diagram, it is clear that liposomal compositions rich in DOTAP and DOPE are the most fusogenic, whereas compositions further away from this mixture exhibit lower fusion efficiencies.

Importantly, for all compositions that exhibit high *E*_FRET_, the interaction results in the transfer of SRB from the liposomes to the GUV interior, showing that these liposomes undergo full fusion (inset GUV images in Figure 1C). The bright green signal observed in some of the GUV snapshots in Figure 1 C most likely correspond to bright out of focus liposomes filled with SRB. A typical FLIM image takes ≈40 s to capture with our settings, which is significantly slower compared to small molecules such as SRB diffusion (>100 µm^2^ s^−1^), and hence transfer into the GUV upon liposome fusion cannot be visualized in real‐time. In contrast, the compositions that do not lead to changes in *E*_FRET_ also do not lead to full fusion. In fact, liposomes made of neutral lipids alone do not bind the GUVs, indicating that they are unable to fuse because they are unable to bind in the first place. In summary, the results show that inclusion of the cationic lipid DOTAP in the liposome is required and sufficient to efficiently fuse liposomes with biomimetic membrane and the addition of the inverted cone‐shape helper lipid DOPE to the liposome further enhances fusion.

### 1.3 Charge Density, not Chemical Identity, Determines Membrane Fusion Efficiency and Fusion‐Dependent Pore Formation

The maximum σ_M_ attained with the liposomes using DOTAP as the cationic lipid (CL) is when DOTAP mole fraction (ϕ_DOTAP_) is 100%, thus devoid of helper lipids. However, higher σ_M_ could be attained with multivalent CLs at lower mole fractions, with the additional advantage that helper lipids could be added for fine tuning liposome properties. Furthermore, it has been shown that cationic liposomes can be toxic to cells^[^ ^40^, ^41^ ^]^ and have limited applicability in physiological media containing serum proteins due to charge screening and particle aggregation.^[^ ^42^ ^]^ In this section, we study the effects of liposome charge density on fusion with GUVs. For this, we increase the mole fractions of the monovalent lipid DOTAP and multivalent CL lipid MVL5 (ϕ_MVL5_), a lipid that harbors up to five positive charges depending on pH. For these formulations, we also investigated the effects of cylindrical (DOPC) or negatively‐curved (DOPE) helper lipids. Later, we assessed whether liposome fusion modifies the structure of the acceptor GUV membrane (i.e., formation of pores) and how interaction with serum proteins affects these processes.

**Figure 2:**
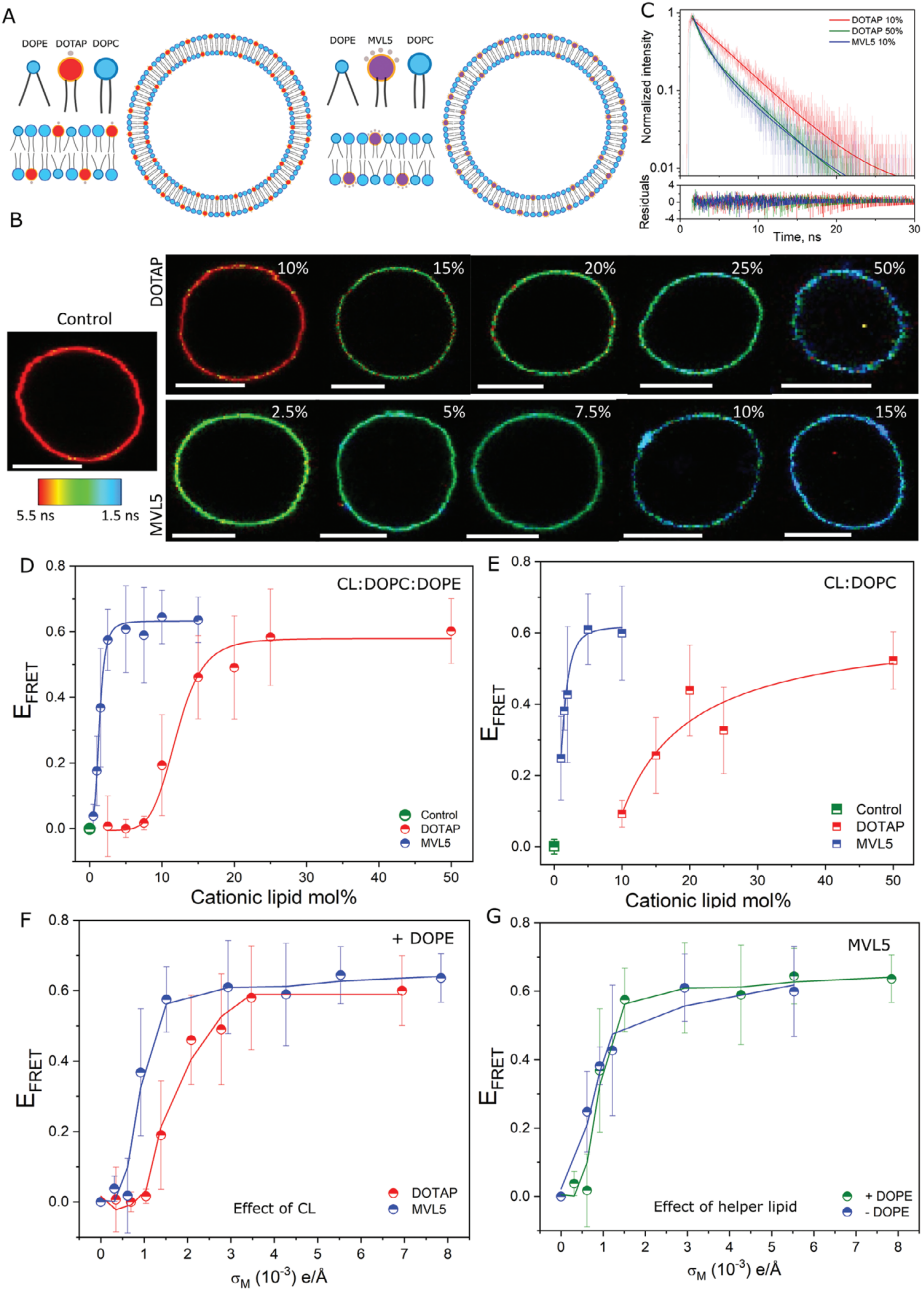
Membrane charge density is the main factor determining fusion with acceptor membranes. A) Sketch of liposomes containing the monovalent lipid DOTAP or the multivalent lipid MVL5 in the presence of helper lipids DOPC and DOPE. B) Representative FLIM GUV DOPC:PS:Chol:DOPE (25:25:25:25, mole fraction) images upon incubation with CL:DOPC:DOPE (X:50‐X:50, mole fraction) 20 µM liposomes (lipid concentration) containing increasing mol% of the CL and labeled with 2 mol% DPPE‐Rhodamine. Control GUVs in the absence of liposomes are also shown along with the lifetime map. Scale bar: 8 µm. C) Representative lifetime decays, respective fits, and residuals from the fits for GUVs labeled with FRET donor Bodipy C_16_ upon fusion with several liposome compositions. D,E) Fusion efficiency as represented by *E*_FRET_ as a function of CL mole fraction for liposomes containing DOPE and DOPC or only DOPC as the helper lipid, respectively. Control represents measurements in the absence of liposomes. Each data point represents measurements on 10–15 individual GUVs. Means and s.d. are shown. The lines are a guide to the eye. F) *E*_FRET_ as a function of the calculated charge density for liposomes containing DOTAP or MVL5 and DOPC:DOPE as the helper lipids. Liposome fusion efficiency strongly depends on σ_M_ and weakly on the chemical identity of the CL. G) *E*_FRET_ response as a function of charge density for liposomes either containing DOPE or not as the helper lipid and MVL5 as the CL. The response does not have a strong dependence on the helper lipid.

We prepared liposomes with increasing mole fractions of the CL (ϕ_CL_) DOTAP or MVL5, containing DOPC alone or DOPC:DOPE mixtures as the helper lipid(s) (**Figure** **2**A). These compositions are represented as CL:DOPC (X:100‐X) or CL:DOPC:DOPE (X:50‐X:50), in which X is the fraction of the CL, adjusted at the expense of DOPC. Figure 2B shows representative FLIM images of GUVs incubated with CL:DOPC:DOPE liposomes with increasing ϕ_CL_. Note that as ϕ_CL_ increases *E*_FRET_ also increases (Figure 2C). The quantitative *E*_FRET_ response curves as a function of ϕ_CL_ in the membrane for liposomes containing DOPE or not, are shown, respectively, in Figure 2D,E. At identical ϕ_CL_, *E*_FRET_ is much more pronounced with MVL5. At very low ϕ_CL_, liposomes containing DOTAP are not fusogenic, whereas fusion is readily detected with as low as 1 mol% MLV5. The fraction at which fusion is half maximum is ≈12 or 28 mol%, for DOTAP liposomes with or without DOPE, respectively, again pointing to a modulating effect of DOPE on fusion, and 1.3 mol% for MVL5 irrespective of the helper lipid. For both CL (or helper lipids), the maximum efficiency saturates. However, the saturation occurs at much lower fractions of MVL5 (≈2.5%) compared to DOTAP (≈25%). Thus, much lower amounts of multivalent CL is required to achieve maximal fusion.

At maximum fusion, the ϕ_DOTAP_ is ≈10‐fold higher than ϕ_MVL5_ despite the < 5 charge ratio between them. To isolate the effects of charge, we replotted the data as a function of σ_M_, which is given by:^[^ ^43^ ^]^

(1) $$\sigma_{M}=\frac{eZN_{cl}}{N_{nl}A_{nl}+N_{cl}A_{cl}}=\left[1-\phi_{nl}/\left(\phi_{nl}+r\phi_{cl}\right)\right]s_{cl}$$

Here, *N*_nl_ and *N*_cl_ are, respectively, the number of neutral and cationic lipids; *Z* is the valence of the CL (we used 1 and 4.5 for DOTAP and MVL5, respectively), *r* = *A*_cl_/*A*_nl_ is the area ratio of the cationic and neutral headgroups (we used 2.3 here, with *A*_nl_ being 72 Å^2^),^[^ ^43^ ^]^ and σ_CL_ is the mole fraction of the cationic lipid. The replotted *E*_FRET_ versus the calculated σ_M_ response nearly falls onto a universal curve for DOPC only (Figure S2A, Supporting Information) or with DOPC:DOPE mixtures as helper lipids (Figure 2F), irrespective of the CL. Thus, σ_M_ seems to be the determining factor for fusion, not the specific chemical identity of the CL. Therein, the role of the helper lipid is secondary to σ_M_, with comparable *E*_FRET_ changes for DOTAP or MVL5 liposomes (Figure 2G and Figure S2B, Supporting Information, respectively). At low σ_M_ (<10^−3^ e Å^−1^), fusion is negligible, whereas fusion is equally efficient at high σ_M_ (>3–4 10^−3^ e Å^−1^) for both CLs, with a mildly higher efficiency for liposomes containing DOPE, as expected from the fusion diagram in Figure 1. Importantly, in all cases with DOTAP or MVL5, and for liposomes containing either DOPE or not as the helper lipid, the interaction with GUVs is via full fusion (Figure S3, Supporting Information). The results show that it is σ_M_ and not the chemical identity of the CL that is the determining factor for fusion of cationic liposomes with acceptor membranes, with efficient delivery of cargo into GUV model membranes, and with the helper lipids having a regulatory effect on fusion at moderate σ_M_.

A disadvantage of using cationic nano(particles) as delivery vehicles is that they are known to be toxic to cells.^[^ ^18^, ^40^, ^41^, ^44^ ^]^ However, it is not known whether the toxic effects are associated with the total charge (i.e., σ_M_) that needs to be processed by cells, in which case DOTAP and MVL5 would be equally toxic, or with the number of charged molecules that need to be dealt with, in which case DOTAP would be more toxic than MVL5 at identical σ_M_. Understanding the molecular mechanisms of toxicity will help the design of efficient drug delivery vehicles with limited toxicity. As a proxy of toxicity, we investigated the effect of liposome fusion on pore formation in GUVs. Recently, we have shown that extensive fusion may lead to the formation of hydrophilic pores in GUV membranes, an effect that is suppressed by physiological levels of cholesterol.^[^ ^36^ ^]^ We labeled the liposome membranes with Atto647‐PE (cyan) as a fusion reporter, and co‐incubated these liposomes with GUVs in the presence of sulforhodamine B in the medium (SRB, red), a water‐soluble probe the can only transverse membranes through pores. The assay is able to simultaneously detect fusion, fusion‐dependent pore formation, as well as vesicle morphology. We use the degree of filling analysis to quantitatively assess the extent of disruption^[^ ^45^ ^]^ wherein values close to unity indicate that larger or longer‐lived pores (or a combination of both) are formed, a sign of more extensive disruption.

**Figure 3:**
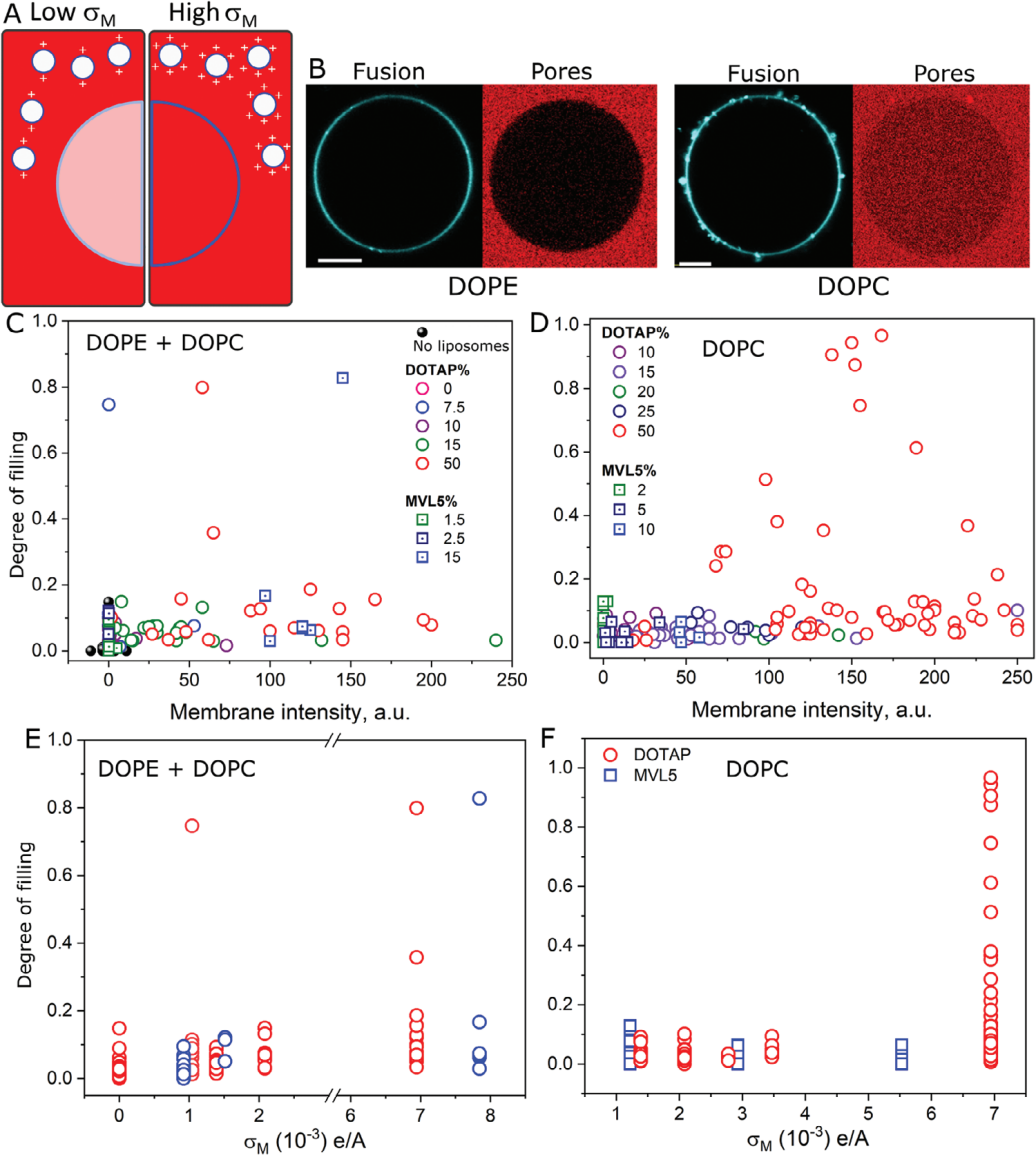
Charge density determines fusion efficiency and membrane disruption. A) Sketch of the fusion/permeabilization assay with low (left) and high (right) charge density (σ_M_) liposomes labeled at the membrane with Atto647‐DOPE (cyan) and incubated with GUVs (DOPC:DOPG:Chol:DOPE, 25:25:25:25, mole fraction) in the presence of 10 µM SRB (red) in the outer solution used as a leakage marker. More fusion (more intense membrane signal) is expected to result in more membrane permeabilization (more SRB entry). B) Representative GUV images incubated with DOTAP (50 mol%) liposomes (20 µM total lipids) either containing DOPE as the helper lipid or not (DOTAP:DOPE or DOTAP:DOPC, respectively). Scale bars: 10 µm. Note that higher fusion (more intense cyan signal at the membrane) co‐occurs with more SRB entry into the GUVs. C,D) Degree of filling dependence on fusion efficiency (measured as the intensity of Atto647‐DOPE in the GUV membrane) for DOTAP or MVL5 as the CL in liposomes containing DOPE and DOPC or only DOPC as the helper lipid, respectively. Liposome composition: CL:DOPC:DOPE (X:50‐X:50, mole fraction). The control without incubation of liposomes is also shown (no liposomes). E,F) Degree of filling in GUVs incubated with liposomes with increasing charge densities (σ_M_) for liposomes containing or not containing DOPE as the helper lipid. Each point represents a measurement on a single GUV.

We reasoned that more fusion would result in more disruption, in which case more intense membrane signal would be accompanied by more SRB leakage into the GUVs (**Figure** **3**A). Figure 3B shows two representative GUVs incubated with DOTAP‐containing liposomes (ϕ_DOTAP_ 0.5) that either do or do not contain DOPE as the helper lipid. As anticipated, more extensive fusion (stronger membrane signal) results in leakage (SRB entry). Figure 3C,D shows the effects of liposome fusion as a function of ϕ_CL_ for DOPC or DOPC:DOPE helper lipid(s). As expected, fusion becomes more efficient as ϕ_CL_ increases. Importantly, in general, the process does not lead to extensive pore formation. The only exception is the highly charged liposomes containing 50 mol% DOTAP but without DOPE. For those vesicles that do become permeable, the SRB signal in the GUV interior is lower than that in the outside medium, an indication that the formed pores reseal before full SRB equilibration. Although in general the permeabilization effects are mild, more disruptive effects such as complete GUV collapse can also be observed (Figure S4 and Movie S1, Supporting Information).^[^ ^46^ ^]^ Of note, in addition to ϕ_CL_, fusion efficiency also depends on liposomal concentration (Figure S5, Supporting Information). When we plot the degree of filling for increasing σ_M_, the GUVs only become permeable at high σ_M_, (Figure 3E,F). However, this effect again does not depend on the CL lipid used, although the presence of DOPE decreases the fraction of permeable GUVs. The results show that, similarly to the effects on fusion, the charge density, not the chemical identity of the CL, is the determining factor for fusion‐dependent membrane disruption, with the helper lipid having a tuning effect on the process. In this case, DOPE tends to both increase fusion efficiency and decrease fusion‐dependent disruption.

### 1.4 Fusion Modifies Membrane Fluidity and Curvature

Membrane fusion results in the mixing of two initially separated membranes. Thus, unless these membranes have identical compositions, fusion inevitably changes their lipid make up. Membrane physical properties are strongly tied to composition.^[^ ^12^ ^]^ Since the compositions of the liposomes and GUVs used here are not identical, we speculate that liposome fusion will not only result in changes in GUV composition, but also in their mechanics. In fact, we have previously shown that full fusion of DOTAP:DOPE liposomes to POPC:POPG GUVs induced a clear reduction in lipid diffusion in these GUVs, whereas only a milder decrease in diffusion was observed when the liposomes hemifused to neutral POPC GUVs.^[^ ^38^ ^]^ Using the Saffman–Delbrück equation^[^ ^47^ ^]^ (see methods), we calculated the approximate membrane viscosity in these membranes from the reported lipid diffusion values. The obtained values are comparable to reported FLIM data using viscosity‐sensitive molecular rotors.^[^ ^37^ ^]^ As shown in Figure S6 (Supporting Information), fusion increases the viscosity of POPC:POPG GUVs. The composition dependence is confirmed since mimetic GUVs – vesicles made of the expected composition after fusion – also exhibited increased viscosity. It has been previously reported that, in the absence of other factors, DOPE increases membrane viscosity.^[^ ^48^ ^]^ We thus interpreted these results as a consequence of fusion‐mediated incorporation of DOPE in the GUV membranes.

To confirm these observations, we used FLIM combined with molecular flippers, molecules that undergo excited‐state changes in their intensity and lifetime as a function of environmental packing,^[^ ^49^, ^50^ ^]^ as a more direct way to assess membrane packing, which relates to viscosity. As observed previously, FliptR's fluorescence decay is best fitted with a double exponential decay, in which the long lifetime (τ_long_) reports packing.^[^ ^49^, ^50^ ^]^ Figure S7 (Supporting Information) shows that liposome fusion with fluid DOPC:DOPG GUVs leads to an increase in τ_long_ (thus, an increase in packing) and this increase is weakly dependent of the CL or helper lipid. To test the generality of these observations, we studied fusion of liposomes with initially more ordered membranes rich in Chol. If the viscosity/composition dependence holds, liposome fusion would then result in a decrease rather than increase in viscosity. For this, we prepared GUV membrane models containing i) highly charged and fluid membranes, and ii) low charge and highly packed membranes, both rich in cholesterol. These two compositions mimic the lipid compositions of the inner and outer leaflet of the PM, respectively, and we have recently shown that the combination of charge and packing allows efficient fusion with the former, but not with the latter.^[^ ^36^ ^]^ Figures S8A (Supporting Information) shows representative GUVs from these compositions before and after liposome fusion, as well as mimetic GUVs with the expected composition upon complete charge neutralization. Figure S8B (Supporting Information) shows the respective fluorescence decay. As expected, fusion of liposomes with GUVs of composition i) indeed decrease τ_long_, showing a reduction in membrane packing (the lifetime ratio of liposome‐incubated/control ≈0.95), whereas liposomes interacting with GUVs of composition ii) show detectable but milder effects (≈0.98 ratio) due to the liposome reduced ability to fuse with these membranes.

In addition to the changes in fluidity, liposome fusion also modifies the spontaneous curvature of the GUVs. As can be seen in Figure 3B and Figure S9 (Supporting Information), the GUVs that underwent extensive fusion (strong membrane signal) also exhibited outward buds. This effect has been observed previously and is interpreted as a result of the transfer of the leaflet asymmetry number, inherent to small liposomes, to the otherwise flat membranes of the GUVs.^[^ ^38^ ^]^ Since membrane curvature is a physical process and occurs whenever a sufficiently large number of (small) liposomes fuse to the GUVs, bud formation should not depend on the particular liposome composition, as indeed verified (Figure S9, Supporting Information). The diameter of the bud reflects the extent of curvature – higher curvatures form smaller buds.^[^ ^51^, ^52^ ^]^ In our experiments, for DOTAP or MVL5 liposomes at identical σ_M_, either containing DOPE or not as the helper lipid, and for all concentrations tested, bud diameter does not vary significantly, as expected given their comparable fusion efficiencies. On the other hand, much higher concentrations result in complete vesicle disruption leading to GUV bursting as in Figure S4 (Supporting Information), and are thus beyond experimental range. FLIM images of FliptR‐labeled GUVs that contain buds show no visible lifetime differences in the buds compared to the flatter regions of the GUVs, indicating composition equilibration, although fitting the data from the buds alone was not possible due to the low photon count. In summary, the results show that the fluidity of the fusing membranes changes as a consequence of the changes in membrane composition, fusion transfers the intrinsic liposome leaflet number asymmetry to the GUVs, changing membrane spontaneous curvature, and lipids are fairly equally distributed in the regions of higher or low curvature.

### 1.5 Protein Corona Screens Liposome Charge and Prevents Membrane Interaction and Fusion

**Figure 4:**
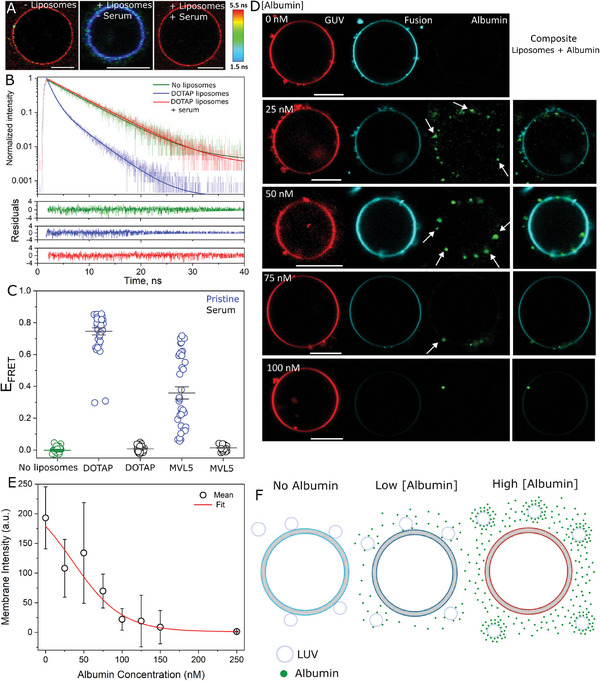
Protein‐coated cationic liposomes are unable to fuse with GUVs. A) Representative FLIM images of Bodipy C_16_‐labeled GUVs (DOPC:DOPG, 1:1 mole fraction) in the absence of liposomes, in the presence of pristine liposomes and in the presence of liposomes preincubated with serum (10× diluted). Scale bars: 9 µm. B) Bodipy C_16_ fluorescence decays along with the fits and residuals of the fits for the GUVs shown in panel A. The data from the control (– liposomes) and the liposomes preincubated in serum (+ liposomes/+ serum) were well‐fitted by a single‐exponential decay, and the data for pristine liposomes (+ liposomes/– serum) were well‐fitted by a double‐exponential decay. C) Measured *E*_FRET_ for a number of GUVs upon incubation with pristine or protein‐coated liposomes containing DOTAP (DOTAP:DOPE, 33:66 mole fraction) or MVL5 (MVL5:DOPC:DOPE, 5:50:45 mole fraction) as the CL. The control without liposomes is also shown. D) Representative GUVs (labeled with 0.5 mol% DPPE‐Rhodamine, red) upon incubation with 20 µM (lipid concentration) Atto647‐DOPE (cyan, representing fusion) liposomes that have been preincubated with increasing concentrations of FITC‐labeled albumin (green). The arrows point to protein particles or complexes with liposomes on the GUV surface. The overlay composite of liposome and albumin channels is also shown. Scale bar: 10 µm. E) Membrane signal measured from the average Atto647‐DOPE fluorescence intensity upon liposome fusion for a number of GUVs (10–15 per condition) as a function of albumin‐FITC concentration. Mean and standard deviation are shown. A fit of an exponential decay (*Y* = *A*_1_e^(‐^ *^X^* ^/^ *^t^* ^1)^+*y*_0_, where *A*_1_ and *y*_0_ are the maximum Atto647‐DOPE intensity and offset at 0 intensity, respectively) to the data is also shown. F) Sketch of the expected interactions between cationic liposomes and GUVs. In the absence of albumin, the liposomes directly fuse with the GUVs, transferring their (cyan) lipids. At intermediate albumin concentrations, partial liposome coverage reduces liposome binding but those that are bound can still fuse. At high protein concentration, complete protein coverage prevents liposome binding altogether and there is no lipid mixing.

In biological systems, media are rich in ionic species as well as large concentrations of biomacromolecules, both of which have the ability to reduce interactions, by charge screening and protein corona formation, respectively.^[^ ^44^, ^53^ ^]^ While membrane fusion of cationic liposomes and GUVs is efficient at high ionic strength,^[^ ^38^ ^]^ here we tested the effects of protein corona formation. We first preincubated the cationic liposomes with serum, and then delivered the liposome/serum dispersion to the GUVs. Thus, note that GUVs were exposed to the liposomes in the presence of both a hard and soft corona, as well as in the presence of serum proteins in the medium. Zeta potential measurements on pristine cationic liposomes show comparatively high values > 40 mV for DOTAP and MVL5 (Figure S10, Supporting Information). Incubation with serum not only neutralizes the liposome charge, but actually “overshoots” the neutralization such that the zeta potentials become negative, most likely due to dense protein coating on the particle surface. At the serum concentration tested, there are no signs of liposome aggregation irrespective of the CL used (Figure S11, Supporting Information). We used multicolor confocal and FLIM microscopy to study the effects of protein corona formation on fusion. **Figure** **4**A shows representative FLIM images and Figure 4B shows the fluorescence decay of the FRET donor Bodipy C_16_ for: a control GUV in the absence of liposomes, a GUV incubated with pristine liposomes, and a GUV incubated with liposomes preincubated with serum. As expected, pristine liposomes efficiently fuse with the GUVs, reducing the acceptor lifetime compared to the control GUV as a result of fusion. In sharp contrast, liposomes preincubated with serum do not undergo fusion as observed by the unmodified donor lifetime. Quantitative analysis on many individual GUVs shows that, for liposomes with comparable σ_M_, fusion is blocked if the liposomes are preincubated with serum, irrespective of the CL used (Figure 4C). Similar results are observed for the intensity analysis (Figure S12, Supporting Information) and they are not dependent on GUV size (Figure S13, Supporting Information).

The FLIM‐FRET assay above precisely detects fusion efficiency but it is insensitive to liposome binding to GUVs. To gain insights into the effect of corona on the separate processes of liposome binding and fusion, we performed multicolor microscopy of liposomes (cyan), a serum model comprised of fluorescently‐labeled albumin (green) of well‐defined concentrations, and GUVs (red). Albumin is a negatively charged protein and incubation with the cationic liposomes is expected to result in liposome‐albumin complex/corona formation. Because GUV imaging was performed in the sequential mode to minimize spectral cross‐talk, the slow imaging combined with fast diffusion makes it difficult to assign co‐localization between albumin and liposomes. Nonetheless, co‐localized green–cyan spots are observed for various albumin concentrations (Figure S14, Supporting Information), indicating protein–liposome complex formation. Protein complex binding to the GUV surface is also observed (Figure 4D). Increasing the concentration of albumin gradually decreases fusion as assessed from the reduced transfer of lipids from the liposomes to the GUVs (Figure 4D,E). While liposomes in the absence of albumin exhibit a single distribution of zeta potential that is highly positive (≈40 mV), the presence of nanomolar albumin concentrations induces the formation of liposome subpopulations with reduced zeta potentials, although still positive, as well as a subpopulation with a similar charge as the liposomes in the absence of albumin (Figure S15, Supporting Information). Hence, we suggest that insufficient protein coating enables liposome binding as there are presumably uncoated regions in the liposome surface and/or completely uncoated liposomes. Thereby, liposomes can dock and fuse with the GUVs at low albumin concentration. At intermediate concentrations, they are still able to bind to the GUVs but fusion becomes less favorable, while at higher concentrations, both docking and fusion are completely blocked. This may be due to increased surface coverage on the liposomes, shielding liposome charges and blocking docking (Figure 4F). However, given that a subpopulation of liposomes still appear to have little protein coverage at the higher albumin concentrations (Figure S15, Supporting Information), the presence of free proteins in the environment may also contribute to the reduced liposome docking efficiency. Fusion efficiency reaches its half already at ≈75 nM albumin and is completely blocked with ≈250 nM of albumin. Human serum albumin has an approximate concentration of 0.5 mM in human blood,^[^ ^54^ ^]^ which is 3 orders of magnitude higher than the highest concentration tested here. This suggests that the cationic liposomes used here would be immediately covered with a dense layer of albumin (as well as other blood proteins), and thus, unable to fuse with cells. In summary, the results show that the presence of proteins in the dispersion blocks fusion by preventing liposome binding to the GUVs and that liposome docking and fusion seem to be separate processes controlled by the degree of corona coating that ultimately shields nanoparticle charge.

### 1.6 Fusion‐Mediated Intracellular Delivery of Cargos in Living Cells and PM Permeabilization

**Figure 5:**
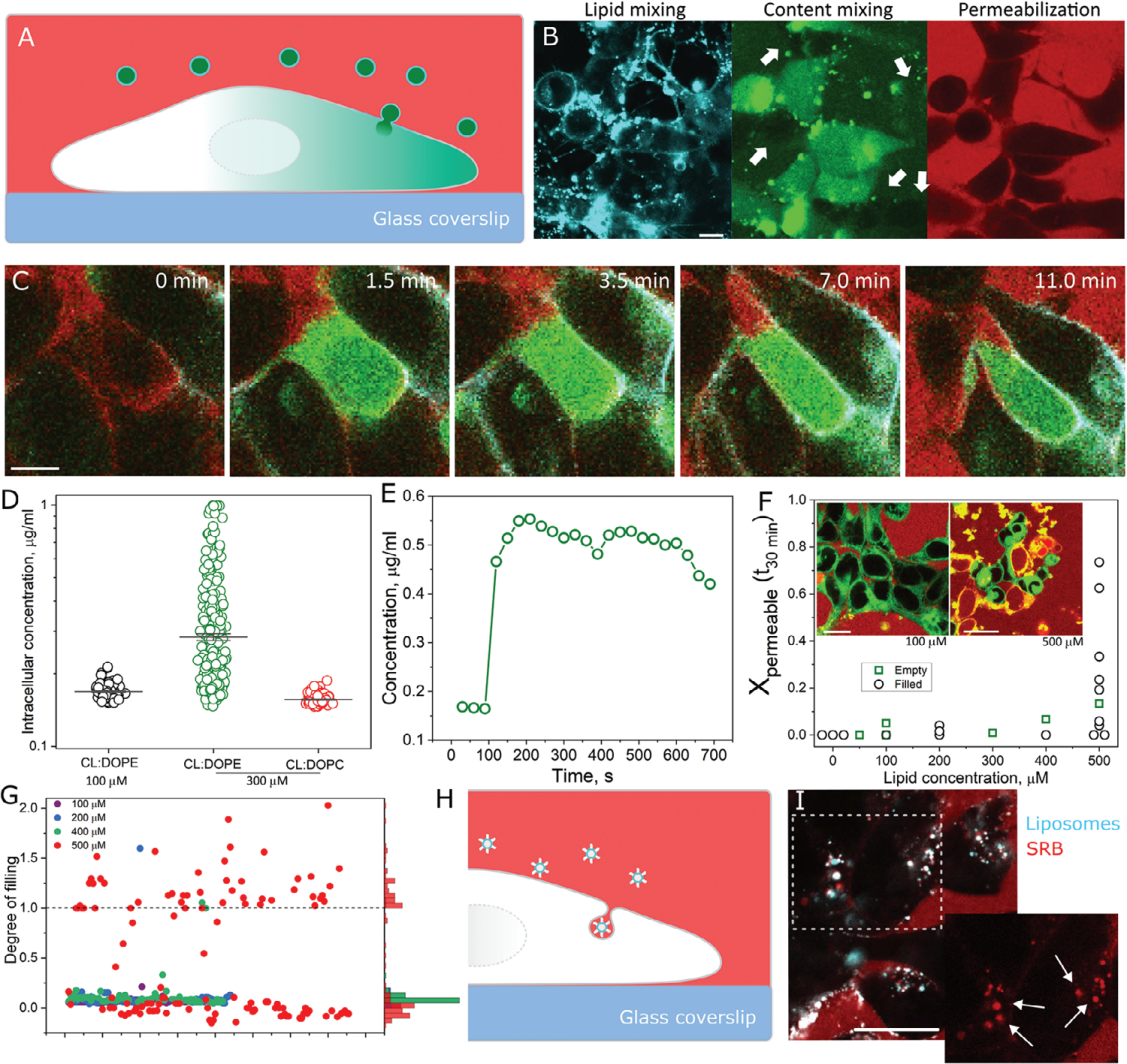
Intracellular delivery of cargos is associated with plasma membrane permeabilization only at high liposome concentration. A) Sketch of the putative delivery process. Liposomes (cyan membrane) deliver the encapsulated water‐soluble cargo (green) by direct fusion with the PM of living cells. B) Representative confocal images of DOTAP:DOPE liposomes (1:1 mole fraction) incubated with living HEK cells at 300 µM lipid concentration. Exposure results in the transfer of Atto647‐DOPE from the liposome membrane to the PM as well as the delivery of Dextran 3 kDa, green (encapsulated at a nominal concentration of 40 µg ml^−1^). Incubation was done in the presence of 10 µM SRB as a cell leakage marker. Arrows in the Dextran channel point at cells whose Dextran 3 kDa signal is below detection sensitivity (and hence not visible in this channel). C) Real‐time visualization of intracellular delivery of water‐soluble cargo. Note that due to its small size (≈1 nm) Dextran 3 kDa can locate in the nucleus. D) Intracellular Dextran 3 kDa concentration for the cell shown in panel C. The decrease in intracellular Dextran signal at later times is due to the cell moving slightly out of focus. E) Single cell intracellular Dextran 3 kDa concentration for different liposome compositions and concentrations after 30 min of incubation. Mean and standard error of the mean are shown. Note the log scale. F) Fraction of cells that become permeable to SRB as a function of liposome (DOTAP:DOPE, 1:1 mole fraction) concentration (total lipid) during 30‐min incubation. Each data point represents the measured fraction of tens of individual cells. Black and green data represent empty and Dextran 3 kDa‐filled liposomes. Inset, image of cells incubated with 100 µM or 500 µM DOTAP:DOPE (1:1 mole fraction) liposomes labeled with Bodipy C_16_. G) Measured degree of filling of individual cells for several liposomal (total lipid) concentrations. The dashed line indicates the expected values for complete permeabilization. Values above 1 are a result of nonspecific SRB affinity for intracellular structures. Histograms are also shown. H) Sketch of protein‐coated liposomes co‐internalized with SRB by cells. I) Overlay of DOTAP:DOPE:DOPC (20:50:30 mole fraction) liposomes (500 µM lipid concentration, labeled with 0.5 mol% Atto647‐DOPE, cyan) and SRB after preincubation with 10x‐diluted serum. Scale in C: 10 µm. All other scale bars: 20 µm. The inset shows the magnified image from the dashed square in panel I.

Given the rich effects of liposome fusion on model membranes, we wondered whether these effects are also mirrored in living cells, and by extension, how biophysical and biological processes are intertwined. As proof‐of‐concept, we used HEK cells, because it is one of the most commonly used cell lines and because we have previously characterized the uptake of polystyrene nanoparticles by these cells.^[^ ^55^ ^]^ We anticipate that liposome behavior will not qualitatively differ across mammalian cell types, as the fusogenic capacity of our system is based on membrane charge, though quantitatively it likely will. Prior studies have shown efficient liposome fusion to a variety of cell types, including primary cells, from both human and rat sources, as well as tissue.^[^ ^20^ ^]^ We incubated living human embryonic kidney (HEK) cells with cationic liposomes and tested their ability to fuse, deliver encapsulated water‐soluble cargos, and potential PM disruption. The experiments were performed in the absence of a protein corona, while we discuss the results for liposomes coated with a corona in the next section. Cyan‐labeled Atto647‐PE was used as lipid marker with liposomes encapsulating Dextran 3 kDa (green) as a water‐soluble cargo model, and cell incubation was carried out in the presence of SRB (red) in the outside medium to test for PM permeabilization (**Figure** **5**A). MVL5 liposomes exhibit weak PM fusion signal. Instead, they are predominantly internalized by cells, as observed by co‐internalization with SRB (Figure S16 and Movie S2, Supporting Information). For DOTAP liposomes, in contrast, efficient lipid and content mixing is observed (Figure 5B) when cells are incubated with 300 µM liposomes (lipid concentration) for 30 min, consistent with fusion. In the early stages of incubation with the liposomes, the lipid marker is preferentially located in the plasma membrane, whereas it is internalized at later stages, although the intracellular localization of the membrane markers depends on the probe used (Figure S17, Supporting Information). Importantly, cells do not become permeable with this vesicle concentration, despite the more than one order of magnitude higher concentrations used compared to those that were found to disrupt GUVs.

As shown in Figure 5B (arrowheads), there is a fraction of cells whose amount of delivered cargo is below our detection sensitivity, showing that the delivery process is heterogeneous. In order to quantify the delivery, we made an, admittedly rough, estimate using the calibrated signal of free Dextran 3 kDa (Figure S18, Supporting Information). The mean intracellular concentration of Dextran 3 kDa (when encapsulated at 40 µg ml^−1^ in DOTAP:DOPE liposomes and when the liposomes are administered at 300 µM lipid concentration) is 0.28 ± 0.17 µg ml^−1^ (mean ± s.d.), with as much as 1 µg ml^−1^ in some cells (Figure 5D). Note that due to its small size (≈1 nm diameter),^[^ ^56^ ^]^ the probe can also enter the cell nucleus. The maximum observed intracellular dextran concentration (1 µg ml^−1^) corresponds to ≈10^[^ ^5^ ^]^ individual liposomes fusing to a single cell. This estimate is based on an average HEK cell volume of 5000 µm^3^, approximating the cell as a 10–15 µm diameter hemisphere, and an average liposome diameter of 100 nm. Decreasing liposomal concentration to 100 µM or using DOPC as the helper lipid results in a much lower level of intracellular delivery and an even larger heterogeneity across the cells, although the amount of delivery material can still be high in some cells (Figure S19, Supporting Information). Of note, the intracellular delivery is a very fast process. Figure 5C and Movie S3 (Supporting Information) show the real‐time delivery of Dextran 3 kDa to an individual cell, reaching ≈0.5 µg ml^−1^ concentration for the observed cell (Figure 5E). Intracellular delivery of this significant amount of cargo happens within a few seconds, showing the high delivery speed. While it has been shown in GUV models that fusion of cationic liposomes takes place in a few seconds or faster,^[^ ^38^ ^]^ these results show that intracellular delivery of cargos are comparatively fast in living cells, corresponding to orders of magnitude faster delivery compared to internalization‐based pathways.^[^ ^55^, ^57^, ^58^, ^59^ ^]^

We next tested the susceptibility of the plasma membrane to damage by incubating HEK cells with increasing liposome concentrations and measured the fraction of cells that become permeable to SRB within 30 min. As with GUVs, disruption is assessed by PM membrane permeabilization and SRB entry. Real‐time observation is necessary since a small but detectable fraction of cells (≈5%) are intrinsically permeable to SRB, most likely corresponding to dead cells, and hence only those that became permeable during liposome treatment were considered for analysis. For intact cells in the absence of liposomes, SRB has no affinity for the PM nor does it diffuse in or is internalized by the cells during the observation period (Movie S4, Supporting Information), whereas SRB binds intracellular structures in chemically‐permeabilized cells (Figure S20, Supporting Information). Upon exposure to liposomes at a 100 µM lipid concentration, extensive mixing is detectable and the cells do not change their morphology nor do their PM become permeable to SRB (Movie S5, Supporting Information). At 400 µM, more intense mixing is detected and some cells exhibit morphological changes although with no sign of PM permeabilization (Movie S6, Supporting Information). Only at a 500 µM lipid concentration do some HEK cells become permeable to SRB (Movie S7, Supporting Information). As shown in the inset in Figure 5F, permeable or intact cells are clearly identifiable. Figure 5F shows the fraction of cells that become permeable to SRB as a function of liposome concentration. Note that although there is some variation between different experiments, significant PM permeabilization is only observed at the higher liposome concentrations. These effects are independent of whether the liposomes are empty or whether they are filled with cargo. The results represent end‐point measurements, and thus. cannot give information on the stochasticity of pore formation. Of note, in the absence of corona, PM damage only occurs upon a combination of high liposome concentration and high σ_M_, whereas 500 µM lipids but lower σ_M_ liposomes do not permeabilize cells (Figure S21, Supporting Information).

More detailed permeability analysis at the single cell level shows that PM permeabilization is an all‐or‐none mechanism (Figure 5G). The values above 1 are a result of the intrinsic SRB affinity for intracellular structures. All‐or‐none permeabilization indicates that the pore lifetime or pore size is longer/larger than that in GUVs, and the pores remained open until at least intracellular equilibration of SRB concentration. Notably, for permeable cells whose morphology is not or only mildly altered, the delivered Dextran 3 kDa cargo remains entrapped despite PM permeability to SRB (Figure S22, Supporting Information). This shows that the pores formed have a size threshold, and even the formation of small pores does not necessarily forbid the use of cationic liposomes as efficient delivery vehicles, especially if the cells are able to recover the injury once the liposomes are removed from the medium (see below). While the cell density and available membrane area per cell are very different to those in the GUV experiments, the results suggest that cells are more resilient against disruption than GUVs, although this should be interpreted carefully. In summary, liposomes enables fast delivery of large amounts of cargos, although heterogeneously, and the liposomal concentration required for intracellular cargo delivery is below that which induces PM disruption.

### 1.7 Liposome Fusion Fluidizes the PM of HEK Cells

**Figure 6:**
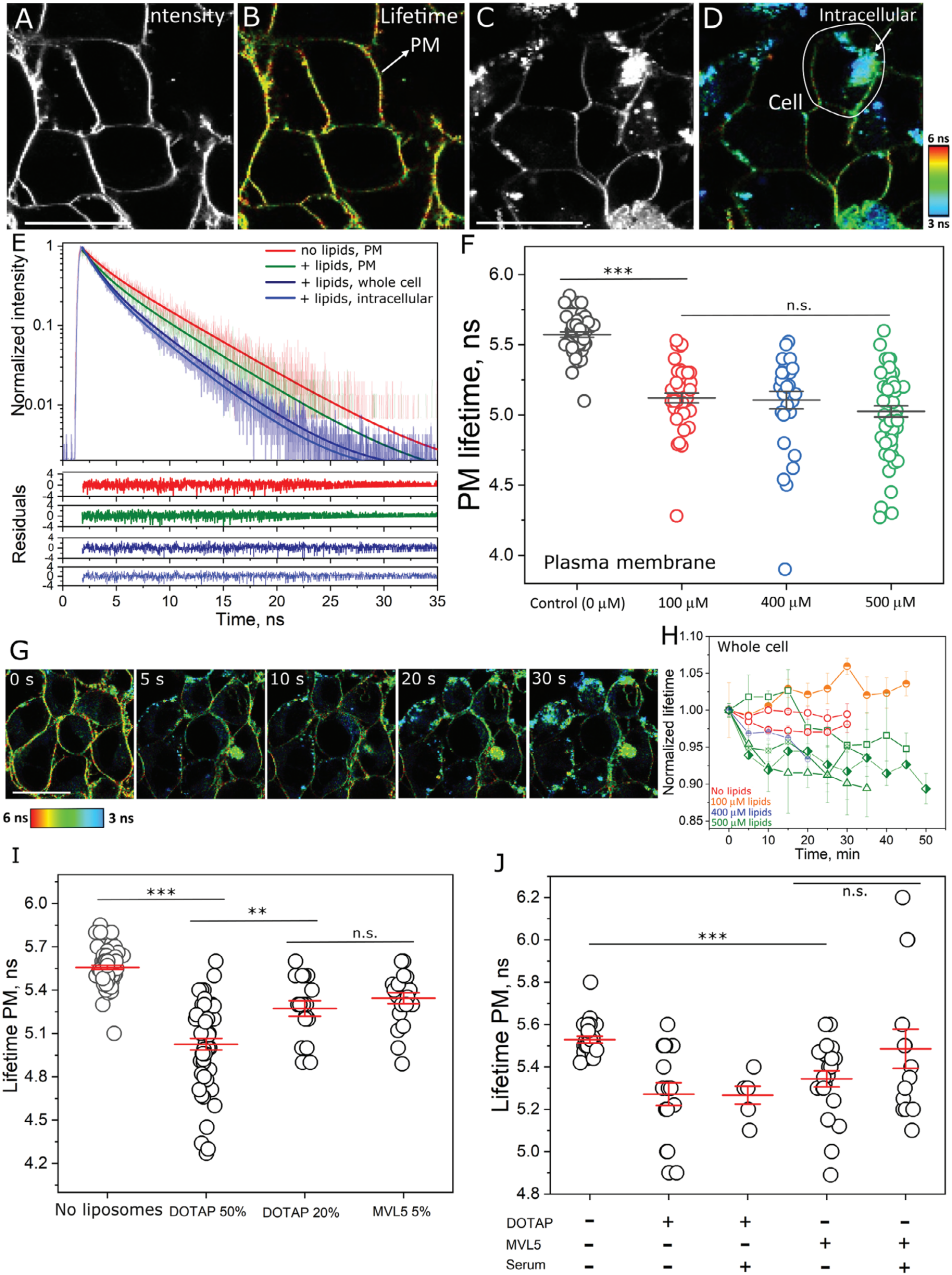
Liposome fusion fluidizes the plasma membrane. A) Cells labeled with FliptR (1 µM in the cell medium) retain the probe at the PM for extended periods (at least 30 min) as observed from fluorescence intensity. B) FLIM image of the same cells, highlighting the PM. C) Extensive bright puncta upon incubation with nonlabeled 500 µM (total lipids) DOTAP:DOPE (1:1 mole fraction) liposomes. The reduced PM signal compared to control without liposomes indicates that some of the PM has been internalized. D) FLIM image of the same group of cells shows that the FliptR's lifetime in the PM is reduced. The intracellular puncta/bound liposomes (arrow) exhibit an even shorter lifetime. E) Measured fluorescence decay along with the fits and residuals from the fit for the structures shown in (B) and (D). For all regions and systems, FliptR's lifetime is best fitted with a double exponential decay model. F) long lifetime measured on the PM for control cells (no liposomes) and cells incubated with increasing concentrations of DOTAP:DOPE (1:1 mole fraction) liposomes. Each circle represents the measurement on a single cell. Statistical significance assessed by an independent two‐sample *t*‐test at a level of 0.05. G) Time‐lapse FLIM images of a group of cells incubated with 500 µM (total lipids) DOTAP:DOPE (1:1 mole fraction) liposomes. H) Representative whole cell dynamics of lifetime changes. Errors (standard errors of the mean) indicate variations from different cells. The lines are a guide to the eye. I) Measured FliptR long lifetime at the PM for liposomes containing DOTAP or MVL5 as the CL and with different σ_M_. J) Effects of serum on the changes in PM lifetime. All scale bars: 20 µm.

From GUV model membranes, changes in fluidity are a result of the final membrane composition after fusion. Given the very ordered nature of the PM of cells,^[^ ^60^ ^]^ we thus anticipate that liposome fusion, and thus, transfer of DOTAP and DOPE lipids, is expected to lead to an increase in membrane fluidity, which using FliptR as a fluidity marker would result in a decrease in lifetime. FliptR strongly accumulates at the PM for extended periods of time in HEK cells (**Figure** **6**A,B), as for other cell lines.^[^ ^50^ ^]^ For control HEK cells in the absence of liposomes, the lifetime measured inside the cells (i.e., intracellular membranes) is lower compared to that of the PM (Figure 6D and Figures S23 and S28, Supporting Information), demonstrating the PM's high packing as previously reported.^[^ ^49^, ^50^, ^60^ ^]^ The measured lifetime at the PM is 5.55 ± 0.12 ns for at least 30 min observation (Movie S8, Supporting Information), in accordance with other studies.^[^ ^49^, ^50^ ^]^ Interestingly, incubation with nonlabeled cationic liposomes containing 20% or 50% DOTAP results in liposome labeling (due to free FliptR in the medium) and internalization, possibly along with internalization of some PM material (Figure 6C,D and Movie S9, Supporting Information). As a result of stronger intracellular signal compared to the control, the signal from whole cell measurements, otherwise dominated by the longer lifetime of the PM, is now skewed to shorter lifetimes due to the contribution from the bright intracellular structures in a liposome concentration‐dependent manner (Figure 6G,H). The decrease in whole cell lifetime is fast and occurs in less than 5 min though there are cell‐to‐cell variations.

When we measure FliptR lifetime specifically at the PM, we also observed clear PM fluidification (Figure 6F). Again, this depends on liposome concentration (Figure 6F and Figure S23, Supporting Information). The extensive incorporation of external lipid material via fusion not only decreases PM packing, but it also decreases membrane tension in GUVs^[^ ^38^, ^61^ ^]^ as well as in cells,^[^ ^62^ ^]^ and thus, it is tempting to speculate that the appearance of intracellular membrane material of lower fluidity may be comprised of internalized vesicles. Interestingly, PM fluidification occurs at liposomes concentrations lower than those necessary to induce pore formation and has, in fact, already leveled off at 100 µM lipid concentration. An average similar PM fluidification is observed at low σ_M_ using DOTAP or MVL5 liposomes, although with a reduced fraction of highly fluid membranes (Figure 6I). This not only shows that the changes in fluidity also depends on σ_M_, but also indicates that these changes occur earlier than induced PM permeabilization. We conclude that the extensive incorporation of lipids in the PM due to direct liposome fusion fluidizes the PM and likely reduces surface tension, leading to internalization of PM material, in a way that depends on liposome concentration and charge density, and that changes in fluidity are milder effects compared to PM pore formation.

### 1.8 Effects of Protein Coating on Liposome–Cell Interactions, Fluidification, and PM Permeabilization

Preincubation with serum screens the charge and, in general, interactions of cationic liposomes, most likely by completely coating their surface, as also observed for other particle models,^[^ ^44^, ^63^ ^]^ preventing fusion with GUVs. We next tested whether corona formation also prevents particle binding and fusion with living cells. Here, cells were exposed to the liposomes in the presence of both a hard and soft corona, as well as in the presence of serum proteins in the medium, comparable to the experiments presented in Figure 4. Liposome internalization and content mixing can be easily distinguished by their staining pattern; whereas content mixing leads to homogeneous labeling of the PM with the lipid marker as sketched in Figure 5A and shown in Figure 5B, internalization leads to a spotted pattern. Furthermore, as vesicles formed during the internalization process inevitably engulf some extracellular liquid content, SRB becomes co‐internalized with liposomes, and thus, internalization is detected as cyan‐red spotted pattern as sketched in Figure 5H and shown in Figure 5I. Figure S24, Movie S10, and Movie S11 (Supporting Information) show that DOTAP and MVL5 liposomes upon preincubation with serum are internalized by cells rather than directly fusing to the PM. Of note, these liposomes do not induce PM permeabilization, even at the highest concentration tested (Figure S25, Supporting Information) although they also fluidize the PM, regardless of the CL used (Figure 6J and Figure S26, Supporting Information). Nevertheless, the fraction of cells with highly fluid PM is reduced, showing that the effects of protein‐coated liposomes on membrane properties are milder. In summary, protein corona formation does not prevent liposome binding to the PM of living cells but it does change the mode of interaction, switching from content mixing to internalization. This is reflected in their much milder mechanical effects on cells.

### 1.9 Changes in Material Properties are Transient

All experiments above involve the assessment of GUVs and cells upon acute treatment with liposomes. For drug and gene delivery applications, many protocols involve a short pulse with the delivery vehicle, followed by vehicle removal to reduce potential side effects. If the vehicle induces toxic effects, as is typical for cationic particles,^[^ ^18^, ^40^, ^41^, ^44^ ^]^ particle removal may allow surviving cells to recover whilst still achieving cargo delivery. To test whether the effects observed in our system, namely pore formation and changes in membrane fluidity, are permanent or temporary, we incubated the cells with increasing concentrations of liposomes as above, removed the liposomes after 30‐min incubation, and placed the cells back under cell culture conditions for 24 h to allow cell recovery. Because we have characterized and optimized the experimental conditions for GUVs in sucrose solutions, and because preparing GUVs in high ionic strength solutions is typically more cumbersome, we opted to use sucrose for the cell experiments. The experiments were performed with isotonic sucrose solutions and for limited times to minimize side effects. On the other hand, this approach has the advantage that the conditions are more closely comparable with the GUV results. When compared to the control cells (treated with sucrose solution in the absence of liposomes), the liposomes induce a similar fraction of dead cells (≈20–40%) for all concentrations tested (Figure S27, Supporting Information). Note that the fraction of dead cells is already higher in sucrose solution itself compared to cells with cell medium alone, but sucrose (or other low ionic strength solution) was required due to liposome preparation and for better comparison with the GUV experiments. After the recovery period, the remaining cells (≈60–80%) were incubated with SRB to assess whether PM pores were still present. No intracellular signal of SRB was observed, indicating that the PM was intact, even at the liposomal concentrations that induced significant PM damage (Figure S28A,B, Supporting Information). As can be seen in Figure S28C (Supporting Information), FliptR, which was added to the cells before imaging (after the recovery period), largely stains the plasma membrane for all concentrations tested, with intracellular labeling indistinguishable from that of control conditions without liposomes. This indicates that incorporated lipids, internalized PM materials and liposomes have been fully processed. Importantly, FliptR's lifetime measured at the PM is identical in all conditions, even at concentrations well above those that induced changes in acute treatment (Figure S28D, Supporting Information). In summary, while administered liposomes are able to deliver cargos into living cells, any measured adverse effects on PM membrane integrity, changes in membrane fluidity or presence of large amounts of internalized lipid materials are recovered to steady conditions upon liposomal removal.

## 2 Discussion

**Figure 7:**
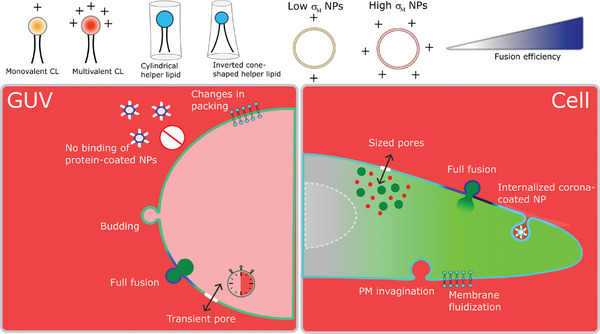
A summary of fusogenic liposome nanoparticle interaction with model membranes and cells. *Top left*: Monovalent (orange headgroup) or multivalent (red headgroup) cationic lipids (CL) and cylindrical or inverted cone‐shape helper lipids. *Top right*: Liposome membrane charge density can be tuned by increasing the mole fraction of CL. Bottom panels: main observations of changes in the material properties of GUV membrane models and living cells. *Bottom left*: In GUVs, fusion occurs via i) content mixing, leads to ii) changes in membrane fluidity/packing, which can iii) increase/decrease depending on the initial and final membrane compositions, iv) leads to bud formation due to changes in spontaneous curvature, and v) the opening of transient hydrophilic pores. Heavily corona‐coated liposomes are unable to bind. The results are independent of the chemical composition of the CL or helper lipid. *Bottom right*: In cells, cationic liposomes directly fuse with the PM, leading to a fast and efficient delivery of cargos in the cytosol. Fusion fluidizes the membrane as external low *T*_m_ lipids are incorporated in the highly packed PM. Extensive incorporation of lipids likely leads to active internalization of PM content to counterbalance a decreased tension. Fusion also leads to the formation of pores of defined sizes that allow the entry of small probes from the medium but the delivered large cargos are retained. Cationic liposomes heavily coated with a protein corona are not able to directly fuse with the PM but efficiently bind to the membrane and are internalized.

In this work, we show that cationic fusogenic liposome nanoparticles efficiently deliver encapsulated cargos to GUV model membranes and living cells, though they modify a number of fundamental material properties of the target membranes in the process. **Figure** **7** summarizes these observations. Fusion increases membrane area upon incorporation of new lipids, initially decreasing membrane tension, as observed in GUVs by the appearance of membrane fluctuations and increase in volume, and as indicated in cells by the internalization of PM material. Depending on the initial membrane composition, the newly added lipids may increase or decrease membrane packing and fluidity, which tend towards values intermediate between those of the acceptor membrane and liposomes. In cells, fusion fluidizes the PM. Due to their small sizes, liposomes carry an intrinsic leaflet asymmetry, with more lipids in their outer than inner leaflet.^[^ ^64^ ^]^ Fusion of a large enough number of liposomes brings this asymmetry to the acceptor membranes, leading to budding as well as an increase in curvature tension that overcomes the initial decrease in tension from the gained area. In extreme conditions, curvature tension build‐up results in the formation of membrane pores of specific sizes, and they may retain the delivered cargo depending on cargo size. Interestingly, the formed pores are transient and they close in a few minutes in GUVs. For corona‐coated liposomes, charge shielding prevents strong electrostatic association with the acceptor membranes, hindering binding and fusion with GUVs, and shifting the mechanisms of interactions with cells from direct fusion to internalization. While these modifications do not necessarily mean serious adverse effects, because under certain conditions the cells are able to fully recover to preliposome treatment state, they are worth considering when designing a drug delivery vehicle. To the best of our knowledge, drug delivery‐mediated changes in the material properties of synthetic and biological membranes were until now unknown.

### 2.1 Effects of Charge Density and Helper Lipid

Cationic nanoparticles, including liposomes, are known to bind more readily to cell and model membranes compared to neutral or anionic nanoparticles^[^ ^65^, ^66^ ^]^ and are also reportedly inducers of membrane pores.^[^ ^67^ ^]^ We show that membrane charge is crucial for membrane interaction, as neutral liposomes fail to bind to GUV membranes; we expect similar behavior from negative liposomes. In the specific case of lipoplexes, helper lipids improves gene delivery,^[^ ^68^ ^]^ but the role of helper lipids is less known in other contexts.

In many ways, intracellular delivery of encapsulated water‐soluble cargos via direct PM fusion mirrors the activity of cationic liposomes used in gene delivery. For lipoplex nanovehicles, high σ_M_ leads to efficient gene delivery independent of the helper lipid, although the helper lipid determines the interaction mechanism; from complex internalization with cylindrical lipids to direct fusion with the PM with inverted cone‐shaped helper lipids.^[^ ^32^, ^43^ ^]^ Similarly to transfection of foreign genes in cells,^[^ ^43^, ^69^, ^70^ ^]^ or the interaction of other types of charged nanoparticles,^[^ ^71^ ^]^ binding and fusion of cationic liposome nanovehicles with acceptor synthetic and biological membranes depends on σ_M_. At high σ_M_, liposome binding is almost instantly followed by fusion, and the helper lipids are dispensable, whereas at intermediate or low σ_M_, membrane–membrane interaction is weakened, fusion is delayed, and inverted cone‐shaped lipids enhance fusion. Similarly, bound weakly charged nanoparticles may eventually diffuse away upon binding.^[^ ^71^ ^]^ Delayed fusion gives the cells the ability to internalize the bound particles as we have recently shown.^[^ ^55^ ^]^ In contrast, noncharged liposomes or liposomes whose charges have been completely screened by a protein corona coat are unable to bind to model membranes although they do bind to cells. In this case, binding results in internalization rather than direct fusion.

In addition to charge, the helper lipid plays an important role in nanoparticle–membrane interactions. Although we focused on two of the most commonly used helper lipids, namely DOPC and DOPE, other helper lipids could have been tested, including cholesterol, DPPC, or more complex lipids such as PEGylated lipids. Solid cationic nanoparticles containing Chol in either homogeneous membranes or in defect regions of membranes exhibiting membrane phase separation increase binding and lipid exchange (the equivalent of fusion in our system) to acceptor anionic membranes.^[^ ^71^ ^]^ For cationic fusogenic liposomes, Chol does not seem to increase binding, although it does decrease fusion due to an increase in membrane stiffness,^[^ ^36^ ^]^ and does modify the interaction energetics.^[^ ^72^ ^]^ DPPC is a long and fully saturated lipid with a high melting temperature (*T*_m_), and thus, forms gel membranes. If mixed with lipids of low *T*_m_, such as those used here, DPPC induces liquid–solid phase separation, which we have shown to aid fusion due to the presence of membrane defects.^[^ ^73^ ^]^ High enough σ_M_ bypasses the need of these factors. Lastly, PEGylated lipids exhibit inhibitory effects on lipoplex interactions,^[^ ^70^ ^]^ and recent observations have shown that PEG chains screen membrane charge resulting in inhibitory effects on fusion depending on polymer size and surface coverage (Lira et al., in preparation). Hence, despite the extremely diverse nanoparticle types and delivery mechanisms, the effects of charge density in drug delivery vehicles seem to be more general than might have been anticipated.

### 2.2 Nanoparticle Toxicity

Cationic lipids do not exist in nature and the administration of cationic particles, including liposomes, has been shown to induce toxicity via various mechanisms.^[^ ^74^, ^75^, ^76^ ^]^ One of such proposed mechanisms includes the need for the cell to metabolize these foreign species.^[^ ^43^, ^77^ ^]^ The use of multivalent lipids could potentially circumvent nanoparticle toxicity by achieving high σ_M_ at low CL fractions, reducing metabolic demands on the cell.^[^ ^77^, ^78^ ^]^ However, as we showed for both synthetic and biological membranes, for comparable amounts of administered nanoparticles, σ_M_ also determines toxicity (as assessed from PM permeabilization) irrespective of the chemical composition of the CL. To reduce toxicity, one strategy could include nanoparticle coating with inert proteins or polymers, such as PEG. PEGylation has the added advantage of improving the stability of positively charged lipid‐based carriers in complex biological media such as the blood. However, PEGylation may inhibit interactions with the cell membrane, resulting in reduced uptake, and in particular, interfere with the fusogenic capacity of the carrier. However, coating not only reduces fusion efficiency at moderate surface coverages, creating a charge/coating dilemma observed with stealth liposomes,^[^ ^8^, ^79^ ^]^ but also shifts the interaction to particle internalization. A more reasonable alternative may constitute the use of cleavable components as shown for PEG‐coated liposomes,^[^ ^70^ ^]^ in which on‐demand removal of the coating would expose nanoparticle charge, and hence induce fusion, although this remains to be demonstrated. Importantly, membrane leakage occurs at liposomal concentrations that are higher than that required to achieve fusion and cargo delivery, and thus, for a given σ_M_, there is a relatively large range of liposome concentrations in which intracellular delivery is efficient and leakage is minimum, although the PM does fluidize. These disruption effects mirror the ability of RNA delivery to cells mediated by lipoplex nanoparticles, with gene silencing below the onset of cytotoxicity.^[^ ^69^ ^]^ Furthermore, the formed pores have a size threshold, and larger delivered cargos (here above ≈ 1 nm, the expected hydrodynamic radius of Dextran 3 kDa)^[^ ^56^ ^]^ are retained in the cell even if the PM is permeabilized. Lastly and most importantly, upon liposomal removal, the cells are able to fully recover to their state before contact with liposomes.

Taking a very simplified interpretation of the observed results by treating them as purely physical in nature, they can be understood by the mechanical effects of fusion of a large number of liposomes to acceptor membranes. Membrane pore dynamics are controlled by a balance between surface tension and edge tension, favoring pore opening and closure, respectively.^[^ ^80^ ^]^ In typical phospholipid bilayer membrane models, edge tension is high, and mechanically opened pores spontaneously close,^[^ ^81^, ^82^ ^]^ but a decrease in edge tension favors the open pore state.^[^ ^46^, ^83^ ^]^ In stably open pores, SRB leaks in to full equilibration in ≈5 min.^[^ ^46^ ^]^ As in the experiments here leakage was incomplete, we assume that the pores close in the first minute after pore opening, and thus, fusion‐dependent decrease in edge tension is significant, but not strong enough to lead to GUV collapse. The discussion above is nonetheless valid for symmetrical artificial membranes. Due to the intrinsic leaflet number asymmetry in small liposomes, their membranes have more lipids in their outer than their inner leaflets. Fusion of large enough numbers of liposomes is thus expected to also result in asymmetry in the GUVs, consequently inducing GUV bud formation outward as observed. Asymmetrical distribution of lipids across the leaflets reduces the edge tension,^[^ ^84^ ^]^ favoring the state of longer‐lived pores. A recent study showed that the number of lipids across the PM leaflets in living cells is also asymmetrically distributed, which poses consequences for PM mechanical stability.^[^ ^85^ ^]^ We are not aware of edge tension values in cells, but extensive liposome fusion may also introduce or even increase such asymmetry. It is thus tempting to speculate that, similarly to GUVs, membrane asymmetry and a decrease in edge tension may also be the reason why pores are formed in cells upon liposome fusion. If such effects take place more quickly than the ability of cells to cope with the introduced stress, pores will likely open in the PM, as indeed observed. On the other hand, if the cells survive treatment, removal of liposomes ceases stress build‐up and cellular activity may ensue to counterbalance the effects of newly introduced foreign lipids, recovering membrane fluidity, and activate membrane flippases and scramblases, relaxing leaflet imbalances. Lastly, the ability of cationic liposomes to induce membrane pores seems to depend on the number of fused liposomes. Thus, the same mechanisms would likely apply and extended incubation times or higher concentrations of liposomes with lower charge density may thus achieve similar membrane perforation levels if comparable fusion levels with acceptor membranes are reached.

### 2.3 GUVs as Cellular Membrane Models

To what extent can GUVs or other model membranes be a predictor of the behavior of biological membranes and cells? GUV membrane models, while not bearing all cellular components, can reconstitute the fundamental processes at the molecular level with incredibly high robustness. A great advantage includes the easy tuning of their material properties by the appropriate choice of their components. In the specific case of cationic liposomes, particle binding, fusion and cargo delivery, pore formation, and changes in overall material properties, such as fluidity and curvature, that were observed in GUVs were mirrored in live cell experiments. However, despite being an excellent tool, GUVs do not always predict all cellular outcomes, and even the (nearly) identical outcomes occur at vastly different liposome concentrations. On the one hand, membrane fluidity changes can be directly inferred from the changes in lipid composition, qualitatively observed in both GUVs and cells. On the other hand, major differences observed between GUVs and cells included the interaction of protein‐coated liposomes with cells, leading to nanoparticle internalization by cells, whereas they were completely unable to bind GUVs. The switch to cellular internalization mechanisms for protein‐coated liposomes likely stems from the specific binding of biomolecular corona‐proteins to cell membrane receptors, which consequently triggers their endocytosis. Indeed, the internalization pathway of several liposomal formulations, such as Doxil, has been shown to be mediated by the presence of particular proteins in the corona.^[^ ^86^, ^87^, ^88^ ^]^ Furthermore, whereas liposome fusion induces spontaneous curvature on GUVs, resulting in outward budding formation, in cells, intracellular vesicles and PM internalization were observed. While in GUVs, budding is a purely physical process that is associated with the asymmetric incorporation of lipids from the intrinsically asymmetric liposomes, resulting in more lipids in the outer than the inner leaflets,^[^ ^38^ ^]^ in cells, PM internalization is most likely actively driven. The sudden incorporation of excess of lipids via fusion acutely decreases PM tension activating the endocytosis machinery for lipid recycling.^[^ ^62^, ^89^, ^90^ ^]^ Furthermore, the high compositional complexity and interaction network of the PM with other cellular factors (i.e., cytoskeleton,^[^ ^91^, ^92^, ^93^ ^]^ media with varying ionic strengths,^[^ ^46^, ^94^ ^]^ PM asymmetry,^[^ ^34^, ^85^ ^]^ and the presence of protein machinery responsible for resealing pore damage)^[^ ^95^ ^]^ likely reflects the higher stability of cells compared to free‐standing GUV membranes.

### 2.4 Direct Membrane Fusion Versus Internalization‐Based Pathways

Most nanoparticle‐based drug delivery strategies are, one way or another, based on particle internalization by cells, with a few exceptions.^[^ ^96^, ^97^ ^]^ This means that, at least transiently, the particles are entrapped in intravesicular compartments, such as endosomes and lysosomes. However, internalization alone is not sufficient for delivery since the vehicle–cargo complexes are still unavailable. In fact, corona‐coated liposomes that were internalized by cells were unable to release the encapsulated cargo during the observation time of our experiments, for any CL or helper lipid used and at any concentration. Hence, the cargo, either when complexed with its vehicle or (preferably) alone needs to be released from these compartments. In general, vesicular processing is slow and can take several hours.^[^ ^98^ ^]^ Long processing times in low pH and in the presence of proteases and nucleases increase the chance of cargo degradation. Thus, even in the case of successful but delayed release, the cargo might no longer be functional. Strategies to improve endosomal escape efficiency and speed includes osmotic endosomal inflation using internalized charged polymers that induce ion and water pumping into the vesicles via the proton‐sponge effect.^[^ ^99^, ^100^ ^]^ This nevertheless not only results in cytosolic release of proteases, but the number of ions needed to burst endosomes has been suggested to increase quadratically with endosomal radius, limiting escape to small endosomes.^[^ ^101^ ^]^ As shown in cells, although siRNA in complex with cationic liposomes can be efficiently internalized, release from endosomes through endosomal pores is transient and incomplete.^[^ ^102^ ^]^ In fact, endosomal escape is a more limiting factor in drug/gene availability than the degree of internalization,^[^ ^101^ ^]^ with as little as 1–2% of internalized cargo being effectively released from the endosomes.^[^ ^103^ ^]^ Due to their presumably high (but unknown) edge tension, formed pores in the endosomal membranes are likely short lived, limiting escape to small cargos and only while the pore is open, probably explaining the extremely low escape and incompleteness of cargo release from internalized vesicles.

The highly fusogenic liposomes undergo fusion with the first contacting membrane (i.e., the PM and the contacting membrane of the GUVs) within a few seconds, faster than most internalization‐based pathways,^[^ ^58^ ^]^ with direct and immediate delivery of large amounts of cargos in the cell cytosol. This bypasses slow and inefficient internalization pathways, with the additional advantage that neither the vehicle nor the cargo is exposed to the low pH and enzymatic milieu of intracellular vesicle compartments. Direct membrane fusion also circumvents the need for complex surface functionalization and intracellular processing, such as the use of pH‐sensitive moieties^[^ ^104^, ^105^ ^]^ or polymers that aid endosomal escape,^[^ ^106^, ^107^ ^]^ significantly simplifying formulation design. Direct fusion‐based intracellular delivery is very likely extendable to any water‐soluble cargo and amenable to cargo co‐delivery,^[^ ^108^ ^]^ and it is not limited by cargo size or specific chemical identity, as successfully demonstrated for proteins,^[^ ^109^, ^110^ ^]^ oligonucleotides,^[^ ^111^ ^]^ and nanoparticles.^[^ ^110^, ^112^ ^]^ The many advantages of direct fusion‐based cargo delivery using fusogenic liposomes make them extremely attractive nanovehicles in drug delivery. The discovery that the extensive fusion of a large number of liposomes transiently modifies the material properties of acceptor membranes and cells in their course of cargo delivery will help the development of more efficient delivery systems with limited and controlled side effects.

## 3 Conclusions

Drug delivery nanoparticles are excellent tools to increase the availability of cargos at sufficient amounts in the target location, ideally quickly, while being inert; that is, they should not induce cellular responses or alter the cell state upon cargo delivery. Here, we show that highly charged cationic liposomes fuse with synthetic vesicles and the biological PM of living cells to efficiently deliver large amounts of encapsulated cargos within seconds, bypassing slow and often inefficient internalization‐based pathways. However, in the course of cargo delivery, they modify several material properties of the acceptor membranes, including membrane packing, spontaneous curvature and surface tension and they lead to the formation of transient pores of defined sizes. To the best of our knowledge, this is the first study to systematically assess the effects of a drug delivery vehicle on the mechanical properties of membranes. We show that for cationic liposomes, the molecular mechanisms of toxicity are associated with a combination of local changes in composition, curvature and tension. Importantly, there is a relatively wide concentration range that achieves effective intracellular delivery, and although some cell toxicity is observed, it is reversed by the removal of liposomes after successful delivery. These effects depend on liposome charge density and can be further modulated by the type of helper lipid. Due to their high charge, intracellular delivery is limited to ex vivo applications since in the body serum proteins would quickly bind and cover their surface. However, they could be fairly adaptable to ex vivo applications with efficient and fast administration in serum‐free media and with limited side effects once the cells return to physiological conditions. In many aspects, fusion‐based drug delivery mirrors lipoplex systems despite major differences in structures and interactions with the target membrane, indicating a more universal role of charge than anticipated. We expect the results to aid the design of more efficient drug delivery systems with limited toxicity.

## 4 Experimental Section

All chemicals and materials were used as obtained. The phospholipids 1,2‐dioleoyl‐sn‐glycero‐3‐phosphocholine (DOPC), 1,2‐dioleoyl‐sn‐glycero‐3‐phospho‐(1′‐rac‐glycerol) (sodium salt) (DOPG), 1,2‐dioleoyl‐sn‐glycero‐3‐phosphoethanolamine (DOPE), l‐α‐phosphatidylserine (Brain, Porcine) (sodium salt) (PS), 1,2‐dioleoyl‐3‐trimethylammonium‐propane (DOTAP), N1‐[2‐((1S)‐1‐[(3‐aminopropyl)amino]‐4‐[di(3‐amino‐propyl)amino]butylcarboxamido)ethyl]‐3,4‐di[oleyloxy]‐benzamide (MVL5) and cholesterol (plant derived), as well as the fluorescent dye 1,2‐dipalmitoyl‐sn‐glycero‐3‐phosphoethanolamine‐*N*‐(lissamine rhodamine B sulfonyl) (ammonium salt) (DPPE‐Rh) were purchased from Avanti Polar Lipids (Alabaster, AL). DOPE‐Atto647N was purchased from AttoTech (Siegen, Germany). Lipid solutions were prepared in chloroform and stored at –20 °C until use. Glucose, sucrose, Dimethyl sulfoxide (DMSO) and the fluorescent probes Bodipy C_16_ (BODIPY FL C16; 4,4‐Difluoro‐5,7‐Dimethyl‐4‐Bora‐3a,4a‐Diaza‐s‐Indacene‐3‐Hexadecanoic Acid), sulforhodamine B (SRB), Dextran‐FITC 3 kDa, albumin–fluorescein isothiocyanate conjugate (FITC‐albumin) were purchased from Sigma‐Aldrich (St. Louis, MO, USA). Bovine serum albumin (BSA) and Poly(vinyl alcohol), PVA 87–90% hydrolyzed were also obtained from Sigma‐Aldrich. The molecular sensor FliptR plasma membrane (commercially known as Flipper‐TR) was purchased from Cytoskeleton Inc. (Denver, CO, USA).

### Vesicle Preparation

GUVs were prepared using the PVA method^[^ ^113^ ^]^ with minor modifications.^[^ ^114^ ^]^ Approximately 100 µL of a 2% (weight/volume) PVA solution (in water) was spread on two glass coverslips and the water was left to evaporate by placing the coverslip on a hot plate at ≈60 °C (typically 10 min) to form a PVA film. Next, a ≈10 µL lipid solution (2 mM in chloroform) containing the desired lipid mixture was spread on the PVA film. Chloroform was evaporated under a stream of Argon. The coverslips were sandwiched using a Teflon spacer forming a ≈2 mL chamber sealed with the help of clips. The chamber was filled with a 200 mM sucrose solution (unless stated otherwise) for ≈30 min to allow GUV growth. Unless stated otherwise, growth was performed at room temperature (R.T. = 18 ± 1 °C). Hydration was carried out in the dark for lipid mixtures containing fluorescent lipids. After hydration, the GUV solution was harvested by gentle pipetting and the GUVs were used within 1 day. For imaging, the GUVs were diluted in an isotonic glucose solution to help sediment the vesicles to the bottom of the imaging chamber. If not mentioned otherwise, the GUVs were labeled with 0.5 mol% fluorescent labels, the composition of which depended on the experiment.

Liposomes made of CL:DOPE:DOPC (X:50:50‐X) or CL:DOPC (X:100‐X), mole fraction, in which CL is the cationic lipid DOTAP or MVL5 were prepared using the hydration‐sonication method.^[^ ^112^ ^]^ For the FRET experiments, the liposomes were labeled with 2 mol% DPPE‐Rh. For the multicolor confocal experiments, the liposomes were labeled with 0.5 mol% DOPE‐Atto647N. In short, the appropriate lipid mixture (in chloroform) was added to the bottom of a glass chamber and evaporated under a stream of Argon and further evaporated under vacuum for 1–2 h to remove any trace of chloroform. Afterwards, the lipid films were hydrated with a 200 mM sucrose solution and vigorously vortexed until full lipid film detachment forming multilamellar vesicles (MVLs). For the encapsulation of water‐soluble probes, the reporter probe was prediluted in the hydrating sucrose solution. SRB was added at 50 µM and Dextran‐FITC was added at 0.1 mg ml^−1^ hydrating concentrations. Both probes were encapsulated in relatively concentrated liposome preparations (2 mM lipid) and diluted ≈100 times, thus minimizing background signal. If not stated otherwise, the final lipid concentration was 2 mM. The MVLs were sonicated using a bath sonication for ≈20 min and used within 2–3 days. GUV incubation with the liposomes was done by diluting the liposomes in sucrose to a 100 µM lipid concentration. An equivalent amount of the prediluted liposomes solution was mixed with 50 µL GUVs in glucose at the desired final liposomes concentration (typically 0–30 µM) for a final 100 µL solution. GUVs and liposomes were incubated for 10–15 min in an Eppendorf tube, after which the solution was ready for imaging. In the serum experiments for both GUVs and cells, the desired liposome concentration was preincubated with 10× diluted serum (or with the desired concentration of FITC‐albumin in the GUV experiments) in sucrose (200 and 300 mM for the GUV or cell experiments, respectively) for 10 min, before incubation with the GUVs or cells. For imaging, the incubated samples were moved to a BSA‐coated glass (1 wt% BSA) coverslip.

### Zeta Potential Measurements

The zeta potential measurements of the liposomal dispersions in the presence of different concentrations of FITC‐albumin were characterized using a Malvern ZetaSizer Nano ZS (Malvern Instruments) and ZetaSizer Software version 7.13 (Malvern Instruments). Dispersions were prepared as above. Measurements were performed in triplicate with minimally 10 runs each at room temperature. The reported results are the mean and standard deviation across repeats.

### Cell Handling, Labeling, and Incubation

Human embryonic kidney (HEK) cells (American‐type culture collection, no. CRL‐1573, lot no. 63 966 486) were used for the cell experiments. The following materials were used for cell culture and experiments: Dulbecco's minimal essential medium (DMEM, Giboc), fetal bovine serum (Gibco), phosphate buffered saline (Gibco), trypsin (0.05%, Gibco), trypan blue (0.4%, Sigma‐Aldrich), µ‐slide 8 well chambers (Ibidi), 35 mm petri dishes (Grenier Bio‐One), and a Neubauer hemacytometer (Brand). HEK cells were cultured in DMEM supplemented with 10% serum (complete medium) at 37 °C under 5% CO_2_ and a humidified atmosphere. All results are from cell cultures that tested negative for mycoplasma. Cells were seeded into µ‐slide 8 well dishes one day prior to experiments. For multicolor confocal imaging, the cells were moved to the microscope stage at 37 °C and the cells were washed once with a sucrose 300 mM solution (isotonic to culture media). In the control permeability experiment (no liposomes), the culture medium contained 10 µM SRB. For incubation with liposomes, a liposome solution (containing 10 µM SRB) in sucrose 300 mM contained the desired final lipid concentration and prewarmed at 37 °C, was added to the cells and imaging started immediately. As a positive control for PM permeabilization, 70% ethanol was applied to cells for 10 min. The ethanol was then removed and replaced with SRB solution. For FLIM experiments, the cells were prelabeled for 10‐15 min with FliptR by adding a small volume of a concentrated FliptR solution (in DMSO) to the cell culture medium to a final 1 µM final concentration, followed by 1–2 washes in 300 mM sucrose to remove the excess of probe as well as the culture medium. Afterwards, incubation with liposomes was performed in an identical manner as above.

### Cell Counting and TB Staining

For the recovery experiments upon incubation with liposomes, cells were seeded into µ‐slide 8 well dishes or petri dishes one day prior to the experiments. Cells were washed with warmed sucrose solution (300 mM) and then exposed to liposomes following the procedure above and then returned to cell culture conditions. After 30 min exposure, the liposomal dispersion was removed and the cells were washed with warmed sucrose solution followed by complete medium to remove any unbound particles. The cells were then returned to cell culture conditions with fresh complete medium for 24 h. The cells seeded in µ‐slide 8 well dishes were then used for imaging and stained using the procedure above. To determine cytotoxicity, the cells in petri dishes were removed from the incubator after 24 h, washed with phosphate buffered saline and then trypsinized to detach them from the cell surface. The cell suspension was diluted with complete medium from which 10 µl of cell suspension was mixed with equal amounts of trypan blue and loaded into one half of a Neubauer hemacytometer. Cells were manually counted separately for four of the grid quadrants. Cells without trypan blue staining were considered to be live, whereas dead cells exhibited staining. The measurements were performed in triplicate for each liposomal concentration investigated. The reported values are the mean and standard deviation across all replicates (i.e., 4 quadrants per triplicate so across 12 measurements in total).

### Microscopy Imaging

Multicolor confocal microscopy imaging was performed on a Zeiss LSM 710 scanning confocal microscope. A water‐immersion C‐Apochromat 40X/1.20 W Korr M27 objective was used for imaging. The spatial and temporal resolutions used were adjusted according to the sample conditions, but in general a consistent imaging size of 212.55 µm × 212.55 µm (1024 × 1024 pixels) was used in a frame scanning mode in a singular direction, with a pixel size of 0.21 µm. Line averaging = 4 and bit depth = 8. The green dyes Bodipy C_16_, FITC‐Albumin and Dextran‐FITC 3 kDa were excited using a 488 nm argon laser, and emission was detected between 495 and 555 nm. The orange dyes DPPE‐Rh and SRB were excited with a 543 nm HeNe laser line and emission was detected in the range between 555 and 600 nm. The far‐red dye Atto‐647N was excited with a 633 nm HeNe laser line and its emission was detected in the range between 640 and 800 nm. Images were scanned in the sequential mode to minimize crosstalk between channels. Dextran 3 kDa concentration in the cells was calculated from a calibration curve. Known concentrations of Dextran 3 kDa were prepared in sucrose and their intensities were measured, exhibiting a linear response. Considering the probe intensity does not change in the cellular milieu, cells were imaged with identical imaging conditions and the intracellular concentration was directly obtained from the calibration.

The fluorescence lifetime imaging microscopy (FLIM) experiments were carried out on an inverted microscope (Olympus IX73) equipped with time correlated single photon counting (TCSPC) (PicoQuant). The samples were imaged through a 100× (1.4 NA) oil immersion objective (UPLSAPO, Olympus). Bodipy C_16_ was excited using a 481 nm laser and its emission was collected using a 525/50 nm band pass filter. The images were acquired using the SymPhoTime 64 software and all samples were excited with a pulsed 20 MHz repetition rate. Unless stated otherwise, the samples were imaged with a 128 × 128 pixels, 1 ms dwell‐time and ≈300 µm/pixels with typical acquisition times of ≈27 s. For analysis, the GUV membrane signal at the equator or a defined region of interest (ROI) in the cell were manually selected for analysis and all pixels binned for fitting. Bodipy C_16_ fluorescence decays were fitted using an n‐exponential tail fit with a single‐ (in the absence of an acceptor) or bi‐exponential decay model (in the presence of the DPPE‐Rh FRET acceptor):

(2) $$I\left(t\right)=I_{0}\left(A_{1}e^{-t/\tau_{1}}+A_{2}e^{-t/\tau_{2}}\right)$$

where 𝐼(𝑡) is the intensity at time 𝑡 and *I*_0_ is the intensity at 𝑡 = 0. 𝐴_1_ and 𝐴_2_ are pre‐exponentials factors associated with lifetime components τ_1_ and τ_2_, respectively.^[^ ^37^ ^]^

The amplitude‐weighted mean fluorescence lifetime in the presence of the FRET acceptor (τ) could be calculated as:

(3) $$\tau =A_{1}\tau_{1}+A_{2}\tau_{2}$$

To measure the FRET efficiency (*E*_FRET)_, τ was obtained from GUVs both before (τ_𝑏𝑒𝑓𝑜𝑟𝑒_) and after (τ_𝑎𝑓𝑡𝑒𝑟_) incubation with the liposomes, in which FLIM‐FRET efficiency is given as:

(4) $$E_{\mathrm{FRET}}=1-\tau_{\mathrm{after}}/\tau_{\mathrm{before}}$$

In the case of cells labeled with FliptR, a bi‐exponential decay model was used from which the long lifetime τ_2_ was used for analysis. For the temperature‐controlled experiments at the FLIM microscope setup, a PeCon GmbH TempController 2000‐1 temperature‐controller was connected to a Heating Insert P Lab‐Tek S heating stage chamber, into which the observation chamber was placed. T‐controlled experiments at the multicolor confocal microscope setup were performed in an in‐built temperature‐control box and controlled using the microscope software.

### Viscosity Calculation from Lipid Diffusion

The viscosity of the GUV membranes (*η*_m_) was calculated from reported diffusion coefficient (*D*) data for the experimental systems used here,^[^ ^38^ ^]^ using the Saffman–Delbrück model:^[^ ^47^ ^]^

(5) $$\eta_{m}=\frac{k_{B}T}{4\pi Dh}\left[\ln\left(\frac{2L_{sd}}{a}\right)-\gamma \right]$$

where *k*_B_ is Boltzmann's constant, *T* is the absolute temperature, h represents membrane thickness (3.98 nm, as measured for POPC at 20 °C),^[^ ^115^ ^]^ *a* is the radius of the diffusing particle (assumed here as 0.5 nm) and γ ≈ 0.577, and *L*_sd_ is the Saffman–Delbrück diffusion length:

(6) $$L_{sd}=\frac{h\eta_{m}}{2\eta_{s}}$$

in which *η*_s_ is the viscosity of the surrounding fluid (1.133 cP).^[^ ^46^ ^]^ Although the model was best suited for relatively large particles embedded in or attached to the membranes (i.e., proteins) than for molecules of similar dimensions to the membrane lipid constituents (e.g., small fluorescent probes),^[^ ^116^ ^]^ it was still useful and widely used to gain information about membrane structure and properties.^[^ ^117^ ^]^ Hence, regardless of the exact obtained numerical values from the estimates of membrane viscosity, the trend would be preserved.

### Statistical Analysis

No preprocessing of data was conducted prior to statistical testing. For the GUV experiments, a minimum of 10–15 (both often much more) individual GUVs from 1 to 2 independent experiments (*E*_FRET_, membrane permeabilization, corona‐dependent membrane fusion) were performed, whereas for cell experiments, a minimum of 15 independent cells from 2 to 3 independent experiments (intracellular delivery, PM permeabilization and fluorescence lifetime) were performed. The specific number of GUVs or cells analyzed per experiment is given in the respective figure captions. Images were processed using Fiji (NIH, USA) and statistics was performed using Origin 2023 (OriginLab, Northampton, USA). The data are reported as individual measurements along with their means and either standard deviation (s.d.) or standard error of the mean (s.e.m), as clarified in the respective figure caption. To compare the different experimental conditions, a two‐sample *t*‐test was used with a significance level (*p* value) <0.05.

## Conflict of Interest

The authors declare no conflict of interest.

## Author Contributions

RBL designed the study, performed experiments, analyzed data and wrote the first version of the manuscript. CJR performed part of the cell experiments and aided in the design of the cell experiments. JH, YBK, and TJ performed part of the GUV experiments and analyzed data. CÅ supervised the cell work. WHR supervised the study. RBL, CJR, CÅ, WHR contributed to the writing of the final version of the manuscript.

## Supporting information



Supporting Information

Supplemental Movie 1

Supplemental Movie 2

Supplemental Movie 3

Supplemental Movie 4

Supplemental Movie 5

Supplemental Movie 6

Supplemental Movie 7

Supplemental Movie 8

Supplemental Movie 9

Supplemental Movie 10

Supplemental Movie 11

## Data Availability

The data that support the findings of this study are available from the corresponding author upon reasonable request.
