# Supplementary material for: Membrane Fusion‐Based Drug Delivery Liposomes Transiently Modify the Material Properties of Synthetic and Biological Membranes
Source: Small. 2025 Feb 25;21(12):2408039. doi: 10.1002/smll.202408039 (PMC11947515; doi:10.1002/smll.202408039)
Supplement: Supplementary file 1 — Supporting Information [file SMLL-21-2408039-s006.docx]

**Supplementary Information**

***Membrane fusion-based drug delivery liposomes transiently modify the material properties of synthetic and biological membranes***

*Jayna Hammond^1^, Ceri J. Richards^1,2^, YouBeen Ko^1^, Thijs Jonker^1^, Christoffer Åberg^2^, Wouter H. Roos^1*^, Rafael B. Lira^1,3*^*

*^1^Moleculaire Biofysica, Zernike Instituut, Rijksuniversiteit Groningen, Groningen, The Netherlands*

*^2^Pharmaceutical Analysis, Groningen Research Institute of Pharmacy, Rijksuniversiteit Groningen, Groningen, The Netherlands*

*^3^Current address: Department of Bionanoscience, Kavli Institute of Nanoscience, Delft University of Technology, Delft, The Netherlands*


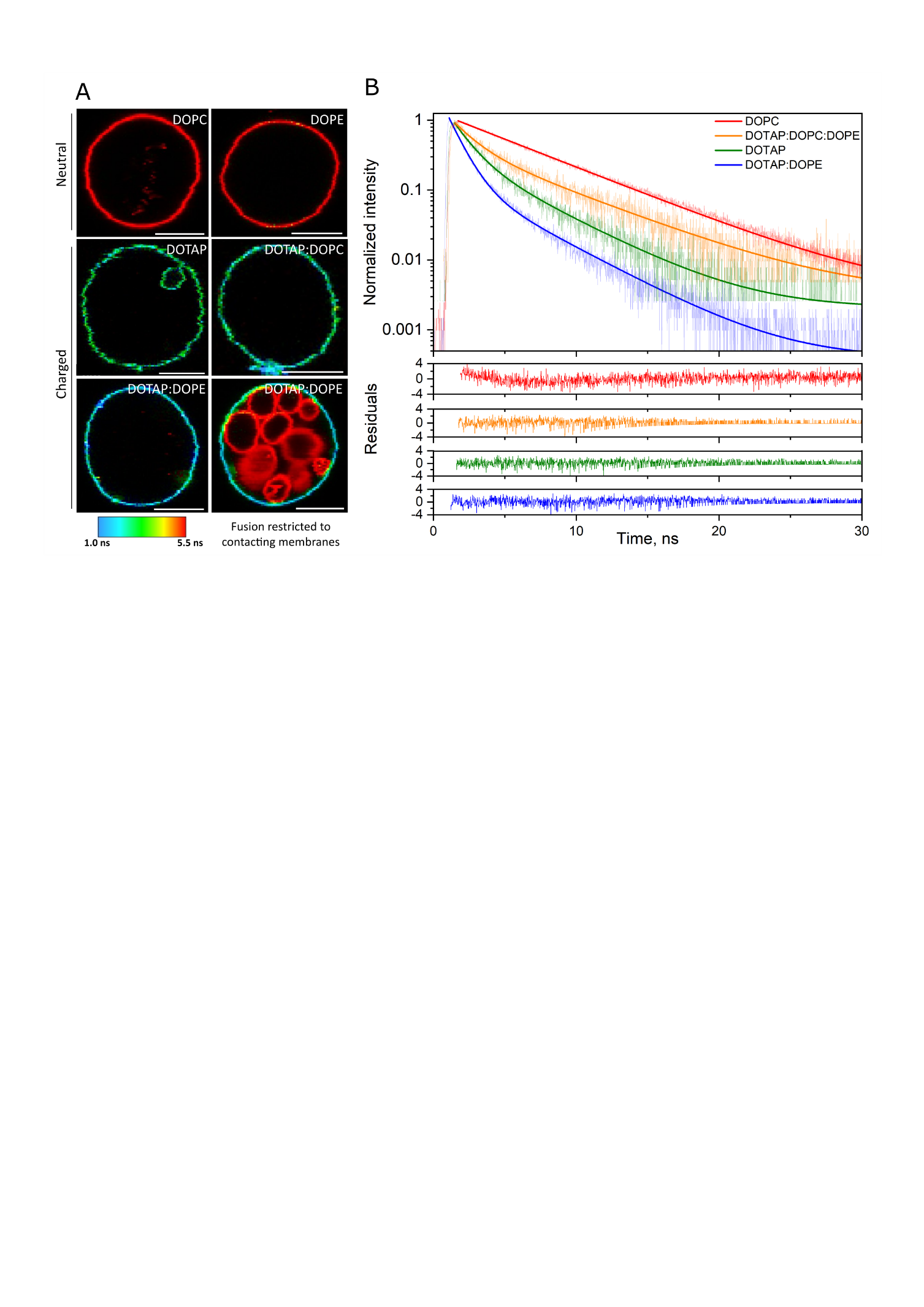


**Figure S1.** Representative lipid mixing experiments. Fusion of cationic liposomes based on DOTAP as the cationic lipid with GUV membrane models. A, representative DOPC:PS:Chol:DOPE (1:1:1:1, mole fraction) GUVs labelled with 0.5 mol% Bodipy C_16­_ upon incubation with 20 μM liposomes (lipid concentration). The specific liposome compositions (labelled with 2 mol% DPPE-Rh) are given in the respective image. Note that fusion is restricted to the contacting membranes, whereas intravesicular vesicles are not accessible and thus no decrease in donor lifetime is observed. Scale bars: 7 μm. B, representative Bodipy C_16_ fluorescence decay for some of the GUVs shown in A along with their respective fittings (solid lines). The residuals from the fits are also shown. For GUVs incubated with liposomes made of neutral lipids (e.g. DOPC), fitting was best adjusted with a single exponential decay, whereas for all other compositions, a two-exponential decay was used.


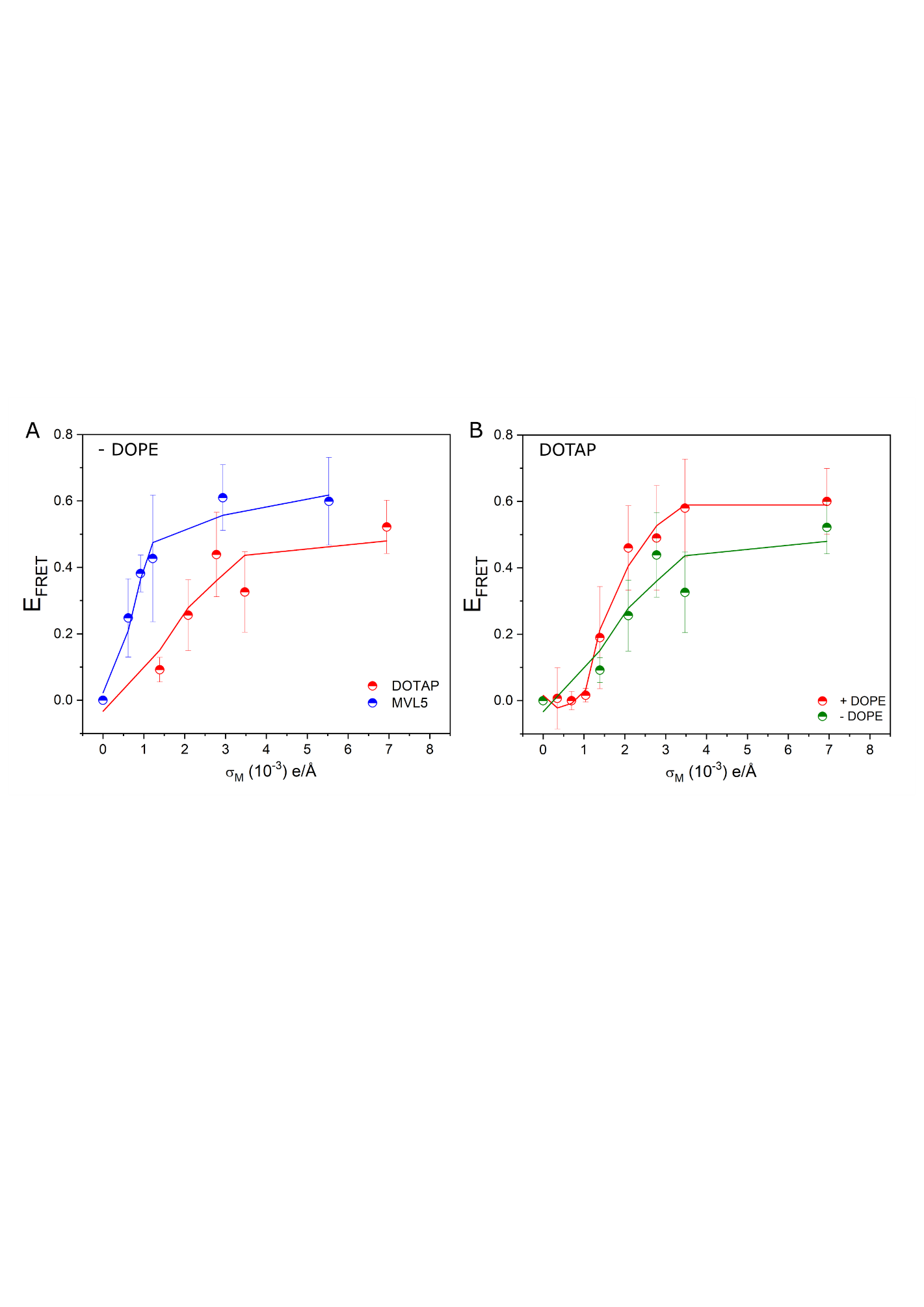


**Figure S2.** Fusion of cationic liposomes with GUV membrane models depends predominantly on membrane charge density (σ_M_). A, fusion response, as measured from E_FRET_, for liposomes containing DOTAP (red) or MVL5 (blue) as the CL (without DOPE helper lipid). B, E_FRET_ dependence on σ_M_ for liposomes containing DOTAP as the CL with or without DOPE as the helper lipid.

**
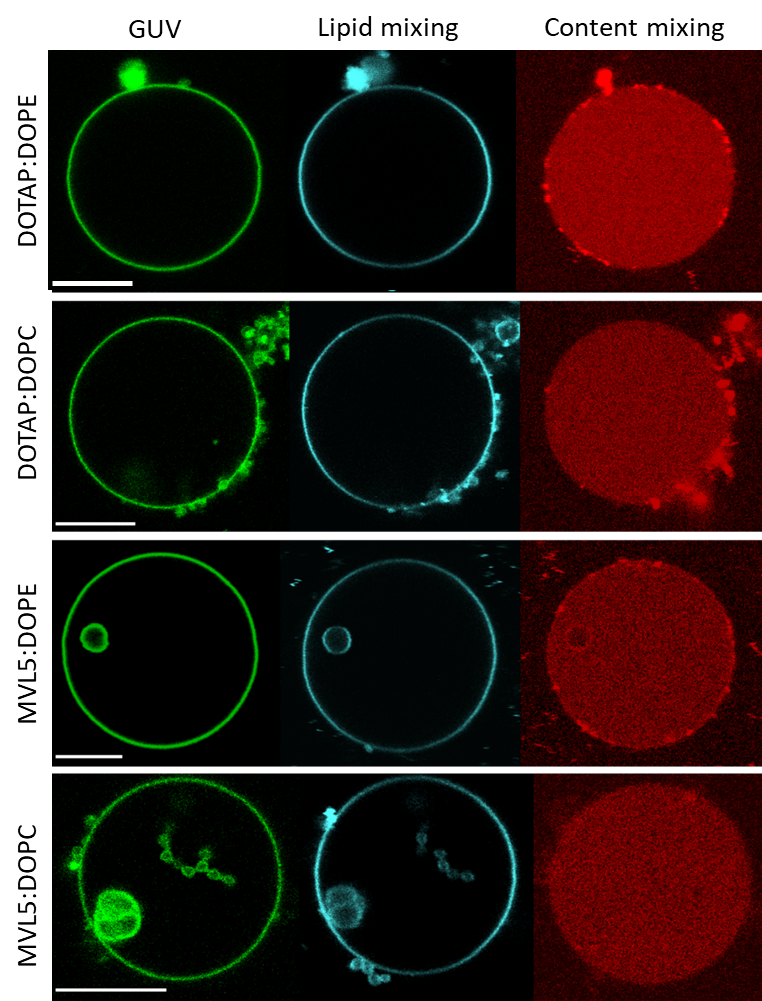
**

**Figure S3.** Cationic liposomes fuse with GUV model membranes via content mixing. The composition names refer to the liposomes that the GUVs (DOPC:DOPG:Chol:DOPE, 25:25:25:25, mole fraction) were incubated with. The GUVs are identified from their green fluorescence (labelled with Bodipy C_16_, 0.5 mol%). Fusion of the cationic liposomes, labelled at the membrane with 0.5 mol% Atto647-DOPE (cyan) and encapsulating 50 μM SRB (red) is identified by the appearance of the cyan signal at the GUV membrane, a sign of lipid mixing, as well as the SRB signal in the GUV lumen, a sign of content mixing. The experiments were done with 20-50 μM liposomes (lipid concentration). Scale bars: 10 μm.

**
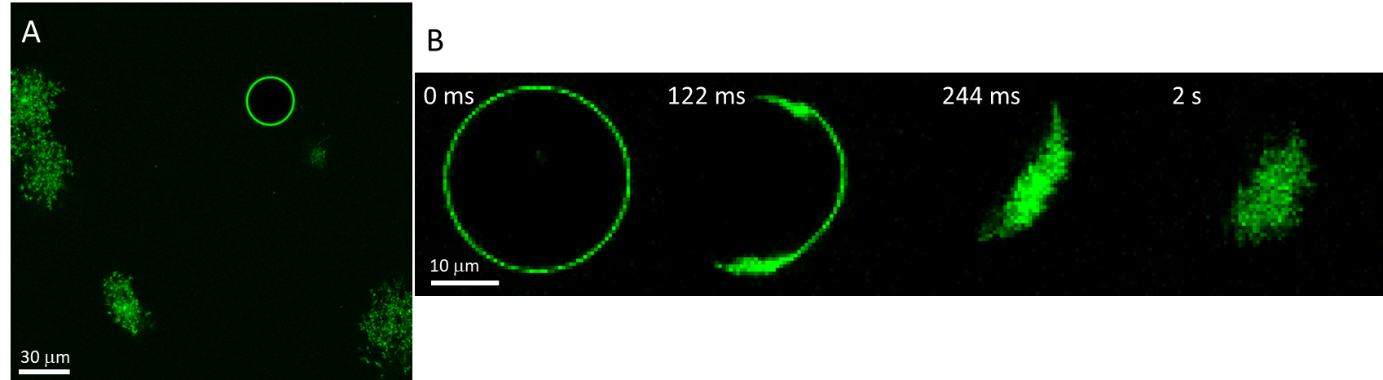
**

**Figure S4**. Liposome fusion induces GUV bursting. A, a remaining intact GUV surrounded by the debris of three burst GUVs made of DOPC:PS:Chol:DOPE (1:1:1:1, mole fraction) and labelled with 0.5 mol% Bodipy C_16­_ upon incubation with 20 μM (lipid concentration) MVL5:DOPC:DOPE (2.5:47.5:50 mole fraction) liposomes. B, single GUV bursting occurs by the stochastic opening of a pore (122 ms) that expands indefinitely by the conversion of flat-like membranes to tubular structures, leading to complete GUV collapse. In order to improve temporal resolution, only the GUV signal was recorded.


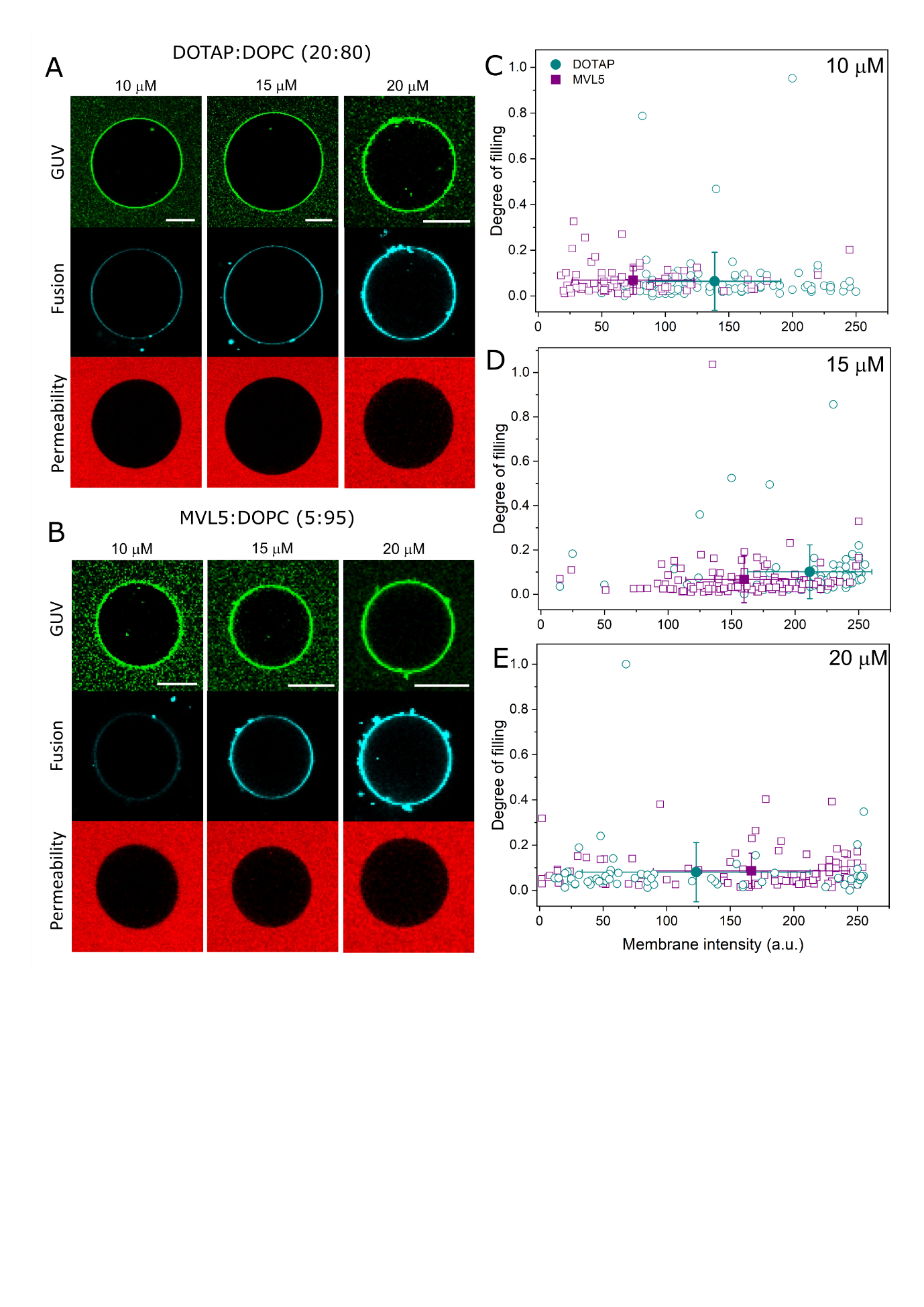


**Figure S5.** Effects of liposome concentration on fusion and fusion-dependent disruption of intermediate σ_M_ liposomes with GUVs. A and B, GUVs (DOPC:DOPG, 1:1 mole fraction) labelled with 0.5 mol% Bodipy C_16_ (green) incubated with liposomes made of DOTAP:DOPC (8:2 mole fraction) or MVL5 (5:95 mole fraction) at comparable σ_M_ labelled with Atto647-DOPE (cyan) in the presence of SRB as a leakage marker. Scale bars: 20 μm. C to E, measured degree of filling response as a function of fusion for DOTAP (green) or MVL5 (purple) liposomes at increasing liposomal concentration. Each data point represents a measurement on a single GUV. Means and standard deviations are also shown (solid symbols). Note that for most compositions, as the concentration increases, the data shifts to higher membrane signal (more fusion) but at these intermediate charge densities, fusion does not induce significant pore formation.

**
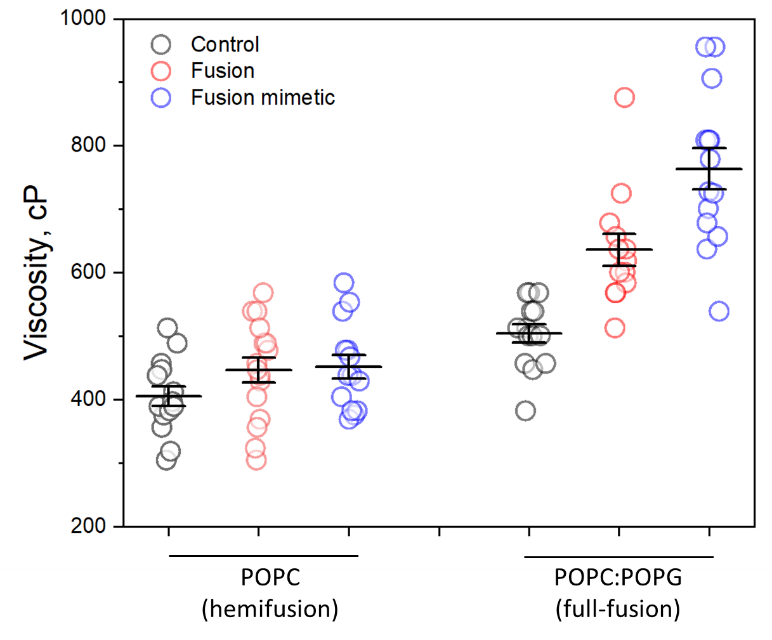
**

**Figure S6.** Calculated viscosity values for GUVs in the absence (control) and presence (fusion) of DOTAP:DOPE (1:1 mole fraction) liposomes. The liposomes are reported to undergo hemifusion with neutral POPC GUVs and full fusion with POPC:POPG (1:1 mole fraction) GUVs. Fusion mimetic, a composition that mimics that upon fusion saturation with the liposomes, are also reported. Note that fusion leads to an increase in membrane viscosity, an effect that is more pronounced upon full fusion. A further increase in viscosity is observed for the mimetic membranes. Each point represents a measurement on an individual GUV. Data calculated from the reported diffusion values from^1^. Means and standard errors of the mean are shown.


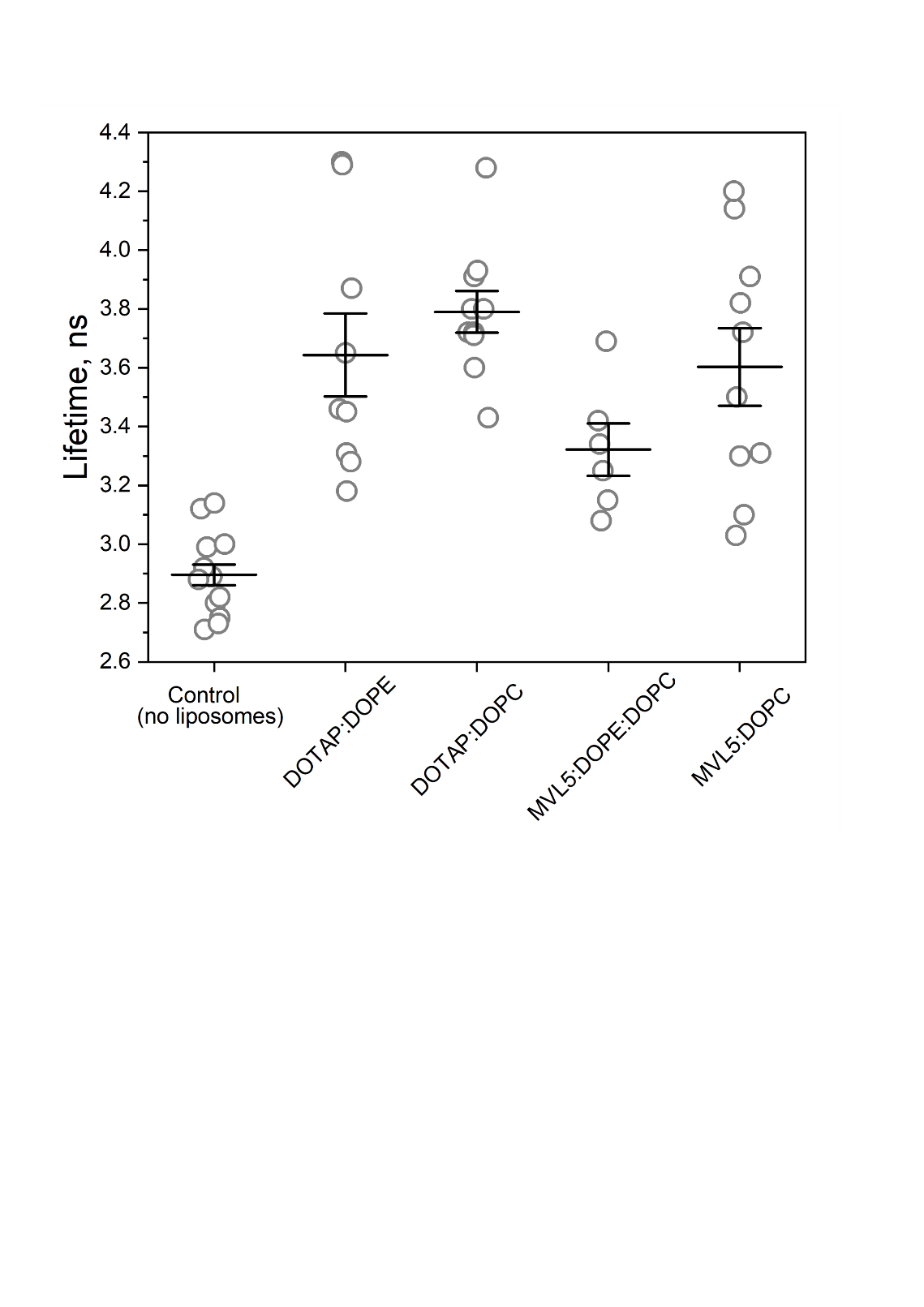


**Figure S7.** Fusion of liposomes of different compositions modifies membrane fluidity to a similar extent. The long fluorescence lifetime decay is shown for GUVs (DOPC:DOPG, 50:50 mole fraction) labelled with 1 mol% FliptR upon incubation with 10-15 μM liposomes containing different CL and helper lipids. Control GUVs in the absence of liposomes are also shown. Means and standard errors of the means are shown. Fusion increases FliptR lifetime regardless of liposome composition.


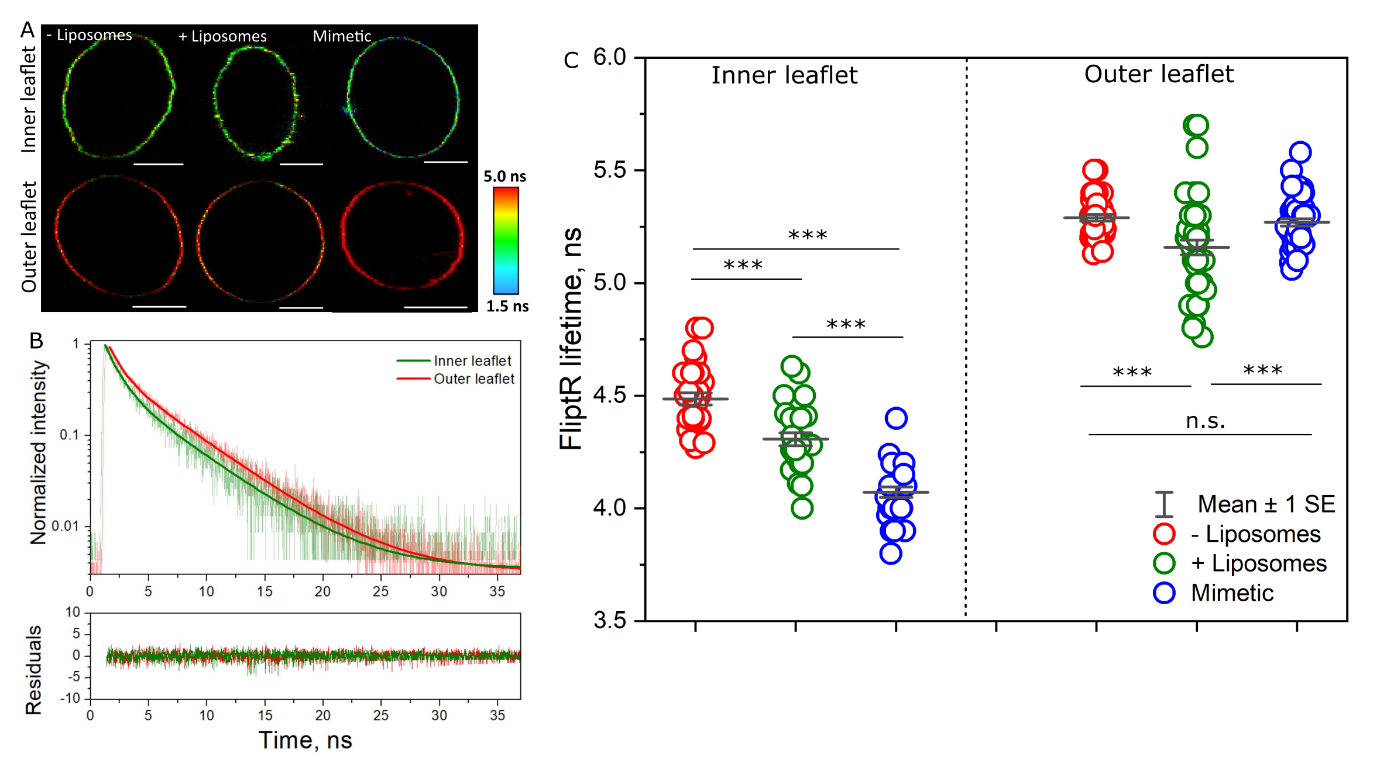


**Figure S8.** Liposome fusion modifies GUV fluidity depending on the final membrane composition. A, representative FLIM images of control GUVs mimicking the charged and fluid inner leaflet made of DOPC:PS:Chol:DOPE (25:25:25:25, mole fraction) or the nearly uncharged and ordered outer leaflet SM:DOPC:PS:DOPE:Chol (24:30:0.6:5.4:40) of the plasma membrane in the absence (- liposomes) or presence (+ liposomes) of non-labelled DOTAP:DOPE (1:1 mole fraction) 20 μM liposomes (lipid concentration). Alternatively, GUVs mimicking the expected composition after liposome fusion (upon saturation) were used as a reference. All GUVs were labelled with 0.5 mol% FliptR as a fluidity reporter. Note the differences in FliptR lifetime. Scale bars: 7 μm. B, representative fluorescence decays of fluid and ordered GUVs (- liposomes) are shown and best fit with a two-decay model. The respective fits and residuals of the fits are also shown. C, long lifetime of FliptR for the different compositions. Each circle represents a measurement on an individual GUV. Means and standard error of the means are shown. Statistical significance assessed by an independent two-sample *t*-test at a level of 0.05. The longer lifetime of control GUVs compared to the GUVs in Figure S7 is due to the more ordered membranes used here.


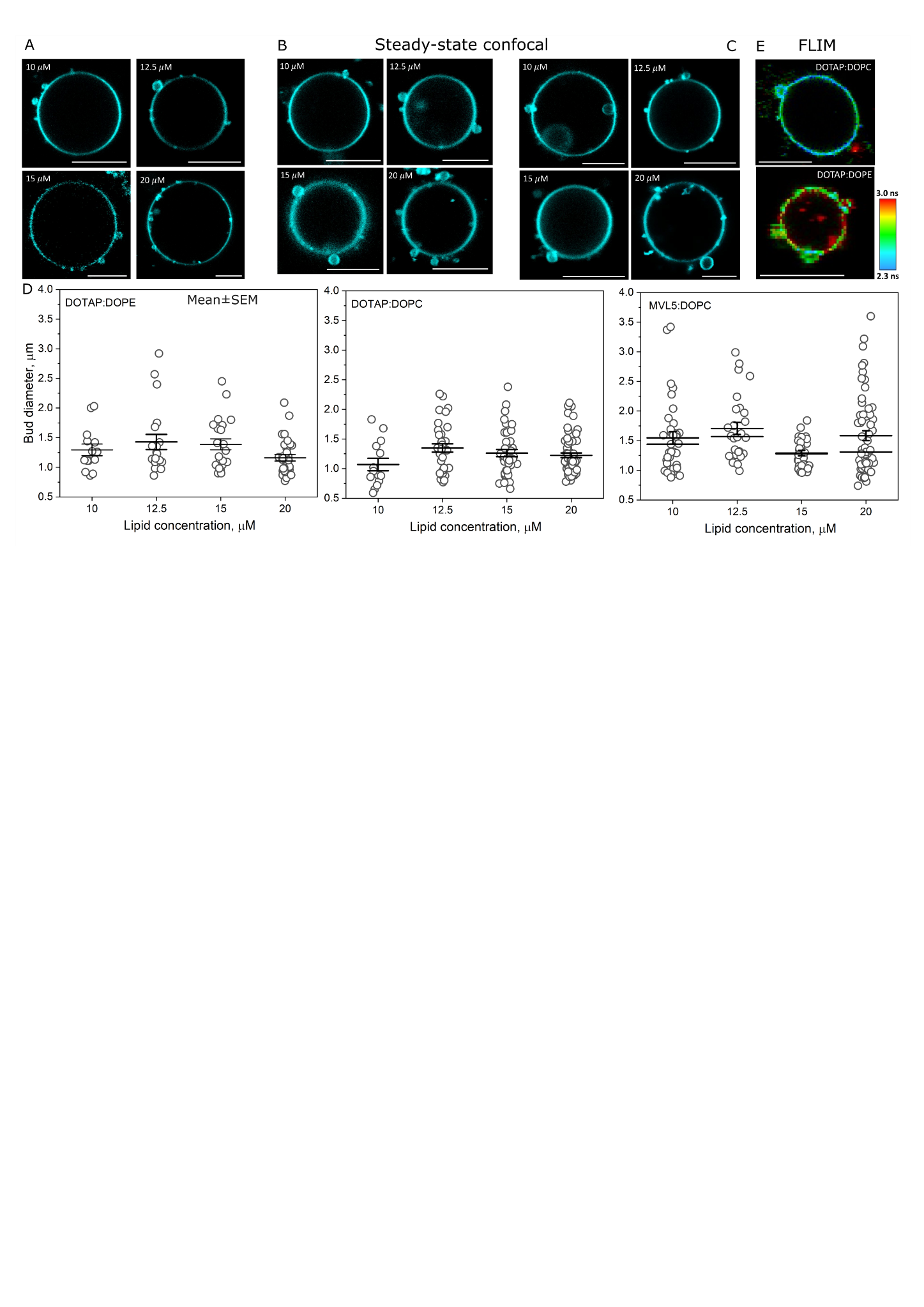


**Figure S9.** Liposome fusion induces GUV budding with similar composition to the flat membranes. A-C, steady state confocal images show representative DOPC:DOPG (1:1 mole fraction and labelled with 0.5 mol% Bodipy C_16_) GUVs containing buds after incubation with various liposome concentrations (total lipids) for DOTAP:DOPE (1:1 mole fraction), DOTAP:DOPC (1:1 mole fraction) and MVL5:DOPC (5:95 mole fraction). The signal comes from the Atto647-DOPE initially present in the liposomes (0.5 mol%) which is transferred to the GUVs upon fusion. Note that the buds are outwards, demonstrating positive curvature. D, bud diameter measurements for a number of GUVs for all conditions tested. Means and standard errors of the means are shown. The measurements were performed from equatorial cross-sections of GUVs, in which one or more buds could be seen per vesicle (with a maximum number of 3 buds for a given GUV, although these are relatively rare). Although single GUVs have multiple buds, most of them are out of focus, and only buds in focus were used for the measurement. The data shown in panel D are a combination of all buds measured, where occasionally multiple buds are reported from a single GUV, but in general > 5 GUVs (mostly > 10 GUVs) per condition. E, FLIM images of DOPC:DOPG GUVs labelled with 1 mol% FliptR and incubated with non-labelled DOTAP:DOPC (1:1 molar ratio) or DOTAP:DOPE (1:1 molar ratio). Note that the lifetime in the bud is comparable to that in the quasi-flat membrane of the GUVs, indicating similar viscosities, and hence equilibrated composition. All scale bars: 10 μm.


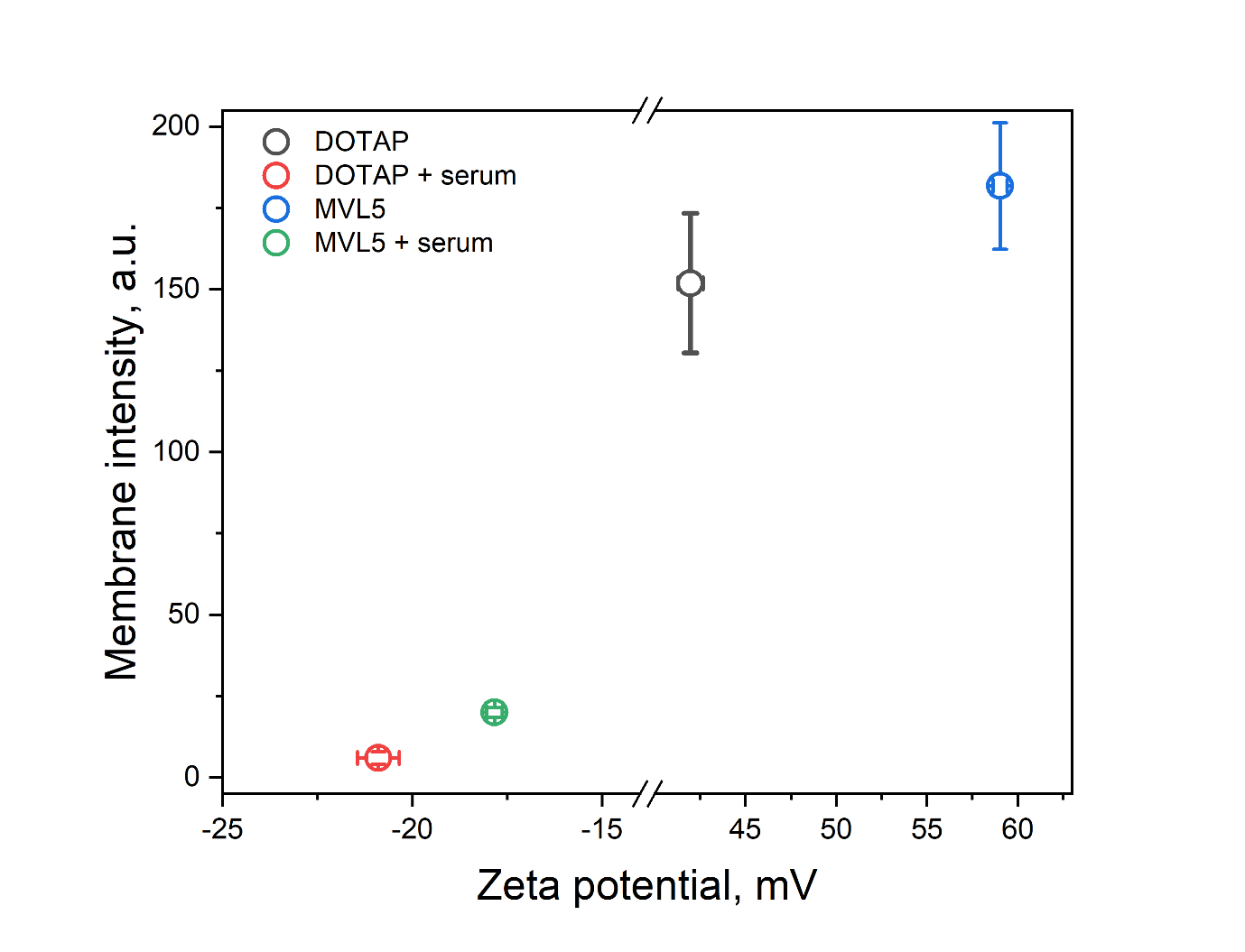


**Figure S10.** Serum binding decreases liposome surface charge, which prevents fusion. Atto647-DOPE membrane intensity, measured upon fusion of DOTAP:DOPC (20:80) or MVL5:DOPC (5:95) liposomes (20 μM total lipid) with DOPC:DOPG (50:50 mole fraction) GUVs as a function of liposome zeta potential for pristine liposomes and liposomes pre-incubated with serum. The data show mean and S.D.


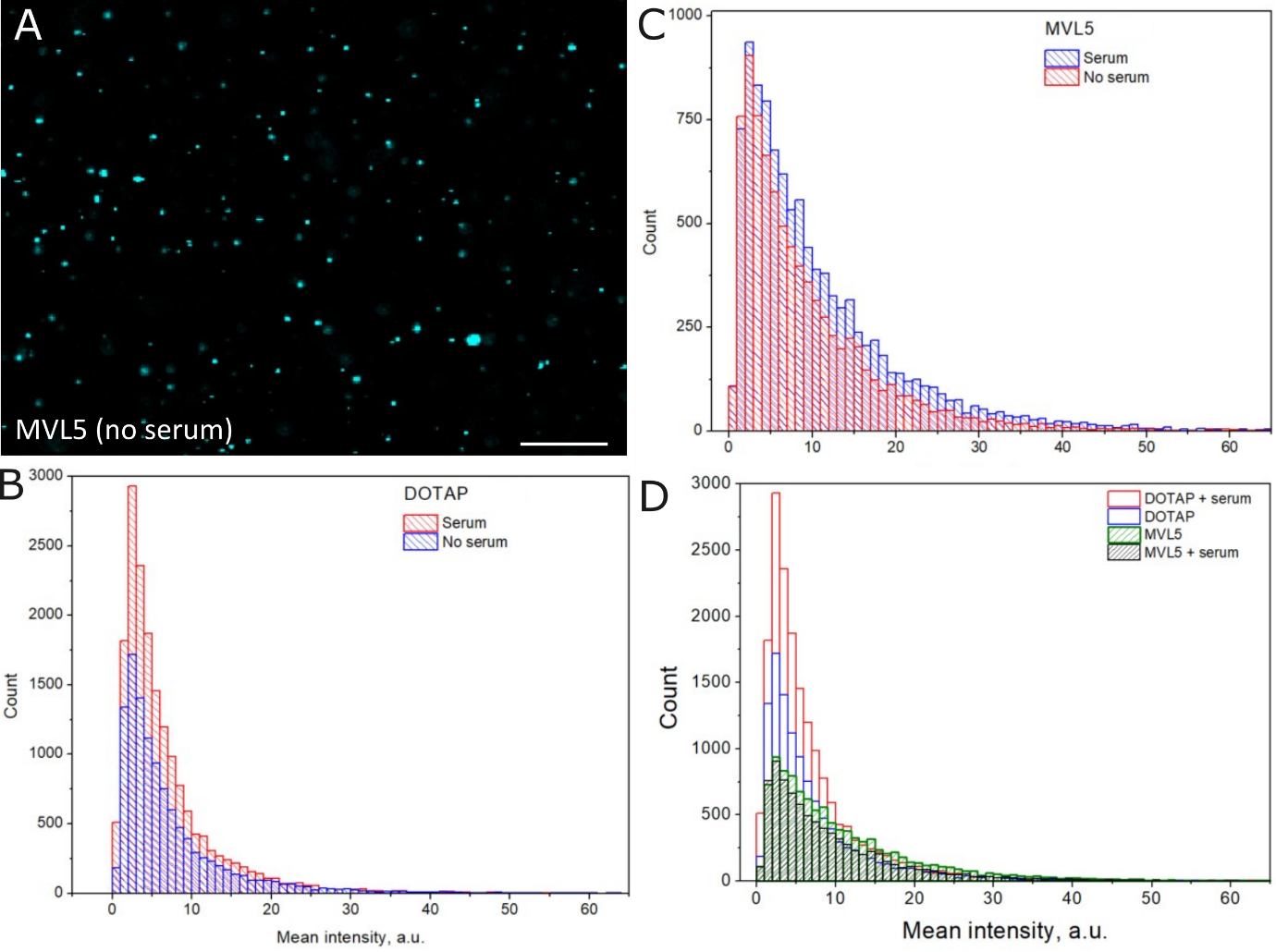


**Figure S11.** Incubation with serum does not modify liposome size distribution. A, representative confocal microscopy images of MVL5 liposomes in the absence of serum. The images show well-dispersed diffraction-limited spots of various sizes (as assessed from brightness). Scale bar: 30 μm. B and C, fluorescence intensity distribution of individual liposomes, assessed from images such as that shown in panel A, for pristine (blue) and serum-incubated (red) MVL5 and DOTAP liposomes, respectively. In D, all liposomes are shown for comparison. The data show total liposome counts. Compositions: DOTAP:DOPE:DOPC (20:50:3 mole fraction) and MVL5:DOPE:DOPC (5:50:45 mole fraction). Note that the intensity (i.e. size) distribution is nearly identical, with no signs of aggregation in the absence or presence of serum. All liposomes were labelled with 0.5 mol% Atto647-DOPE.


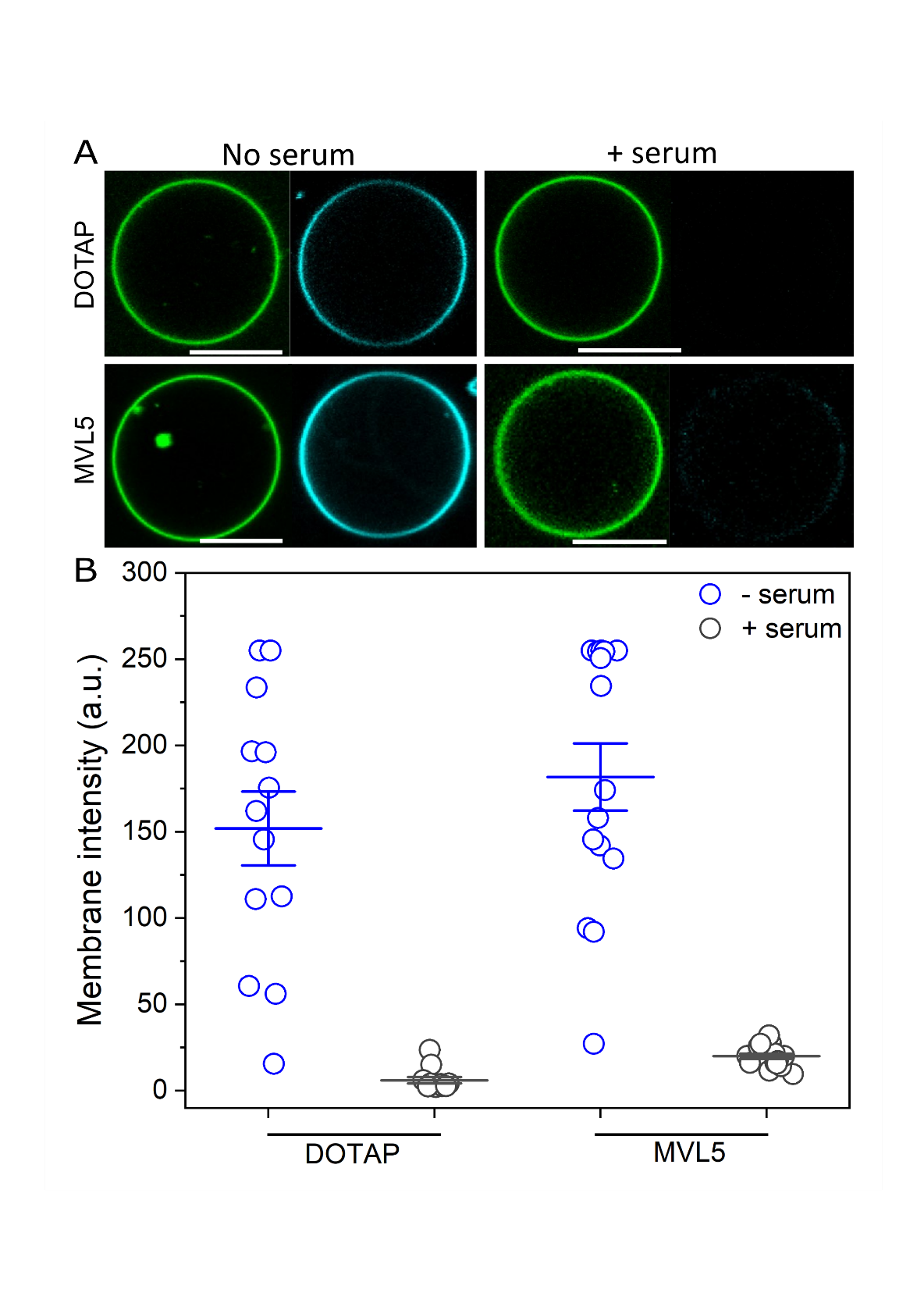


**Figure S12**. Protein-coated cationic liposomes are unable to fuse with GUVs. The GUVs made of DOPC:DOPG (1:1 mole fraction) were incubated with pristine liposomes, or liposomes that were pre-incubated with serum (10x diluted). The GUVs were labelled with 0.5 mol% Bodipy C_16_ and the liposomes were labelled with 0.5 mol% Atto647-DOPE (cyan). Fusion (lipid mixing) is detected as the appearance of a cyan signal in the GUV membrane. A, representative images of fusion of pristine liposomes containing DOTAP or MVL5 as the CL, and absence of fusion when the liposomes were pre-incubated with serum. Scale bars: 10 μm. B, single-GUV quantification of membrane signal. The means and standard error of the means are also shown.


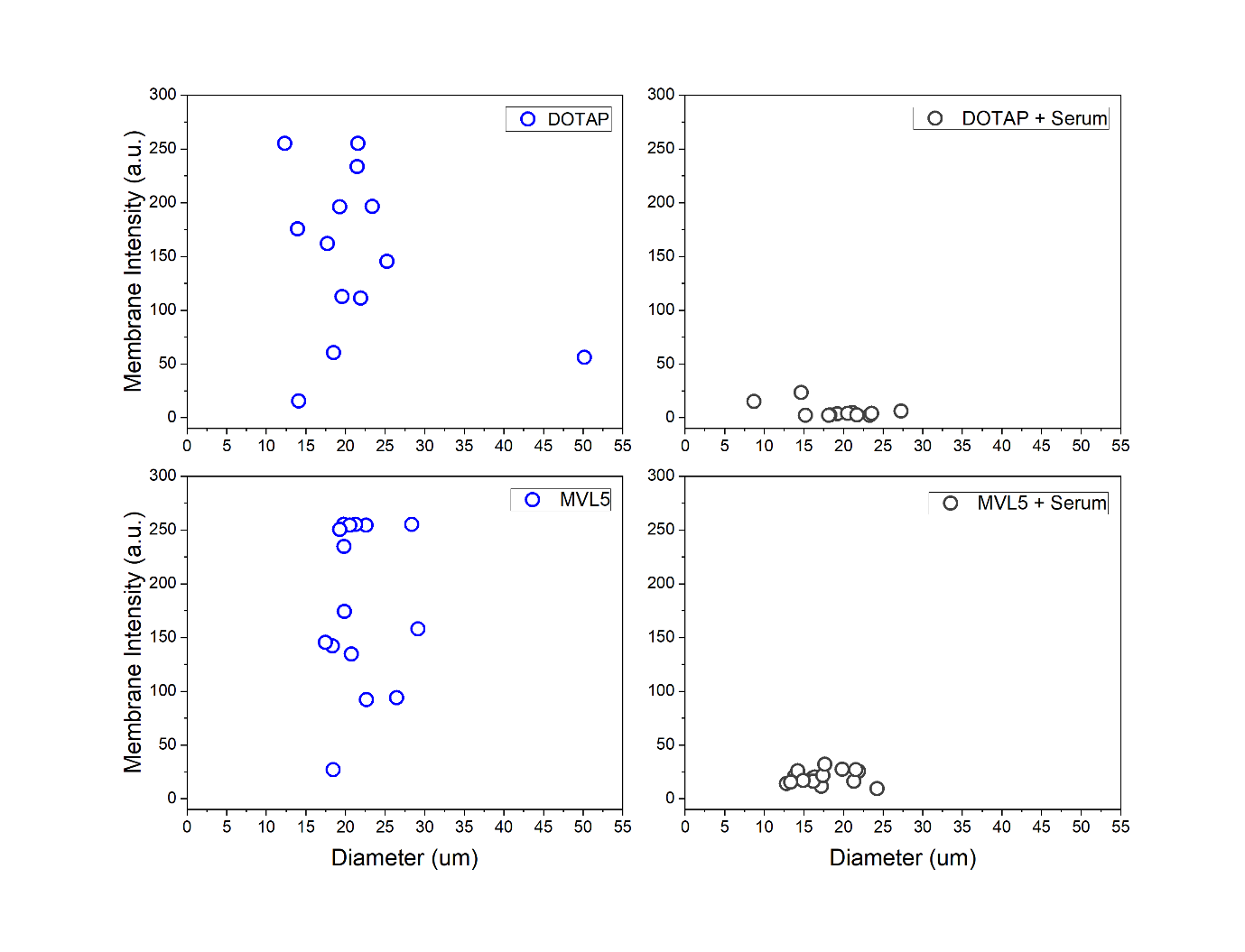


**Figure S13.** Fusion is not dependent on GUV diameter. The plots show the dependence of fluorescence Atto647-DOPE membrane intensity in DOPC:DOPG (1:1 mole fraction) GUVs upon fusion with cationic liposomes containing DOTAP (DOTAP:DOPE:DOPC - 20:50:30 mole fraction) or MVL5 (MVL5:DOPE:DOPC liposomes - 5:50:45 mole fraction) as the CL for pristine (blue) or protein-coated (black) liposomes. Each circle represents a measurement on an independent GUV.


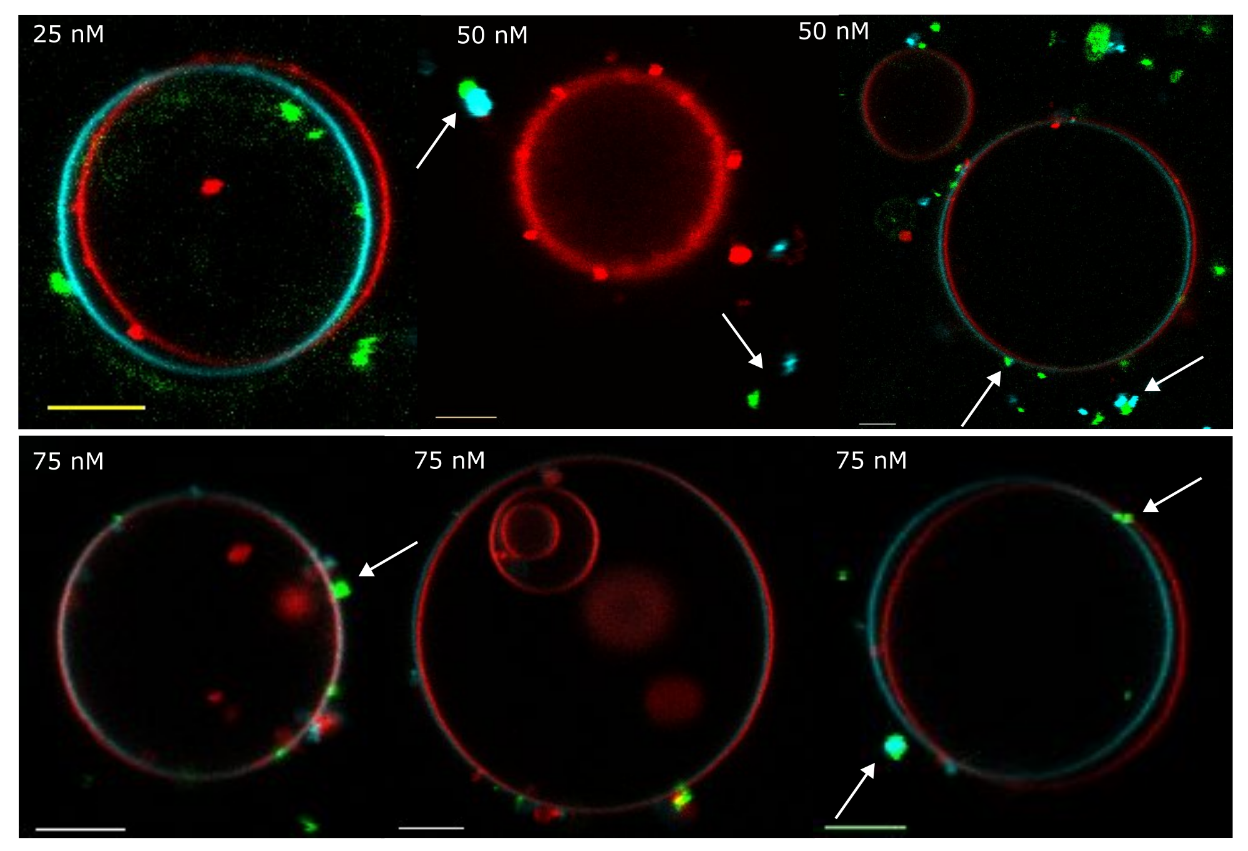


**Figure S14.** Albumin co-localizes with cationic liposomes. The images show representative snapshots of (DOPC:DOPG, 1:1 mole fraction) GUVs (labelled with 0.5 mol% DPPE-Rhodamine, red) upon incubation with 20 μM (lipid concentration) Atto647-DOPE (cyan, representing fusion) liposomes that have been pre-incubated with increasing concentrations of FITC-labelled albumin (green). The arrows point to co-localized cyan-green spots that correspond to albumin-liposome complex formation. Note that due to slow imaging (a few hundreds of milliseconds), GUV drift and various degrees of albumin/liposome mismatch are observed. Albumin concentrations are indicated. Scale bars: 5 μm.


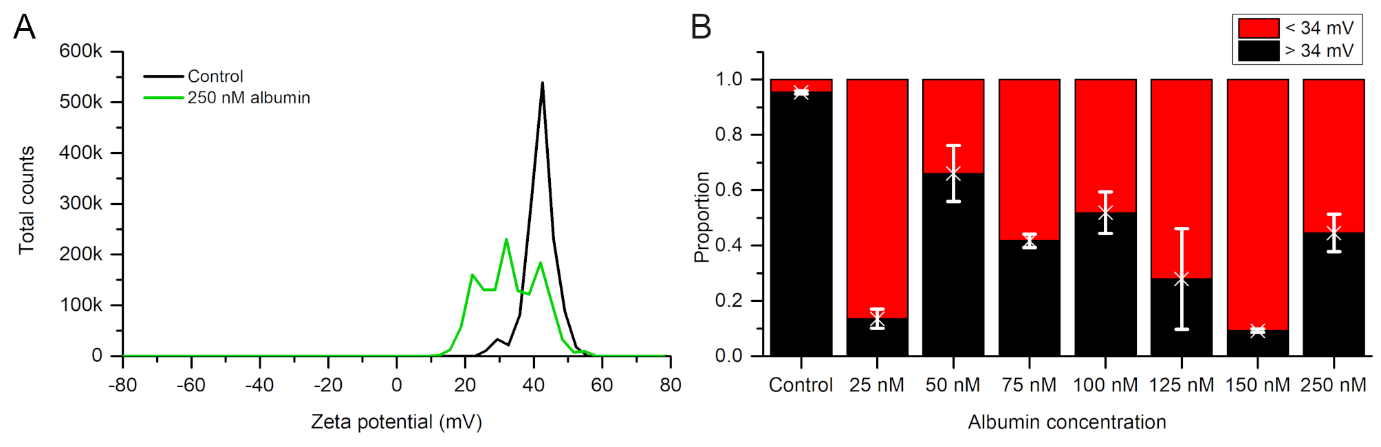


**Figure S15.** Zeta potential measurements of cationic liposomes made of DOTAP:DOPE (1:1 mole fraction) labelled with Atto647-DOPE incubated with increasing albumin concentrations. A, Zeta potential distribution of control liposomes (*i.e.* no albumin present, black) and liposomes incubated with 250 nM albumin (green). Control liposomes have a clear singular peak at 40 mV. While resolving subpopulations using zeta potential measurements is non-trivial, it would appear that liposomes incubated with albumin show sub-populations at lower zeta potentials as well as at 40 mV. B, Proportion of counts with zeta potentials above (black) and below (red) 34 mV for the various albumin concentrations shown in Figure 4. Control liposomes predominantly exhibit a single population with zeta potentials > 34 mV. For all albumin concentrations, substantial sub-populations of particles with lower zeta potentials (< 34 mV, red) are observed and therefore presumably had (partial) protein coatings. However, for all albumin concentrations, a population of liposomes still had zeta potentials similar to that of the control liposomes (black). Values are the mean and error (s.d.) for three measurements on the same samples as a function of albumin concentration.


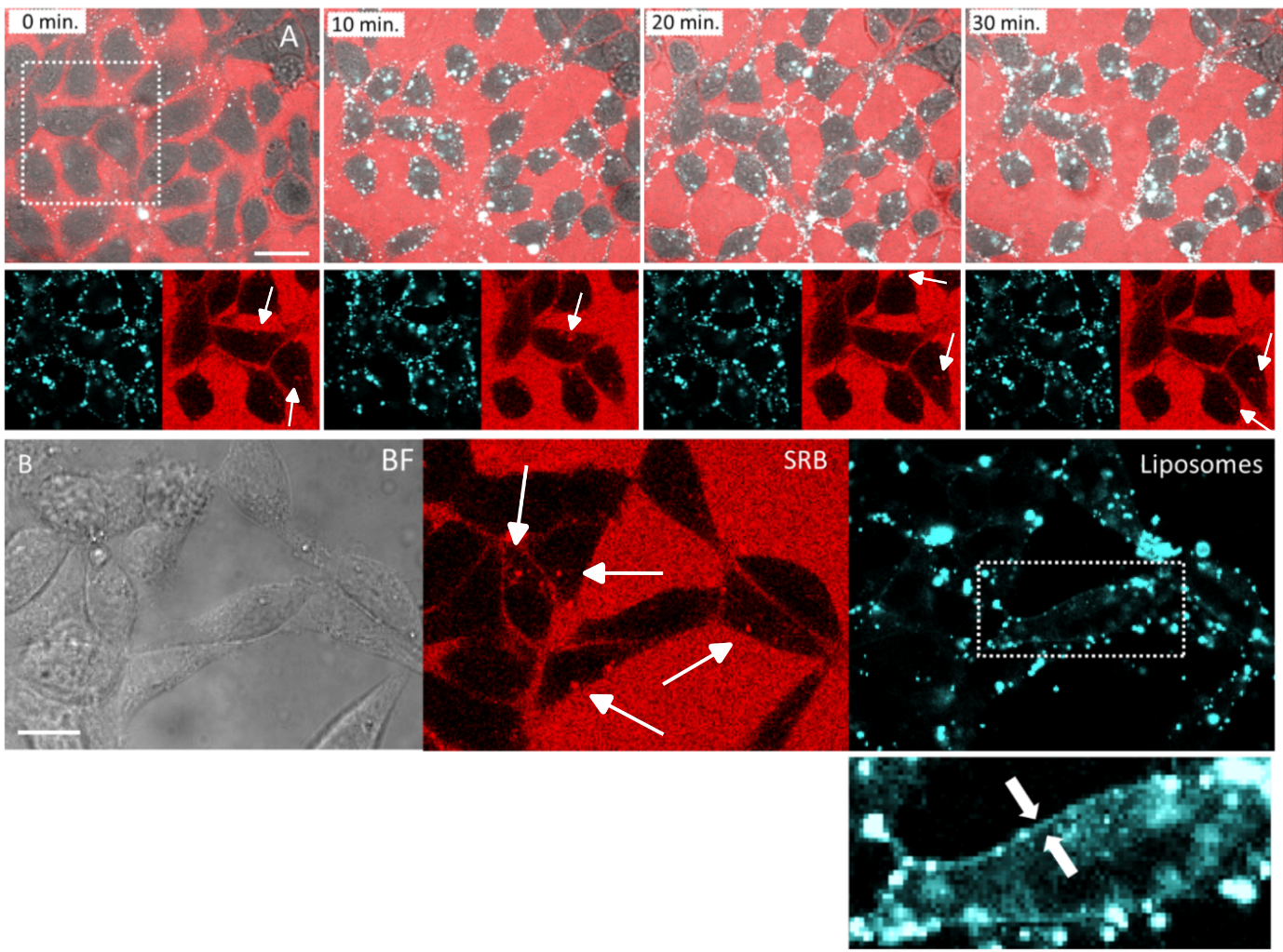


**Figure S16.** MVL5 liposomes do not efficiently fuse with the PM of living HEK cells. A, real-time visualization of incubation of pristine MVL5:DOPE:DOPC liposomes (5:50:45 mole fraction, 500 μM total lipids) labelled with 0.5 mol% Atto647-DOPE (cyan) in the presence of 10 μM SRB (red). The images are overlayed with bright field (BF, grey). The time points are relative to the onset of imaging (approximately half a minute post addition of liposomes to cells). The images below are zoom-in of the box in the upper row and show the co-labelling of liposomes and SRB presumably inside intracellular vesicles (arrows) after at least 30 minutes incubation with liposomes. Scale bar: 20 μm. B, bright field (BF), SRB and liposome channels of other HEK cells incubated with the liposomes. Note that although internalization seems to be a major mechanism of interaction for MVL5 liposomes, fusion may also take place, as judged from lipid mixing with the PM, albeit to a lower extent (arrows in the inset picture; brightness and contrast uniformly enhanced for better visualization). Fusion is assessed from uniform PM labelling, though in interpreting these results one should keep in mind the potential transfer of dye without fusion^2,3^ as well as dye released from internalized liposomes later returning to the PM. Scale bar: 10 μm.

**
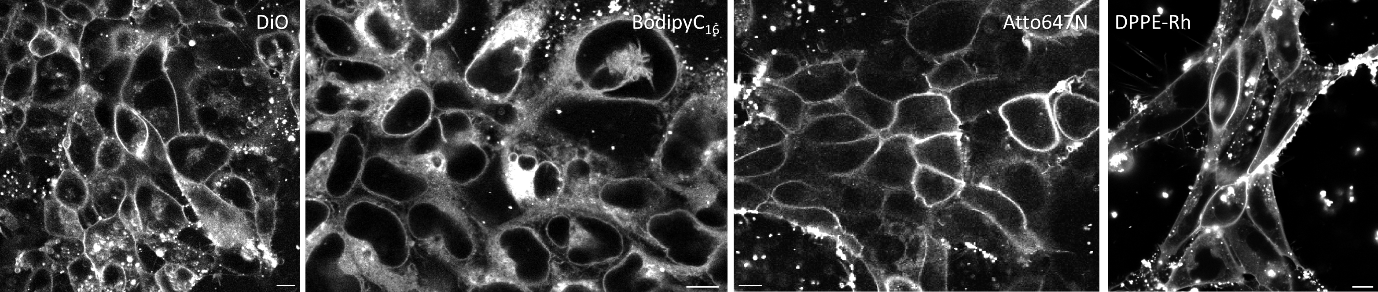
**

**Figure S17**. Different lipid markers have different intracellular localization upon fusion-mediated delivery. For all dyes, the cells were incubated with 500 μM DOTAP:DOPE (1:1 mol) liposomes (except BodipyC_16_, 200 μM). Scale bar: 10 μm.


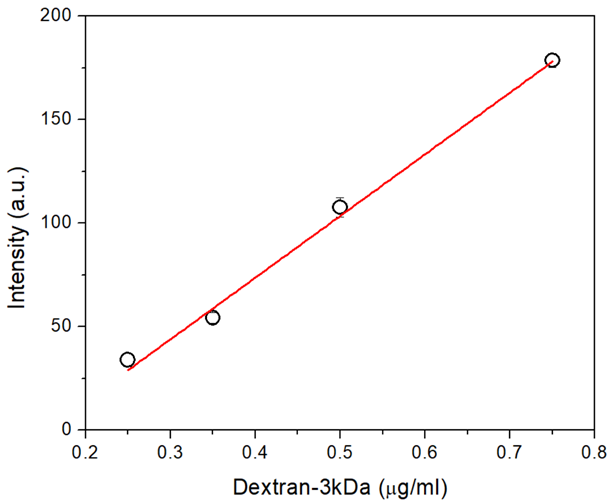


**Figure S18.** Determination of Dextran 3 kDa concentration from intensity measurements. Experiments performed from free dye in a 300 mM sucrose solution at room temperature.


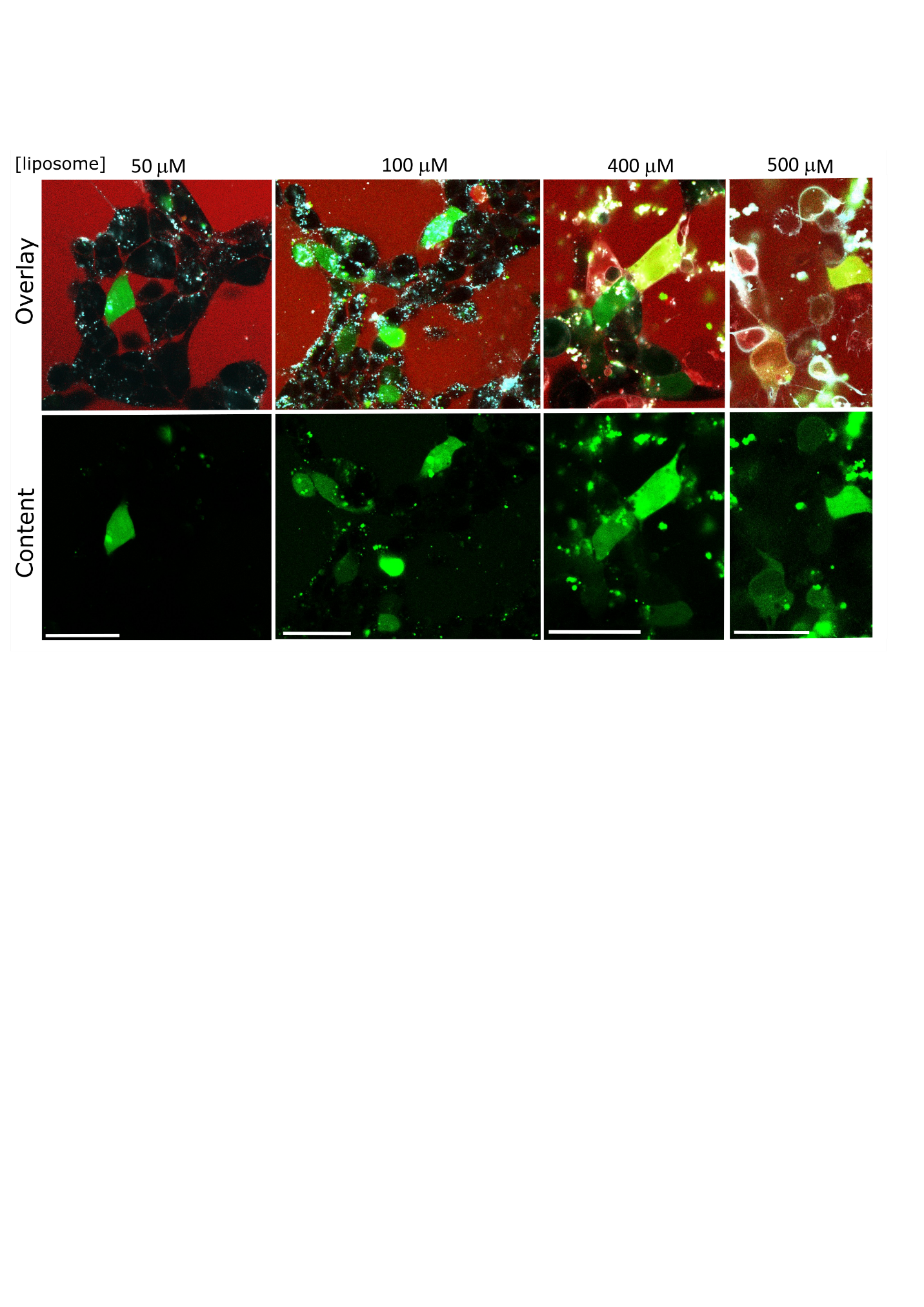


**Figure S19.** Cationic liposomes made of DOTAP:DOPE (50:50 mole fraction) at low concentrations (indicated in the images) induce heterogeneous intracellular delivery of water-soluble encapsulated Dextran 3 kDa in the absence of corona. The images show the overlay of the liposomes (cyan), the delivered cargo (green) and the leakage indicator SRB (red). A small be detectable number of cells exhibit a strong intracellular cargo signal. For these experiments, we deliberately searched for cells with Dextran signal and the imaging settings were set to be more sensitive than for the quantification experiments shown in Figure 5 in the main text. Note that at the higher liposomal concentrations, the cells start to become permeable to SRB (see Figure S20 for details). Scale bars: 30 μm.


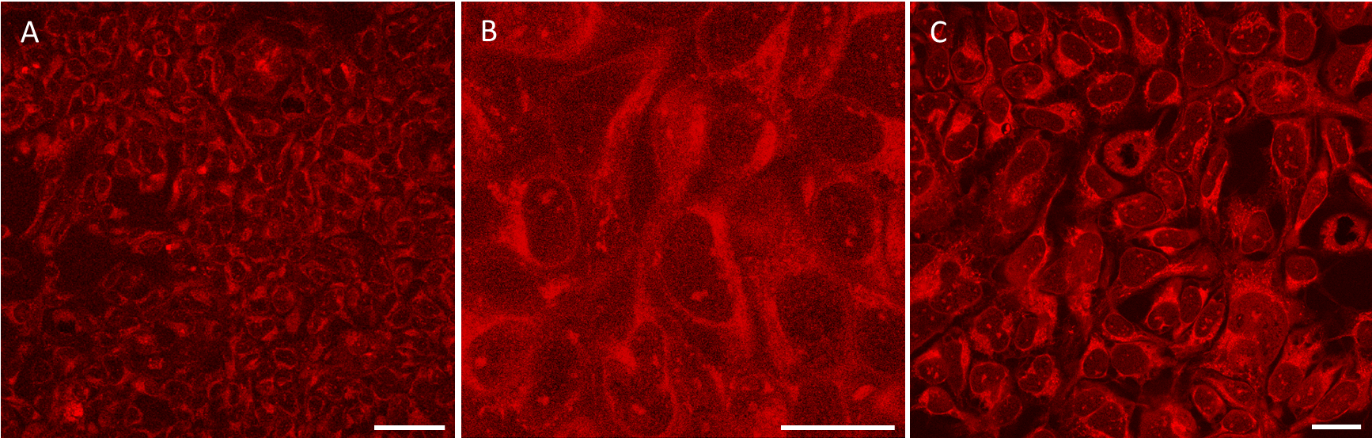


**Figure S20.** Non-specific SRB binding to intracellular structures in ethanol permeabilized cells. In A and B, cells were incubated with SRB for 30 minutes. In C, incubation for 2 h. Scale bars 20 μm.


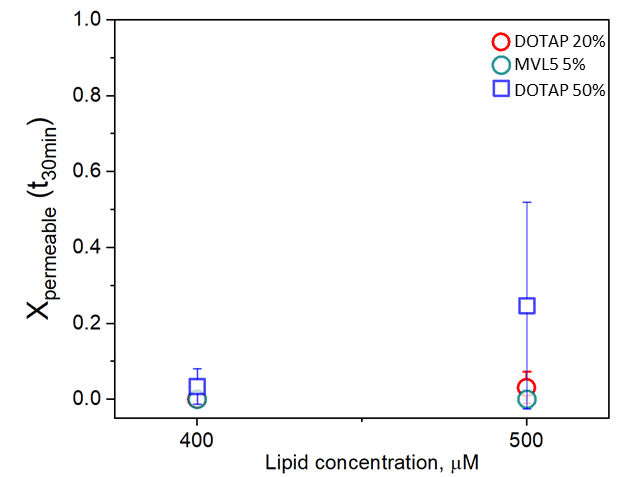


**Figure S21.** Liposomes permeabilize cells at high concentrations and high charge density. Fraction of cells permeable to SRB (X_permeable_) for DOTAP:DOPE:DOPC (20:50:30 mole fraction), DOTAP:DOPE (50:50 mole fraction) or MVL5:DOPE:DOPC (5:50:45 mole fraction). Each point represents the observation of tens of cells from at least two independent experiments. Means and s.d. are shown.


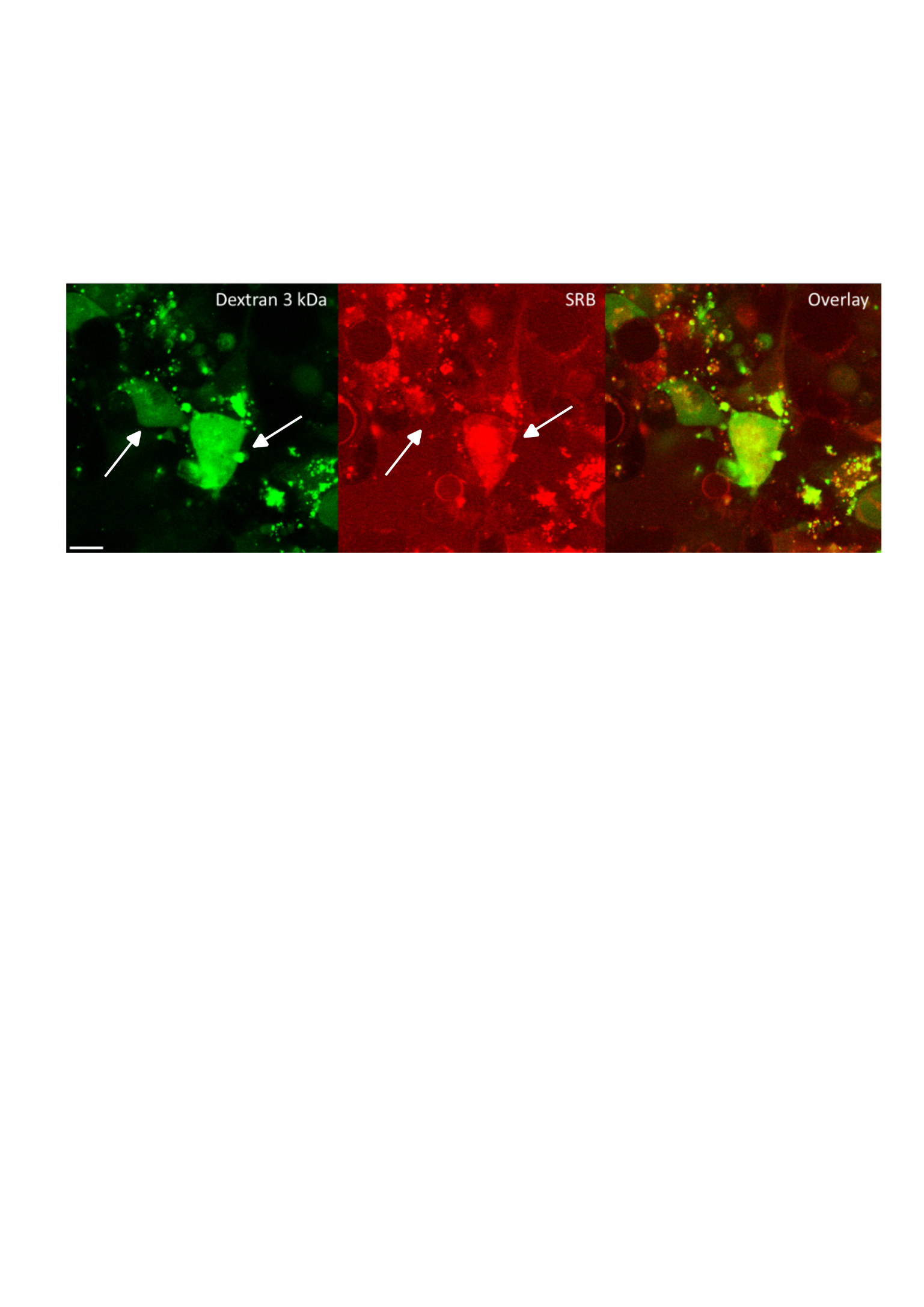


**Figure 22.** Delivered cargo remains entrapped in cells whose plasma membrane becomes permeable to a small probe. Dextran 3 kDa delivered cargo is retained in cells that become permeable (arrows) upon fusion of DOTAP:DOPE liposomes (50:50 mole fraction), 500 μM total lipid concentration. The SRB channel shows that these cells become permeable, but the pores formed are only small enough to allow SRB entry without leading to Dextran 3 kDa escape. The rightmost image shows the overlay of both channels. Scale bars 10 μm.


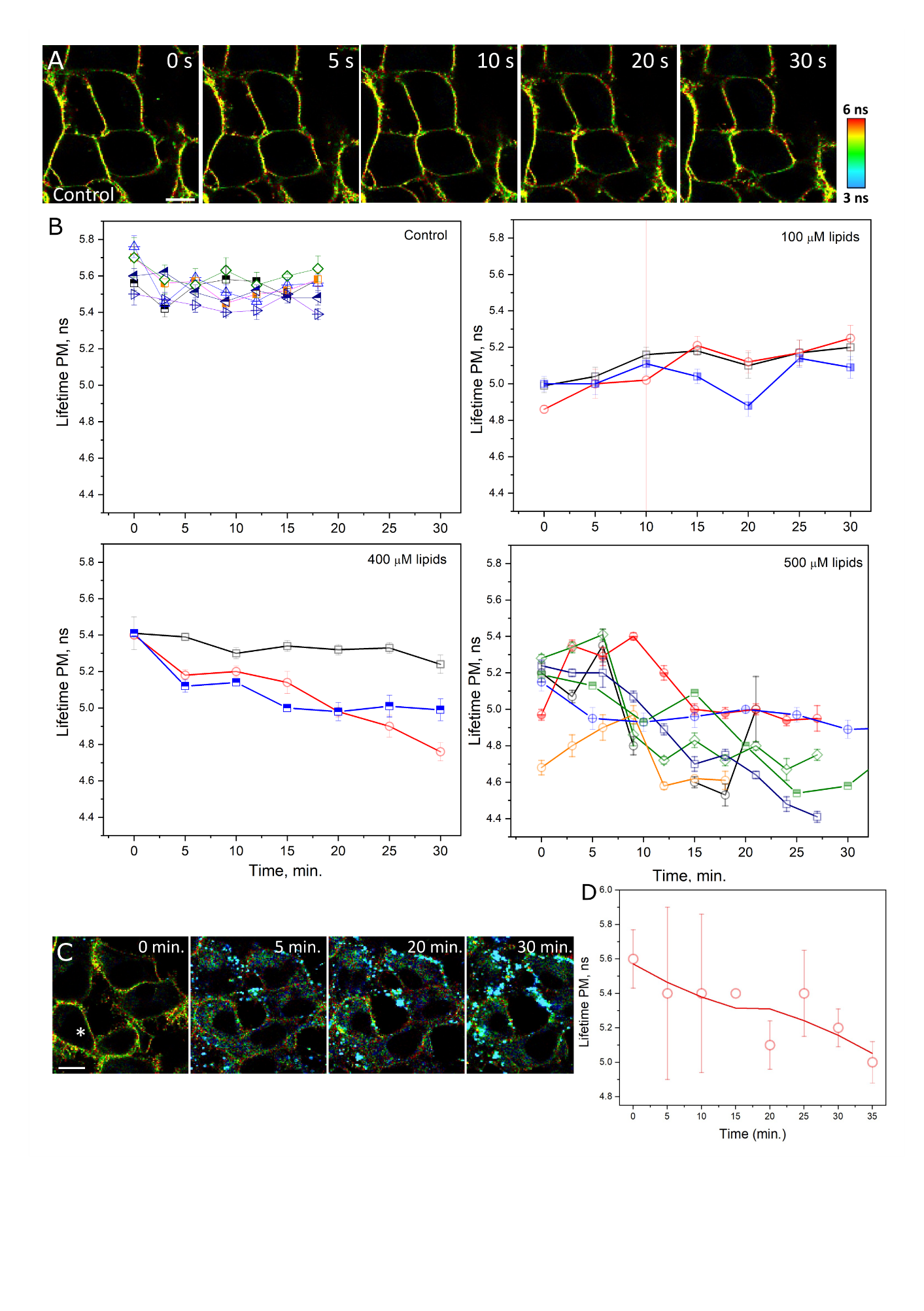


**Figure S23.** Changes in PM packing are dependent on liposomal concentration. A, stable PM labelling and lifetime in control cells in the absence of liposomes. B, single cell lifetime changes (long lifetime) measured at the PM for cells incubated with DOTAP:DOPE (1:1 mole fraction) liposomes at increasing liposome (total lipid) concentration. Each line represents the temporal evolution of a single cell. Error bars represent the errors from the fluorescence decay fit. C, a group of cells incubated with 500 mM (total lipids) liposomes composed of DOTAP:DOPE:DOPC (20:50:30 mole fraction). D changes in FliptR’s long lifetime measured on the plasma membrane for the cell in panel C indicated by an asterisk *. Scale bars: 5 μm.


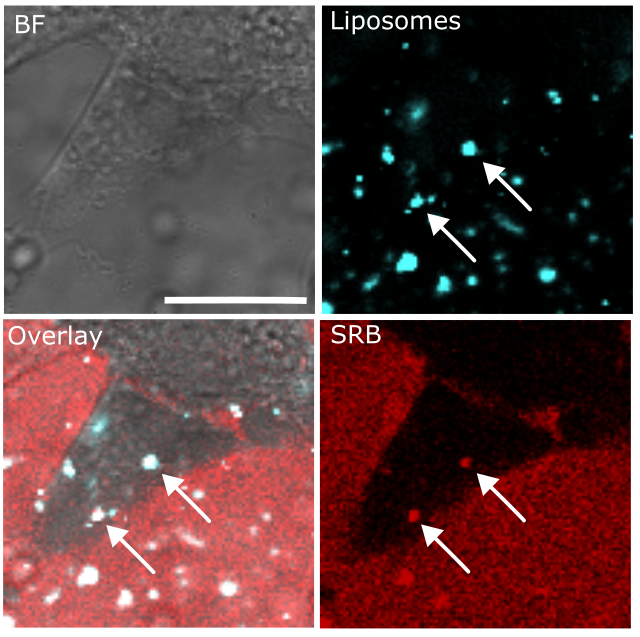


**Figure S24.** Liposomes composed of MVL5:DOPE:DOPC (5:50:45 mole fraction), labelled with 0.5 mol% Atto647-DOPE (cyan), were incubated with living HEK cells at 500 mM (total lipid concentration), in the presence of 10 μM SRB (red), after pre-incubation in 10x diluted serum. The arrows point to co-localized spots between liposomes and SRB. Scale bar: 20 μm.


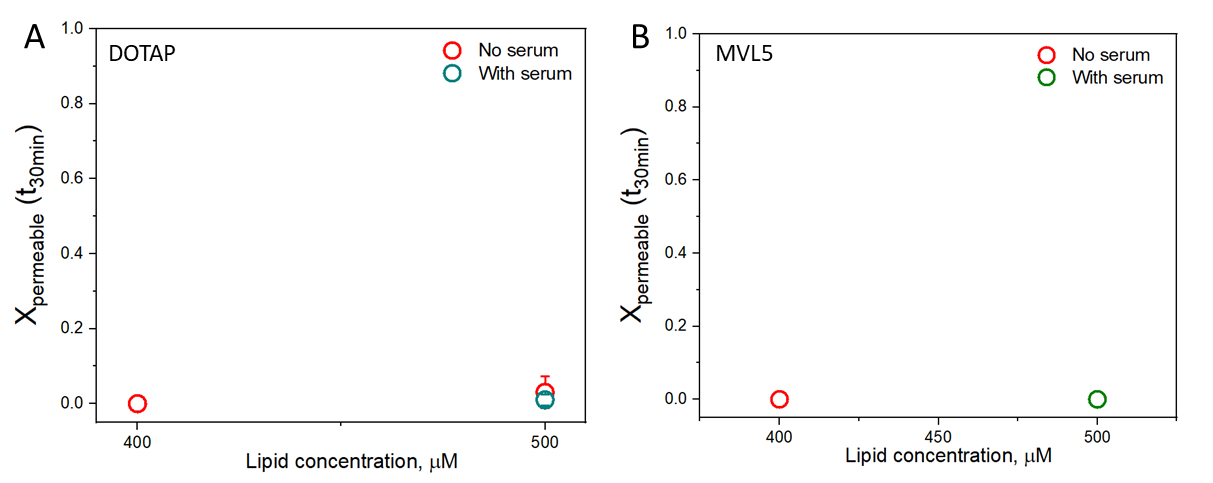


**Figure S25.** Cationic liposomes of intermediate σ_M_ do not permeabilize the PM of HEK cells. A and B show the fraction of permeable cells (X_permeable_) upon incubation with the highest liposome concentrations tested, for DOTAP:DOPE:DOPC (20:50:30 mole fraction) and MVL5:DOPE:DOPC (5:50:45 mole fraction), respectively, both labelled with 0.5 mol% Atto647-DOPE. The experiments were performed for pristine liposomes and liposomes that had been pre-incubated in 10x diluted serum. Each point represents the observation of tens of cells from at least two independent experiments. Means and s.d. are shown.


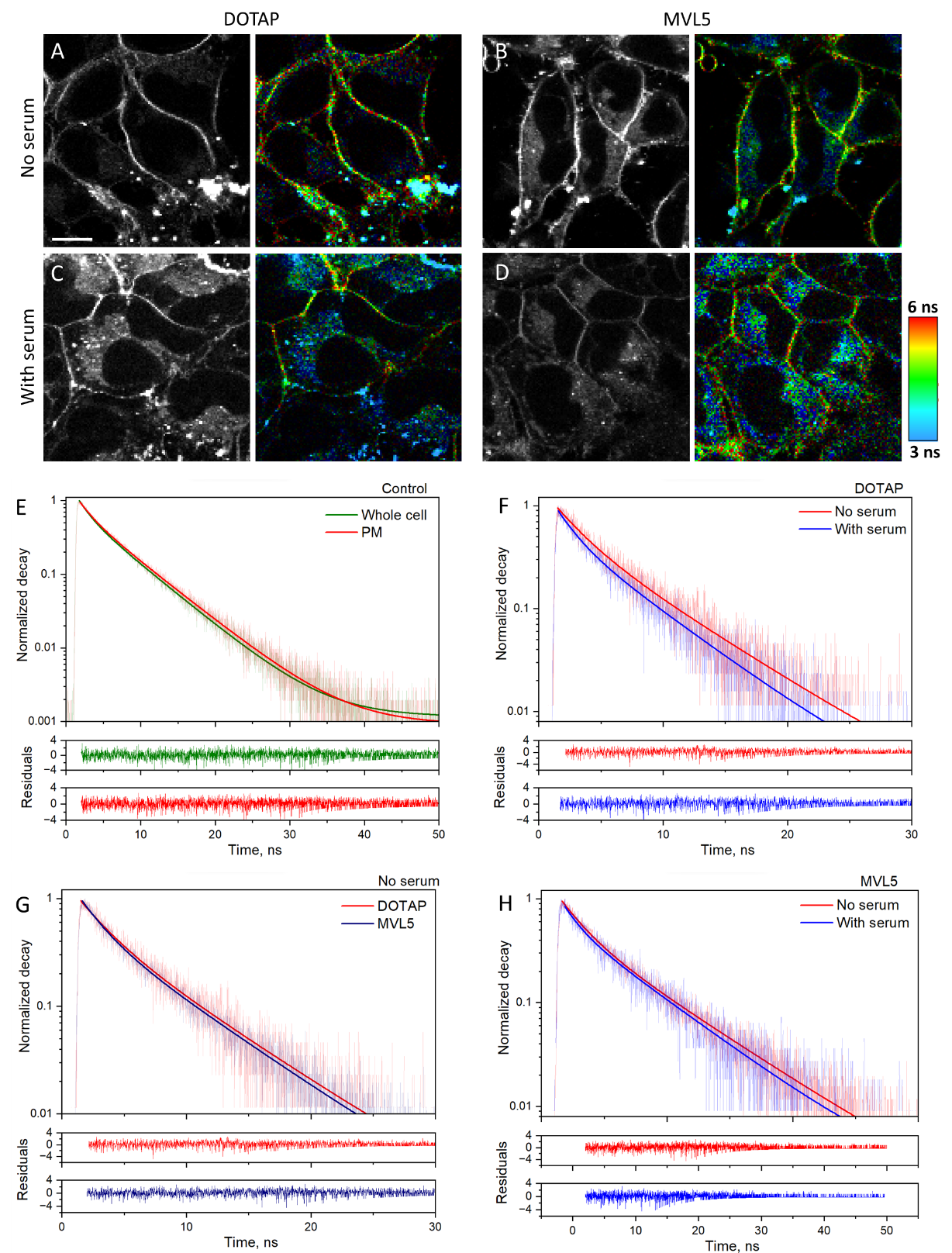


**Figure S26.** Effects of cationic liposomes containing DOTAP or MVL5 as the CL and pre-incubation of serum on PM fluidity. A-D, representative fluorescence intensity (left) and FLIM (right) images of HEK cells incubated with 500 μM (total lipid concentration) non-labelled DOTAP:DOPE:DOPC (20:50:30 mole fraction) or MVL5:DOPE:DOPC (5:50:45 mole fraction) for pristine (no serum) or protein-coated liposomes pre-incubated with 10x diluted serum. The cells were labelled with 1 mM FliptR. Note the predominant accumulation of the probe mainly at the PM. Shorter lifetime intracellular labelling is also observed. Scale bar: 10 μm. E, measured lifetime at the whole cell level or only at the level of the PM for control samples (no serum, no liposomes). F to H show fluorescence decays measured at the PM level for some representative cells incubated with DOTAP or MVL5 liposomes that either have or have not been pre-incubated in serum. For all plots, the fluorescence decays, fits and residuals of the fits are shown.


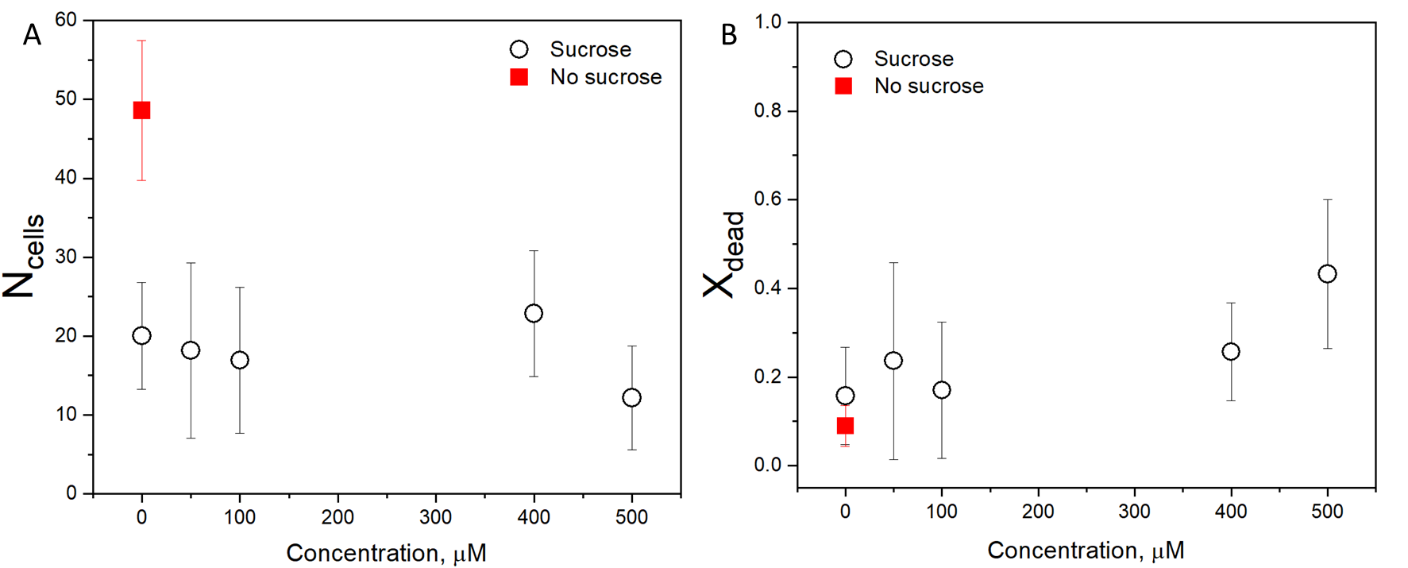


**Figure S27.** Incubation of living HEK cells with cationic liposomes reduces the number of cells. A, average number of live cells counted in one quadrant of a Neubauer hemacytometer after incubation with increasing concentrations of DOTAP:DOPE (50:50 mole fraction) liposomes. Four quadrants were measured per triplicate measurement. Reported values and errors are the means and standard deviations across all measurements (12 measurements in total). B, fraction of dead vs. live cells as a function of increasing liposomal concentration. The concentrations refer to total lipid concentration. Incubation was carried out in sucrose 200 mM (open circles), or in complete cell medium (solid red square). Note that though sucrose treatment did not increase the dead fraction of measured cells, the total number of cells was reduced, likely due to cell detachment during the washing steps. Thus, the effects of sucrose seem to be more prominent than the liposomal treatment per se. Reported values and errors are the means and standard deviations of measurements of 4 quadrants for triplicate experiments (12 measurements in total).

**
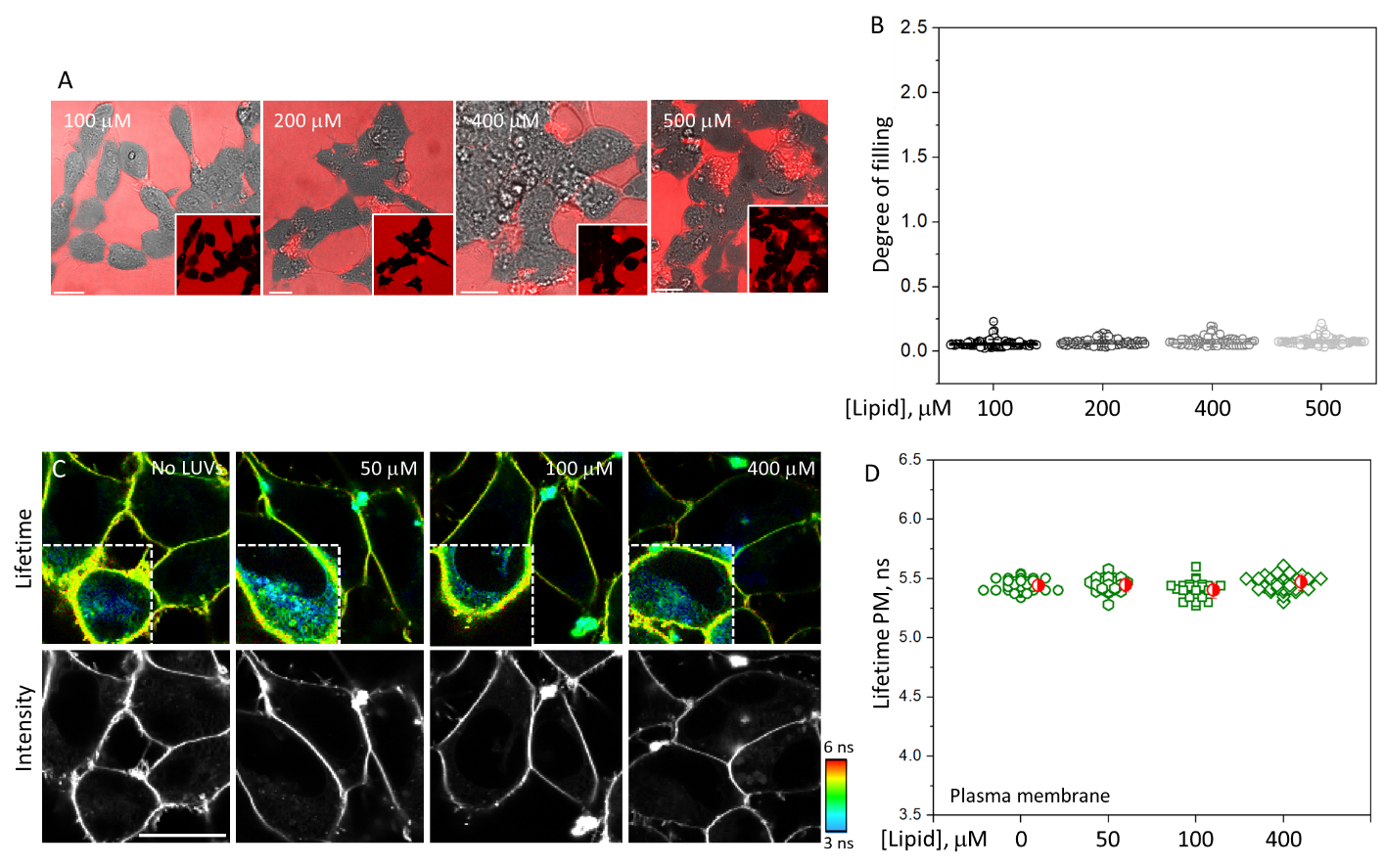
**

**Figure S28.** Effects of liposome treatment on PM permeability and fluidity are transient. A, representative living HEK cells that had been incubated with increasing concentrations of DOTAP:DOPE (50:50 mole fraction) liposomes for 30 minutes, washed to remove the liposomes and placed back in cell culture conditions for 24 h to allow recovery. Imaging was performed in the presence of 10 μM SRB to assess PM permeabilization. B, single cell measurements of the degree of filling. Note that all cells that survived treatment had intact PMs. Around 100 cells analysed. C, representative FLIM (upper row) and fluorescence intensity images (bottom row) of cells that had been incubated with increasing concentrations of the same liposomes as in A and B and allowed to recover. The cells were labelled with FliptR. The probe mainly localizes at the PM, although lower lifetime (more fluid) intracellular organelles can be seen (insets). From the images, it is clear that intracellular labelling is weaker compared to acute liposome treatment (Figure 6D in the main text). D, measured FliptR fluorescence lifetime (long lifetime) at the PM for a number of cells (30-50) for increasing liposome concentrations. Scale bars in A: 20 μm. Scale bars in B: 10 μm.

**Supplementary movies**

**Movie S1.** Bursting of GUV model membrane upon fusion of cationic liposomes. The GUVs made of DOPC:PS:Chol:DOPE (1:1:1:1 mole fraction) and labelled with 0.5 mol% Bodipy C_16­_ were incubated with 20 μM (lipid concentration) liposomes (MVL5:DOPC:DOPE, 2.5:47.5:50 mole fraction). Only the green channel is shown. The numbers correspond to the time from the onset of observation.

**Movie S2.** Time-lapse of liposomes containing MVL5 as the CL (MVL5:DOPE:DOPC – 5:50:45 mole fraction) incubated with living HEK cells at 37 °C at 500 μM total lipid concentration. The liposomes were labelled with 0.5 mol% Atto647-DOPE (cyan) and incubation was done with the presence of 10 μM SRB (red). Note that the left and right sides of the chamber are slightly misaligned, thus resulting in a slight change in focus.

**Movie S3.** Fusion-mediated intracellular delivery of cargo is a fast process. The sequence corresponds to the cell in Figure 5 D in the main manuscript.

**Movie S4.** Living HEK cells observed for over 30 minutes when incubated with 10 μM SRB at 37 °C. Note that SRB is not internalized by, nor does it have affinity for the cells within the observed period.

**Movie S5.** Living HEK cells incubated with 100 μM (total lipid concentration) of DOTAP:DOPE liposomes (50:50 mole fraction) labelled with 0.5 mol% Bodipy C_16_ (green). Bodipy C_16_ quickly redistributes withing intracellular organelles. Note that the cell morphology is similar to that of control cells that have not been in contact with the liposomes. Incubation was carried out at 37 °C.

**Movie S6.** Living HEK cells incubated with 400 μM (total lipid concentration) of DOTAP:DOPE liposomes (50:50 mole fraction) labelled with 0.5 mol% Bodipy C_16_ (green). Note that strong dye signal in the intracellular organelles at this higher liposomal concentration and some changes in cellular morphology. No cells became permeable to SRB. Incubation was carried out at 37 °C.

**Movie S7.** Living HEK cells incubated with 500 μM (total lipid concentration) of DOTAP:DOPE liposomes (50:50 mole fraction) labelled with 0.5 mol% Bodipy C_16_ (green). In addition to extensive morphological changes, several cells become permeabilized to SRB. Incubation was carried out at 37 °C.

**Movie S8.** FLIM images of control living HEK cells labelled with FliptR (1 μM medium concentration) observed for 30 minutes at 37 °C. Note the stable membrane labelling at constant lifetime throughout the measurements.

**Movie S9.** FLIM images of living HEK cells labelled with FliptR (1 μM medium concentration) observed for 30 minutes at 37 °C upon incubation with non-labelled 500 μM DOTAP:DOPE (50:50 mole fraction) liposomes. Note the appearance of surface and intracellular materials that exhibit a short lifetime that coincided with FliptR recruitment from the PM. PM lifetime is also reduced.

**Movie S10.** Living HEK cells incubated with 500 μM DOTAP:DOPE:DOPC (20:50:30 mole fraction) liposomes labelled with 0.5 mol% Atto647-DOPE that had been pre-incubated in 10x diluted serum. Cell incubation was performed in the presence of 10 μM SRB. Both the liposomes and SRB are co-internalized by cells.

**Movie S11.** Living HEK cells incubated with 500 μM MVL5:DOPE:DOPC (5:50:45 mole fraction) liposomes labelled with 0.5 mol% Atto647-DOPE that had been pre-incubated in 10x diluted serum. Cell incubation was performed in the presence of 10 μM SRB. Both the liposomes and SRB are co-internalized by cells.

**References SI**

1. Lira, R. B., Robinson, T., Dimova, R. & Riske, K. A. Highly Efficient Protein-free Membrane Fusion: A Giant Vesicle Study. *Biophys J* **116**, 79–91 (2019).

2. Salvati, A. *et al.* Experimental and theoretical comparison of intracellular import of polymeric nanoparticles and small molecules: Toward models of uptake kinetics. *Nanomedicine* **7**, 818–826 (2011).

3. Tenuta, T. *et al.* Elution of labile fluorescent dye from nanoparticles during biological use. *PLoS One* **6**, (2011).
